# Mapping anorexia nervosa genes to clinical phenotypes

**DOI:** 10.1017/S0033291721004554

**Published:** 2023-04

**Authors:** Jessica S. Johnson, Alanna C. Cote, Amanda Dobbyn, Laura G. Sloofman, Jiayi Xu, Liam Cotter, Alexander W. Charney, Eating Disorders Working Group of the Psychiatric Genomics Consortium, Andreas Birgegård, Jennifer Jordan, Martin Kennedy, Mikaél Landén, Sarah L. Maguire, Nicholas G. Martin, Preben Bo Mortensen, Laura M. Thornton, Cynthia M. Bulik, Laura M. Huckins

**Affiliations:** 1 Pamela Sklar Division of Psychiatric Genomics, Icahn School of Medicine at Mount Sinai, New York, NY 10029, USA; 2 Department of Genetics and Genomics, Icahn School of Medicine at Mount Sinai, New York, NY 10029, USA; 3 Icahn Institute for Genomics and Multiscale Biology, Icahn School of Medicine at Mount Sinai, New York, NY 10029, USA; 4 Seaver Autism Center for Research and Treatment, Icahn School of Medicine at Mount Sinai, New York, NY 10029, USA; 5 Department of Psychiatry, Icahn School of Medicine at Mount Sinai, New York, NY 10029, USA; 6 James J. Peters Department of Veterans Affairs Medical Center, Mental Illness Research, Education and Clinical Centers, Bronx, NY 14068, USA; 7 Department of Medical Epidemiology and Biostatistics, Karolinska Institutet, Stockholm, Sweden; 8 Department of Psychological Medicine, Christchurch School of Medicine & Health Sciences, University of Otago, 2 Riccarton Avenue, PO Box 4345, 8140 Christchurch, New Zealand; 9 Institute of Neuroscience and Physiology, Sahlgrenska Academy at Gothenburg University, SE-413 45 Gothenburg, Sweden; 10 InsideOut Institute, University of Sydney, New South Wales 2006, Australia; 11 QIMR Berghofer Medical Research Institute, Locked Bag 2000, Royal Brisbane Hospital, Herston, QLD 4029, Australia; 12 The Lundbeck Foundation Initiative for Integrative Psychiatric Research, Aarhus, Denmark; 13 National Centre for Register-Based Research, Aarhus University, Aarhus, Denmark; 14 Department of Psychiatry, University of North Carolina at Chapel Hill, Chapel Hill, NC 27517, USA; 15 Department of Nutrition, University of North Carolina at Chapel Hill, Chapel Hill, NC 27517, USA

**Keywords:** Anorexia nervosa, EHR, pheWAS, PrediXcan, transcriptomic imputation

## Abstract

**Background:**

Anorexia nervosa (AN) is a psychiatric disorder with complex etiology, with a significant portion of disease risk imparted by genetics. Traditional genome-wide association studies (GWAS) produce principal evidence for the association of genetic variants with disease. Transcriptomic imputation (TI) allows for the translation of those variants into regulatory mechanisms, which can then be used to assess the functional outcome of genetically regulated gene expression (GReX) in a broader setting through the use of phenome-wide association studies (pheWASs) in large and diverse clinical biobank populations with electronic health record phenotypes.

**Methods:**

Here, we applied TI using S-PrediXcan to translate the most recent PGC-ED AN GWAS findings into AN-GReX. For significant genes, we imputed AN-GReX in the Mount Sinai Bio*Me*™ Biobank and performed pheWASs on over 2000 outcomes to test the clinical consequences of aberrant expression of these genes. We performed a secondary analysis to assess the impact of body mass index (BMI) and sex on AN-GReX clinical associations.

**Results:**

Our S-PrediXcan analysis identified 53 genes associated with AN, including what is, to our knowledge, the first-genetic association of AN with the major histocompatibility complex. AN-GReX was associated with autoimmune, metabolic, and gastrointestinal diagnoses in our biobank cohort, as well as measures of cholesterol, medications, substance use, and pain. Additionally, our analyses showed moderation of AN-GReX associations with measures of cholesterol and substance use by BMI, and moderation of AN-GReX associations with celiac disease by sex.

**Conclusions:**

Our BMI-stratified results provide potential avenues of functional mechanism for AN-genes to investigate further.

## Introduction

Anorexia nervosa (AN) is a severe eating disorder with a lifetime prevalence of 0.9–4%, characterized by extreme low body weight, fear of gaining weight, and compensatory weight-loss behaviors such as dietary restrictions, purging, and excessive exercise (Zipfel, Giel, Bulik, Hay, & Schmidt, 2015). Despite having one of the highest mortality rate of any psychiatric disorder and an increased risk of suicide (Bulik, Flatt, Abbaspour, & Carroll, 2019; Chesney, Goodwin, & Fazel, 2014; Mitchell & Peterson, 2020), relatively little is known about the biological mechanisms underlying AN, and effective, evidence-based treatments are scant, especially for adults (Watson & Bulik, 2013). Twin studies have established AN-heritability between 50% and 60%, indicating a considerable contribution of genetic factors to disease liability (Watson et al., 2019; Yilmaz, Hardaway, & Bulik, 2015).

Genetic studies of AN provide evidence of both psychiatric and metabolic etiology. The largest AN genome-wide association study (GWAS) to date (*N*_Cases_ = 16 992) uncovered eight AN-associated loci (Watson et al., 2019), and determined single-nucleotide polymorphism (SNP)-based heritability (*h*^2^_SNP_) of 11–17%, similar to other psychiatric disorders. In addition, genetic correlations demonstrate significant shared genetic variation between AN and other psychiatric disorders, including major depressive disorder (MDD), obsessive-compulsive disorder, anxiety disorders (Watson et al., 2019), schizophrenia (Duncan et al., 2017; Watson et al., 2019), alcohol use disorder (Munn-Chernoff et al., 2021), as well as with physical activity (Hübel et al., 2019a; Watson et al., 2019). Significant negative genetic correlation between AN and anthropometric and metabolic traits [including body mass index (BMI), fat mass, obesity, type-2 diabetes, leptin, and insulin-related traits; Duncan et al., 2017; Hübel et al., 2019a; Watson et al., 2019] have also been observed, further indicating metabolic components to AN disease risk. Studies of AN polygenic risk scores indicate additional genetic associations of AN risk variants with anthropometric, behavioral, and psychiatric traits (Hübel et al., 2021), and with weight trajectories even among healthy adults (Xu et al., 2021).

Although GWASs may provide powerful insights into genetic associations with AN, they cannot pinpoint gene- or tissue-level associations. Therefore, in this study we apply transcriptomic imputation (TI) to identify tissue-specific gene associations with AN. TI approaches leverage gene expression predictor models derived from large, well-curated gene expression datasets [e.g. the Genotype-Tissue Expression project (GTEx) (Lonsdale et al., 2013), CommonMind Consortium (CMC) (Fromer et al., 2016; Huckins et al., 2019), and Depression Genes and Networks (DGN) (Battle et al., 2014)]. These models may be applied to predict genetically regulated gene expression (GReX) in large genotyped cohorts, without the need to collect tissue samples (Huckins et al., 2019).

Observational genetic studies are powerful approaches to detect disease-associated variants, but cannot address subthreshold or prodromal disease states, and do not speak to clinical relevance. Therefore, we leverage a phenome-wide association study (pheWAS) design to probe clinical outcomes with AN-associations. pheWAS effectively query the full electronic health record (EHR) to identify diagnoses and traits associated with a gene or variant (Denny et al., 2010; Pendergrass et al., 2011; Smoller, 2018), and have been used to replicate associations and identify new pleiotropic consequences of GWAS variants, including psychiatric disorders (Denny et al., 2013; Leppert et al., 2020; Zheutlin et al., 2019). EHRs contain vast amount of longitudinal health data, including diagnoses, medications, laboratory tests, vital signs, and family medical history, and, coupled with genetic data through hospital-based biobanks, can provide an understanding of the clinical spectrum of disease and disease progression across the lifetime of the patient. Exploring the associations of AN-genetics with clinical phenotypes has the potential to clarify how some of these GWAS variants functionally contribute to AN disease risk, symptomatology, and clinical presentation.

Here, we explored the associations between AN-GReX and the clinical phenome. We performed TI using S-PrediXcan on the most recent PGC-ED AN GWAS (Watson et al., 2019) to first find GReX associated with AN, and then tested for clinical associations of these genes with structured EHR-encoded phenotypes using pheWAS. We further investigated the effects of BMI and sex on the GReX-phenotype associations through stratification of biobank individuals. Understanding the clinical associations of aberrant gene expression across the phenome may clarify the biological mechanisms of relevant AN GWAS risk variants.

## Methods

### Transcriptomic imputation

We performed TI using S-PrediXcan (Barbeira et al., 2018) on the largest available summary statistics of AN (16 992 cases/55 525 controls) (Watson et al., 2019). We tested for the association of GReX using available GTEx (Lonsdale et al., 2013), DGN (Battle et al., 2014), and CMC (Fromer et al., 2016; Hoffman et al., 2019) predictor models (Barbeira et al., 2018; Gamazon et al., 2015; Huckins et al., 2019) across 50 tissues with AN case–control status. We established two thresholds for significance; first, Bonferroni correction for all genes tested within each tissue (*p*_tissue_, online Supplementary Table S1), and second, correcting for all tissues and genes tested (*p*_Experiment_ = 3.75 × 10^−8^). We performed a gene-set analysis for 53 significant AN-genes using FUMA (v1.3.6) GENE2FUNC (Watanabe, Taskesen, van Bochoven, & Posthuma, 2017), including all S-PrediXcan genes (*N* = 28 454) as the background genes. We defined significant gene sets using *p*_FDR_ < 0.05.

### Bio*Me*™

The Mount Sinai Bio*Me*™ Biobank includes genotype and EHR data from 31 704 individuals (online Supplementary Table S2). Individuals were genotyped on the Illumina Global Screening Array; quality control (QC), and imputation of the genotyping data for Bio*Me*™ is described elsewhere (Zheutlin et al., 2019). Ancestry QC of Bio*Me*™ individuals using principal component analysis in PLINK (Chang et al., 2015; Purcell et al., 2007) is described in the online Supplemental Methods. After QC, a total of 31 585 individuals were available for analysis.

### pheWAS

We calculated GReX for all S-PrediXcan *p*_tissue_ significant genes (*N* = 53) across 45 tissues from GTEx, DGN, and CMC in Bio*Me*™ using PrediXcan-2 (Barbeira et al., 2018; Gamazon et al., 2015). We excluded sex-specific tissues (ovary, uterus, vagina, prostate, and testis) from this analysis, and genes with GReX variance (gVAR) less than 0.002 (online Supplementary Table S3C).

Logistic regressions between AN-GReX and categorical phenotypes were performed using the pheWAS R package (Carroll, Bastarache, & Denny, 2014), adjusting for age, sex, and the first five genotype-derived principal components. pheWAS were run individually for each Bio*Me*™ ancestry cohort, and results were meta-analyzed using an inverse-variance based approach in METAL (Willer, Li, & Abecasis, 2010). We required at least 10 occurrences of each phenotype in each population group for inclusion in the analysis, and an overall effective sample size *N*_eff_ > 100 (Equation (1)). We excluded associations with significant Cochran's *Q*-test for heterogeneity scores across cohorts (pHet > 0.001).

Effective sample size (*N*_eff_):1
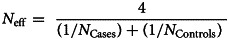


‘Encounter Diagnoses’ were recorded at each patient visit using the International Classification of Disease (ICD) coding system. Phecodes were assigned from Encounter Diagnoses by grouping ICD-9 and ICD-10 diagnostic codes (Wu et al., 2019). We defined cases as individuals with at least two counts of a code. Those with zero counts were considered ‘controls’, and those with only one count were set to missing. After QC, our dataset included 2178 unique Encounter Diagnosis codes and 1093 unique phecodes. Due to the high correlation between Encounter Diagnosis and phecode files, we combined all results from both pheWAS and performed an FDR correction in R to determine significance (*p*_FDR_ < 0.05).

In addition to diagnostic codes, Bio*Me*™ EHR data include allergies, vital signs (weight, height, blood pressure, pulse, pulse oximetry, respirations, and temperature), lab results, family history, personal history, medication use, obstetrics and gynecology outcomes (OB/GYN), and other social and behavioral traits. Summaries, descriptive statistics, and QC descriptions for these phenotypes can be found in online Supplementary Methods. We tested for AN-GReX-pheWAS associations within each category, establishing significance using a Bonferroni correction (Equation (2)).

Bonferroni-correction for EHR data at tissue- and experiment-significance thresholds:2
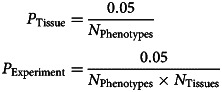


### Stratification by BMI

Given the nature of AN symptomology, we tested whether our significant AN-GReX associations varied based on BMI. We stratified Bio*Me*™ individuals into three BMI categories: low (<first BMI quartile, *N* = 6976), mid (first—third BMI quartiles, *N* = 13 972) and high (>third BMI quartile, *N* = 9350; online Supplementary Fig. S1A). Quartiles were defined based on ancestry- and sex-specific BMI distributions within our data (online Supplementary Fig. S1B; Table S2; Supplemental Methods). We repeated our pheWAS within these categories, for all AN-GReX associations reaching significance in our overall analysis. BMI-stratified pheWAS were adjusted for age, sex, and the first five genotype-derived PCs.

### Stratification by sex

To determine whether our associations varied based on sex, we stratified the Bio*Me*™ cohort by females (*N* = 17 968) and males (*N* = 12 417), with sex determined by genotype, and repeated our AN-GReX pheWAS analyses within each group. Here, we included sex-specific tissues (ovary, uterus, vagina, prostate, and testis) for the respective sex. Sex-stratified pheWAS were adjusted for age and the first five genotype-derived PCs.

### Testing for hidden case contamination

It is possible that some of the clinical associations within our study stem from undiagnosed ED and AN cases, rather than a direct effect of gene expression on phenomic expression. We term this ‘diagnostic contamination’. To test whether this effect may drive the associations we observe, we simulated the effects of diagnostic contamination occurring at rate *p* within our biobank sample, with effect *β* on GReX.

Full derivations and simulations are shown in our online Supplementary Material; briefly, we derive the expression among controls, and cases contaminated at rate *p*; the difference in expression between the two groups; the expected variance among cases, and pooled across all samples, and the expected statistical significance (*T*-score, and *p* value). Next, we simulated gene expression distributions for (i) 1000 cases and 1000 controls; (ii) 1000 cases and 10 000 controls; (iii) 1000 cases and 30 000 controls; each for a range of *p* (0.1, 0.5, 1, 2, 5, 10, 20, 30, 40, 50%) and *β* (1/10, 1/5, 1/4, 1/3, 1/2, 1, 2, 3, 4, 5, 10) values. We repeated our simulations 10 000 times at each case–control proportion- *p*- *β* combination, and demonstrated that our formulae accurately estimate the desired values (online Supplementary Fig. S2).

In order to test whether diagnostic contamination may account for the associations observed within our pheWAS, we calculated the expected impact of diagnostic contamination for two genes (*NCKIPSD*-Aorta; *SEMA3F*-Spinal Cord) with the largest effect sizes *β* observed in our S-PrediXcan analysis, under two scenarios:

1. Diagnostic contamination occurs among our cases only, assuming the normal population rate for AN: 0.9% among women; 0.3% among men.
2. Contamination occurs at significantly higher levels than might be expected in the population (0.6, 1.2, 3, 6%), and all of these samples fall into our case category.

For each scenario, we repeated our calculation using 500 and 1000 cases, matched with 1000, 5000, and 30 000 controls.

## Results

### S-PrediXcan identifies gene–tissue associations with AN

**Fig. 1.**
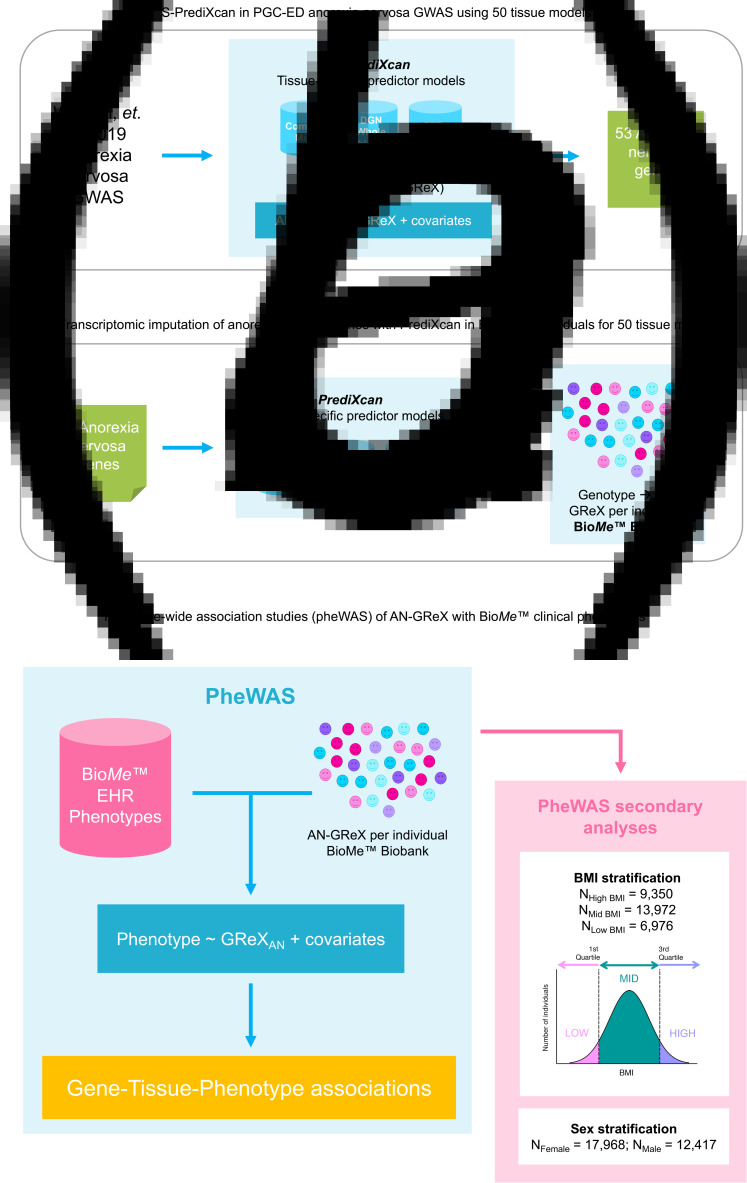
Graphical depiction of S-PrediXcan and PrediXcan TI and pheWAS analyses. (*a*) We used S-PrediXcan predictor models for 50 different tissue types to impute GReX in Watson et al. (2019) AN GWAS and found 53 genes whose GReX was associated with AN. We then (*b*) imputed GReX for those 53 AN-genes in our Bio*Me*™ cohort and (*c*) performed a pheWAS across available EHR phenotypes. The pheWAS analyses were run within each ancestral population and then meta-analyzed using an inverse-variance approach in METAL. Secondary analyses included stratifying individuals in Bio*Me*™ by BMI and sex, and running the pheWAS analyses within each stratification group.

**Fig. 2.**
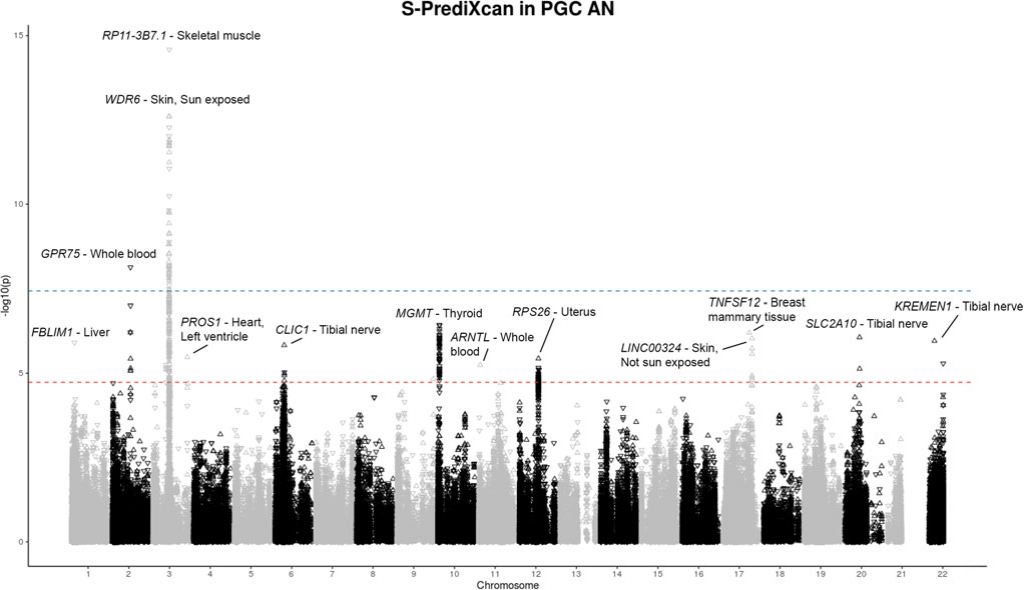
S-PrediXcan results for PGC-ED AN GWAS (2019) (*N*_Cases_ = 16 992, *N*_Controls_ = 55 525). S-PrediXcan TI of the PGC-ED AN GWAS summary statistics to determine GReX, with tests for association of GReX with AN disease status. Manhattan plot of S-PrediXcan gene–tissue associations with AN for 50 tissues. Each point represents a different gene–tissue association result; i.e. the same gene may have multiple points within a peak. Experiment-wide significance threshold of *p* < 3.75 **×** 10**^−^**^8^ (solid line); tissue-specific significance threshold of *p* < 2.45 × 10**^−^**^5^ (dotted line).

We applied S-PrediXcan to PGC-AN GWAS summary statistics, and identified 218 gene–tissue associations, including 53 unique genes across 12 loci (Fig. 1). Two loci on chromosomes 2 and 3 reached experiment-wide significance (*p* < 3.75 × 10^−8^), while 10 loci reached tissue-specific significance (Fig. 2, Table 1; online Supplementary Table S3A). Our S-PrediXcan AN-genes are significantly enriched in gene sets for traits including inflammatory bowel disease (IBD: Crohn's and ulcerative colitis) (*p*_adj_ = 1.3 × 10^−25^), sleep duration (*p*_adj_ = 6.9 × 10^−17^), blood protein levels (*p*_adj_ = 3.2 × 10^−16^), intelligence (*p*_adj_ < 1.5 × 10^−6^), regular attendance at a religious group, gym, or sports club (*p*_adj_<1.46 × 10^−10^), mood instability (*p*_adj_ = 3.3 × 10^−9^), AN (*p*_adj_ = 7.4 × 10^−8^), and body fat distribution (*P*_adj_ < 5.0 × 10^−7^) (online Supplementary Table S4A and B).

**Table 1.** S-PrediXcan loci results

Chromosome	Genes	*Z* score	*p* value	Top finding (gene)	Top finding (tissue)
1	*FBLIM1*	−4.851060	1.23 × 10^−6^	*FBLIM1*	Liver
2	*ASB3, CHAC2, GPR75*	−5.780228	**7.46 × 10^−9^**	*GPR75*	Whole Blood
3	*SPINK8, PFKFB4, TMEM89, SLC26A6, CELSR3, NCKIPSD, ARIH2OS, ARIH2, P4HTM, WDR6, DALRD3, NDUFAF3, USP19, LAMB2, CCDC71, CCDC36, C3orf62, GPX1, NICN1, DAG1, APEH, MST1, MST1R, RNF123, RBM6, RBM5, SEMA3F, TUSC2*	7.321127	**2.46 × 10^−^^13^**	*WDR6*	Skin, Sun Exposed
3	*PROS1, DHFRL1*	4.687382	2.77 × 10^−6^	*DHFRL1*	DGN Whole Blood
6	*CLIC1*	4.814629	1.47 × 10^−6^	*CLIC1*	Tibial Nerve
10	*MGMT, EBF3*	−5.086391	3.65 × 10^−7^	*MGMT*	Thyroid
11	*ARNTL*	4.534957	5.76 × 10^−6^	*ARNTL*	Whole Blood
12	*SUOX, RPS26, SLC26A10*	4.628307	3.69 × 10^−6^	*RPS26*	Uterus
17	*TNFSF12, LINC00324*	4.982641	6.27 × 10^−7^	*TNFSF12*	Breast Mammary Tissue
20	*SLC2A10*	4.921176	8.60 × 10^−7^	*SLC2A10*	Tibial Nerve
22	*KREMEN1*	4.872670	1.10 × 10^−6^	*KREMEN1*	Tibial Nerve
22	*SCO2*	−4.560015	5.11 × 10^−6^	*SCO2*	Brain, Cerebelium

Summary of S-PrediXcan of PGC-ED AN GWAS results. Genes associated with AN from each locus are shown, as well as the top finding *Z* score, *p* value, gene and tissue for each locus. *p* values shaded in bold indicate experiment-wide significant loci (*p* < 3.75 × 10^−8^).

### AN-GReX is associated with autoimmune and autoinflammatory diseases

**Fig. 3.**
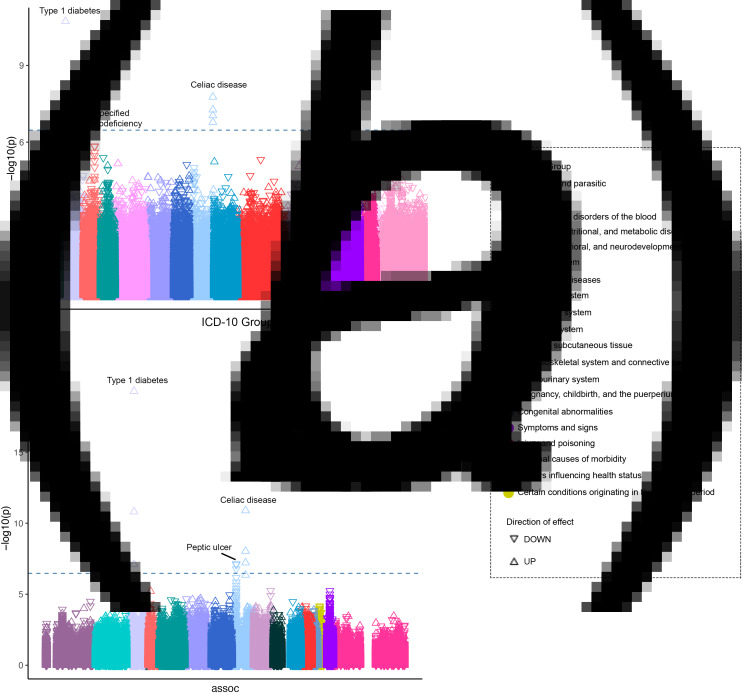
AN-GReX associations with Bio*Me*™ Diagnosis codes. GReX-Tissue-Phenotype associations for (*a*) encounter diagnosis ICD-10 codes (*N* = 2178) and (*b*) phecodes (*N* = 1093) for the Bio*Me*™ cohort (*N* = 30 585). Diagnosis codes are plotted along the *x*-axis and grouped by category, with the −log(10) *p* value associations along the *y*-axis. FDR-significant diagnosis codes are labeled (FDR-adjusted *p* < 0.05). FDR-significant *p* value threshold *p* = 3.4 **×** 10**^−^**^7^ (blue dashed line).

We performed a pheWAS to identify clinical associations with dysregulation of our AN-genes. We identified 16 FDR-significant gene–tissue associations with four Phecode- and Encounter Diagnosis phenotypes (FDR < 0.05): type 1 diabetes (*N*_Cases_ = 408), celiac disease (*N*_Cases_ = 63), peptic ulcer (*N*_Cases_ = 254), and unspecified immunodeficiency (*N*_Cases_ = 55) (Fig. 3; online Supplementary Table S5). These associations are driven in part by the MHC-gene *CLIC1*; predicted upregulation of *CLIC1* in spleen was associated with celiac disease and type 1 diabetes (*p* = 1.30 × 10^−11^, *p* = 5.08 × 10^−20^, respectively) in the overall cohort. We did not observe any significant effect of patient BMI group on these associations (online Supplementary Fig. S3A and C). Upregulation of *CLIC1* in females was significantly associated with type 1 diabetes (*N*_Cases_ = 230) and celiac disease (*N*_Cases_ = 53) (Females-Spleen-*CLIC1*, *p* < 1.49 × 10^−9^) (online Supplementary Fig. S3B and D) .

**Table 2.** pheWAS AN-GReX tissue associations with cholesterol phenotypes

Stratification category	Gene	Tissue	Effect	s.e.	*p*
(A) AN-GReX pheWAS associations with highest recorded total cholesterol (mg/dl)					
High BMI	***MGMT***	**Stomach**	**−7.2**	**1.4**	**1.6 × 10^−7^**
High BMI	***MGMT***	**Brain, nucleus accumbens basal ganglia**	**−11.5**	**2.3**	**4.0 × 10^−7^**
High BMI	***MGMT***	**Colon, transverse**	**−6.1**	**1.3**	**1.2 × 10^−6^**
High BMI	***MGMT***	**Brain, caudate basal ganglia**	**−7.2**	**1.5**	**2.1 × 10^−6^**
High BMI	***MGMT***	**Cells, EBV-transformed lymphocytes**	**−8.0**	**1.7**	**2.8 × 10^−6^**
High BMI	***MGMT***	**Thyroid**	**−5.6**	**1.2**	**2.9 × 10^−6^**
High BMI	***MGMT***	**Visceral omentum adipose**	**−5.7**	**1.2**	**2.9 × 10^−6^**
High BMI	***MGMT***	**CommonMind DLPFC**	**−13.6**	**2.9**	**3.7 × 10^−6^**
High BMI	***MGMT***	**Lung**	**−5.9**	**1.3**	**6.3 × 10^−6^**
High BMI	***MGMT***	**Esophagus, mucosa**	**−5.6**	**1.3**	**9.3 × 10^−6^**
High BMI	***MGMT***	**Brain, hippocampus**	**−7.6**	**1.7**	**1.0 × 10^−5^**
High BMI	***MGMT***	**Brain, cerebellum**	**−7.8**	**1.8**	**1.9 × 10^−5^**
High BMI	***MGMT***	**Spleen**	**−3.7**	**0.9**	**2.7 × 10^−5^**
High BMI	***MGMT***	**Tibial nerve**	**−5.2**	**1.3**	**3.7 × 10^−5^**
High BMI	*MGMT*	Liver	−5.5	1.4	1.3 × 10^−4^
High BMI	*MGMT*	Brain, cerebellar hemisphere	−4.2	1.2	2.9 × 10^−4^
Overall	*MGMT*	Stomach	−2.7	0.7	2.9 × 10^−4^
(B) AN-GReX pheWAS associations with highest recorded LDL cholesterol (mg/dl)					
High BMI	***MGMT***	**Stomach**	**−5.2**	**1.1**	**2.6 × 10^−6^**
High BMI	***MGMT***	**Brain, nucleus accumbens basal ganglia**	**−7.9**	**1.8**	**1.5 × 10^−5^**
High BMI	***MGMT***	**Brain, hippocampus**	**−5.8**	**1.4**	**3.1 × 10^−5^**
High BMI	***MGMT***	**Colon, transverse**	**−4.1**	**1.0**	**4.0 × 10^−5^**
High BMI	***MGMT***	**Cells, EBV-transformed lymphocytes**	**−5.7**	**1.4**	**4.2 × 10^−5^**
High BMI	***MGMT***	**Brain, caudate basal ganglia**	**−4.9**	**1.2**	**7.4 × 10^−5^**
High BMI	***MGMT***	**Thyroid**	**−3.8**	**1.0**	**7.4 × 10^−5^**
High BMI	***MGMT***	**Visceral omentum adipose**	**−3.8**	**1.0**	**9.4 × 10^−5^**
High BMI	***MGMT***	**CommonMind DLPFC**	**−9.1**	**2.4**	**1.2 × 10^−4^**
High BMI	***MGMT***	**Esophagus, mucosa**	**−3.9**	**1.0**	**1.2 × 10^−4^**
High BMI	*MGMT*	Lung	−3.8	1.1	3.5 × 10^−4^
High BMI	*MGMT*	Brain, cerebellum	−5.2	1.5	4.1 × 10^−4^
High BMI	*MGMT*	Tibial nerve	−3.5	1.0	6.3 × 10^−4^
High BMI	*MGMT*	Liver	−3.8	1.1	8.2 × 10^−4^
Mid BMI	*SLC26A10*	Aorta artery	−4.7	1.4	8.8 × 10^−4^
(C) AN-GReX pheWAS associations with highest recorded HDL cholesterol (mg/dl)					
Overall	*SUOX*	Thyroid	0.9	0.3	1.7 × 10^−4^
Overall	*SUOX*	Brain, caudate basal ganglia	1.4	0.4	1.9 × 10^−4^
Overall	*RPS26*	Breast mammary tissue	−0.7	0.2	1.9 × 10^−4^
Overall	*SUOX*	Tibial artery	1.4	0.4	2.0 × 10^−4^
Overall	*RPS26*	Brain, nucleus accumbens basal ganglia	−0.7	0.2	3.1 × 10^−4^
Overall	*SUOX*	Tibial nerve	0.8	0.2	3.5 × 10^−4^
Overall	*SUOX*	Pituitary	1.0	0.3	4.6 × 10^−4^
Female	*SUOX*	Brain, caudate basal ganglia	1.8	0.5	4.7 × 10^−4^
Overall	*SUOX*	Brain, anterior cingulate cortex (BA24)	2.0	0.6	5.1 × 10^−4^
Overall	*RPS26*	CommonMind DLPFC	−1.2	0.3	5.2 × 10^−4^
Overall	*RPS26*	Skeletal muscle	−0.6	0.2	5.4 × 10^−4^
Overall	*SUOX*	Esophagus, mucosa	4.2	1.2	5.5 × 10^−4^
Overall	*SUOX*	Skin, not sun exposed	1.2	0.3	6.1 × 10^−4^
Overall	*RPS26*	Brain, putamen basal ganglia	−0.7	0.2	6.3 × 10^−4^
Overall	*RPS26*	Minor salivary gland	−1.0	0.3	6.5 × 10^−4^
Overall	*SUOX*	Small intestine, terminal ileum	1.3	0.4	6.8 × 10^−4^
Overall	*SUOX*	Esophagus, muscularis	1.4	0.4	7.6 × 10^−4^
Overall	*RPS26*	Tibial nerve	−0.6	0.2	7.6 × 10^−4^
Overall	*SUOX*	Lung	1.0	0.3	7.7 × 10^−4^
Overall	*SUOX*	Pancreas	1.0	0.3	7.9 × 10^−4^
Overall	*RPS26*	Brain, substantia nigra	−0.9	0.3	8.0 × 10^−4^
Overall	*RPS26*	Brain, anterior cingulate cortex (BA24)	−0.6	0.2	8.1 × 10^−4^
Overall	*RPS26*	Brain, hypothalamus	−0.7	0.2	8.1 × 10^−4^
Overall	*SUOX*	Heart, left ventricle	2.2	0.7	8.1 × 10^−4^
Overall	*RPS26*	Aorta artery	−0.7	0.2	8.2 × 10^−4^
Overall	*RPS26*	Brain, spinal cord (cervical c-1)	−0.6	0.2	8.5 × 10^−4^
Overall	*SUOX*	Spleen	0.9	0.3	8.8 × 10^−4^
Overall	*SUOX*	Breast mammary tissue	3.7	1.1	9.2 × 10^−4^
Overall	*RPS26*	Whole blood	−0.7	0.2	9.5 × 10^−4^
Overall	*RPS26*	Lung	−0.6	0.2	9.9 × 10^−4^
(D) AN-GReX pheWAS associations with mean total cholesterol (mg/dl)					
High BMI	***MGMT***	**CommonMind DLPFC**	**−9.5**	**2.1**	**4.8 × 10^−6^**
High BMI	***MGMT***	**Brain, spinal cord (cervical c-1)**	**−4.9**	**1.1**	**6.6 × 10^−6^**
High BMI	***MGMT***	**Brain, caudate basal ganglia**	**−4.8**	**1.1**	**7.9 × 10^−6^**
High BMI	***MGMT***	**Brain, hippocampus**	**−5.2**	**1.2**	**1.8 × 10^−5^**
High BMI	***MGMT***	**Lung**	**−3.9**	**0.9**	**2.2 × 10^−5^**
High BMI	***MGMT***	**Esophagus, mucosa**	**−3.7**	**0.9**	**2.6 × 10^−5^**
High BMI	***MGMT***	**Brain, cerebellum**	**−5.1**	**1.3**	**6.8 × 10^−5^**
High BMI	***MGMT***	**Liver**	**−3.9**	**1.0**	**9.1 × 10^−5^**
High BMI	*MGMT*	Spleen	−2.4	0.6	1.4 × 10^−4^
High BMI	*APEH*	Skin, sun exposed	8.9	2.6	5.8 × 10^−4^
(E) AN-GReX pheWAS associations with mean LDL cholesterol (mg/dl)					
High BMI	***MGMT***	**Brain, hippocampus**	**−4.1**	**1.0**	**2.6 × 10^−5^**
High BMI	***MGMT***	**CommonMind DLPFC**	**−7.1**	**1.7**	**4.1 × 10^−5^**
High BMI	***MGMT***	**Brain, spinal cord (cervical c-1)**	**−3.7**	**0.9**	**4.2 × 10^−5^**
High BMI	***MGMT***	**Esophagus, mucosa**	**−2.9**	**0.7**	**1.0 × 10^−4^**
High BMI	***MGMT***	**Brain, caudate basal ganglia**	**−3.4**	**0.9**	**1.2 × 10^−4^**
High BMI	*MGMT*	Brain, cerebellum	−3.9	1.1	2.8 × 10^−4^
High BMI	*MGMT*	Lung	−2.8	0.8	2.8 × 10^−4^
High BMI	*MGMT*	Liver	−3.0	0.8	3.1 × 10^−4^
Mid BMI	*SLC26A10*	Aorta artery	−3.4	1.0	7.8 × 10^−4^
(F) AN-GReX pheWAS associations with mean HDL cholesterol (mg/dl)					
Mid BMI	*RP11-3B7.1*	Lung	−2.1	0.6	4.2 × 10^−4^
Male	*RP11-3B7.1*	Lung	−2.0	0.6	4.9 × 10^−4^
Overall	*RP11-3B7.1*	Lung	−1.5	0.4	6.2 × 10^−4^
Male	*GPX1*	Brain, caudate basal ganglia	1.2	0.3	8.3 × 10^−4^
Overall	*RPS26*	Brain, nucleus accumbens basal ganglia	−0.5	0.1	9.4 × 10^−4^
Overall	*RPS26*	Breast mammary tissue	−0.5	0.1	9.4 × 10^−4^
(G) AN-GReX pheWAS associations with lowest total cholesterol (mg/dl)					
Female	*PFKFB4*	Skin, sun exposed	5.7	1.7	7.4 × 10^−4^
High BMI	*MGMT*	CommonMind DLPFC	−7.3	2.2	7.5 × 10^−4^
High BMI	*MST1*	Stomach	5.0	1.5	8.7 × 10^−4^
(H) AN-GReX pheWAS associations with lowest LDL cholesterol (mg/dl)					
Mid BMI	*RBM5*	Pituitary	8.3	2.5	7.2 × 10^−4^
Overall	*MST1*	Liver	1.4	0.4	8.6 × 10^−4^
(I) AN-GReX pheWAS associations with lowest HDL cholesterol (mg/dl)					
High BMI	*RP11-477N3.1*	Lung	4.2	1.0	3.4 × 10^−5^
Female	*SEMA3F*	Spleen	3.3	0.9	3.9 × 10^−4^
High BMI	*RP11-477N3.1*	Skeletal muscle	2.4	0.7	3.9 × 10^−4^
Mid BMI	*RP11-3B7.1*	Lung	−2.0	0.6	4.5 × 10^−4^
Overall	*SEMA3F*	Spleen	2.3	0.7	7.0 × 10^−4^
Overall	*RP11-3B7.1*	Lung	−1.4	0.4	8.0 × 10^−4^
High BMI	*RP11-477N3.1*	Tibial artery	2.0	0.6	9.1 × 10^−4^
Mid BMI	*CCDC36*	Lung	−1.7	0.5	9.2 × 10^−4^

pheWAS associations with highest, lowest, and mean total cholesterol, LDL cholesterol and HDL cholesterol measures for all stratification groups: overall (*N* = 23 661), high-BMI (*N* = 6910), mid-BMI (*N* = 11 243), low-BMI (*N* = 5324), females (*N* = 13 907), and males (*N* = 9669). AN-GReX association with phenotypes of (A) highest recorded total cholesterol (mg/dl), (B) highest recorded LDL cholesterol (mg/dl), (C) highest recorded HDL cholesterol (mg/dl), (D) mean total cholesterol (mg/dl), (E) mean LDL cholesterol (mg/dl), (F) mean HDL cholesterol (mg/dl), (G) lowest recorded total cholesterol (mg/dl), (H) lowest recorded LDL cholesterol (mg/dl), and (I) lowest recorded HDL cholesterol (mg/dl). Bonferroni experiment-wide significant associations are marked in bold (*p*_Experiment_ = 0.05/(9 phenotypes × 45 tissues) = 1.23 × 10^−4^). Bonferroni tissue-specific value threshold *p* < 0.0056 (0.05/9 phenotypes).

### BMI moderates the effect of MGMT-GReX on cholesterol levels

**Fig. 4.**
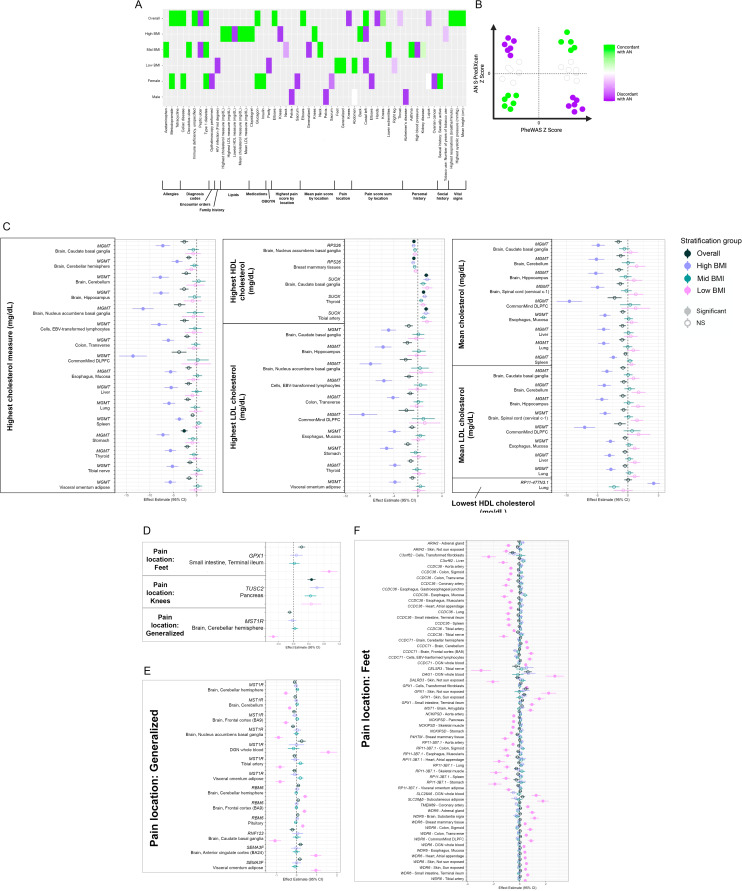
Context-specific pheWAS associations. (*a*) Concordance of context-specific experiment-wide significant AN-GReX clinical associations with AN direction of effect. We compared the direction of effect (DoE) for each experiment-wide gene–tissue-pheWAS association with the DoE of that gene–tissue pair for AN from our S-PrediXcan results (online Supplemental Methods). For those phenotypes concordant with AN, this may indicate that genetic regulation of those AN-genes is more similar to individuals with AN in individuals with those clinical outcomes. (*b*) Schematic of the proportion of concordance of AN-GReX pheWAS associations with AN S-PrediXcan associations. Associations with similar direction of effect to AN (green) identified as ‘concordant’, associations with opposite direction of effect (purple) identified as ‘discordant’. (*c*) Context-specific associations of AN-GReX with lipid phenotypes of highest recorded, lowest recorded, and mean measures of total cholesterol (mg/dl), HDL cholesterol (mg/dl), and LDL cholesterol (mg/dl). Experiment-wide significant threshold set at *p* = 0.05(9 phenotypes × 45 tissues) = 1.2 × 10^−4^. Tissue-specific threshold set at 0.05/(9 phenotypes) = 0.0056. Context-specific associations of AN-GReX with pain location for (*d*) experiment-wide significant associations (*p* = 0.05/(99 phenotypes × 45 tissues) = 1.1 × 10^−5^), (*e*) all context-specific associations (experiment-wide and tissue-specific) with generalized pain, and (*f*) with foot pain. Tissue-specific threshold for pain location set at 0.05/(99 phenotypes) = 5.0 × 10^−4^.

Next, we looked at the association of AN-GReX and measures of highest recorded, lowest recorded and mean total cholesterol (mg/dl), high-density lipoprotein (HDL) cholesterol (mg/dl), and low-density lipoprotein (LDL) cholesterol (mg/dl) (Table 2). In the full cohort, *RPS26* and *SUOX* GReX were significantly associated with the highest HDL measures, and *MGMT* GReX was associated with the highest cholesterol at within-tissue significance (*N* = 23 357) (*p* < 5.1 × 10^−4^) (Fig. 4C). Associations of AN-GReX with lipid measures varied across BMI-stratified groups; predicted downregulation of *MGMT* in the stomach, liver, esophagus, and cells was associated with the highest total cholesterol and LDL levels, and in dorsolateral prefrontal cortex and hippocampus with mean cholesterol and LDL among high-BMI individuals (*N*_High BMI_ = 6910) (High-BMI-Stomach-*MGMT*, *p* = 1.55 × 10^−7^) (Fig. 4C; online Supplementary Table S6).

### AN-GReX is associated with tobacco use

Multiple genes were associated with categorical and continuous measures of tobacco use in the overall cohort at within-tissue significance (online Supplementary Fig. S4, Table S7, Supplementary Results). We found moderating effects of patient BMI on tobacco use: among High-BMI groups, different genes were associated with the number of years of tobacco use at experiment-wide significance (*N*_High BMI_ = 8129) (High-BMI-Pituitary-*CCDC36*, *p* = 4.2 × 10^−6^; High-BMI-Colon, Transverse-*P4HTM*, *p* = 3.4 × 10^−5^; High-BMI-Stomach-*P4HTM*, *p* = 2.2 × 10^−5^) (online Supplementary Fig. S4, Table S7).

### AN-GReX is associated with measures of pain score and pain location

We tested for association between AN-GReX and (1) pain location, (2) pain score overall, and (3) pain score by body location. AN-GReX was associated with multiple measures of pain and location across all stratification groups (Fig. 4D–F; online Supplementary Table S8). Our results revealed a large number of AN-GReX-pain associations specific to the Low-BMI category (Fig. 4D–F; online Supplementary Table S8); in particular, we note 60 gene–tissue associations with presence of foot pain (*N*_Cases_ = 433), one of which passed experiment-wide significance (Low-BMI-Small intestine-*GPX1*, *p* = 7.6 × 10^−6^), which may indicate a propensity to excess exercise, or exercise-related injuries among these individuals.

### Upregulation of *CLIC1* is associated with glucagon medication

Upregulation of *CLIC1* was associated with glucagon, a hormone used to treat severe hypoglycemia, in the overall cohort (*N*_Cases_ = 129) (Overall-Spleen-*CLIC1*, *p* = 4.20 × 10^−9^) and in females (*N*_Cases_ = 78) (Females-Spleen-*CLIC1*, *p* = 1.4 × 10^−8^) (online Supplementary Fig. S5, Table S9). We additionally find significant associations between the upregulation of *CLIC1* and insulin medications in females (Females-Subcutaneous adipose-*CLIC1*, *p* < 9.6 × 10^−7^).

### Identifying potential case contamination effects

Our Bio*Me*™ patients have not all been explicitly assessed for eating disorders, and information regarding eating disorder diagnoses and assessments earlier in life may be omitted from the records due to incomplete clinical history assessments. Therefore, it is possible that diagnostic contamination within some of our sample is responsible for the associations observed within our data. We tested whether such contamination may drive the associations observed in our study, assuming the following different contamination scenarios (online Supplementary Table S10), for two genes with large S-PrediXcan effect sizes: (i) that contamination occurs among cases, assuming the highest common estimate for AN prevalence (0.9% among women, 0.3% among men); (ii) that contamination occurs within our biobank, assuming the highest common estimate for AN prevalence (0.6%, 190 cases), and that all of these samples fall into our ‘case’ category.

Our model indicates that diagnostic contamination among cases is unlikely to result in significant pheWAS associations in our data. Contamination occurring at population ED prevalence (0.6%) did not result in significant associations for any of the case–control scenarios in our model (online Supplementary Table S10); nor did contamination at 1.2% or 3%. Assuming 6% contamination resulted in potentially significant associations (estimated *p* < 2.8 × 10^−8^; see Methods and online Supplementary Methods for more details).

## Discussion

Our analysis identified novel AN-genes associated with metabolic, anthropometric, autoimmune, and psychiatric phenotypes (online Supplementary Table S4B). In particular, the experiment-wide significant locus on chromosome 3 overlaps with a known GWAS peak for IBD (de Lange et al., 2017), and includes genes associated with Crohn's and ulcerative colitis (online Supplementary Table S4B) (Hedman et al., 2019; Mårild et al., 2017; Raevuori et al., 2014; Zerwas et al., 2017), in line with earlier AN GWAS showing genetic overlap with autoimmune disease (Gibson & Mehler, 2019). Variants in one of our significant S-PrediXcan genes, *GPR75*, have been recently shown to have a protective effect against obesity, and have been associated with lower body weight overall (Akbari et al., 2021).

To our knowledge, our results include the first AN-association within the MHC locus (*CLIC1*, chloride intracellular 1), a region that has been associated with many other psychiatric (Ripke et al., 2014; Stahl et al., 2019) and autoimmune disorders. In particular, *CLIC1* has previously been identified as associated with schizophrenia (The Autism Spectrum Disorders Working Group of The Psychiatric Genomics Consortium, 2017), autism (The Autism Spectrum Disorders Working Group of The Psychiatric Genomics Consortium, 2017), MDD (Zhu et al., 2019), post-traumatic stress disorder (Marchese et al., 2021), neuroticism (Baselmans et al., 2019) and depressive phenotypes (Baselmans et al., 2019). Importantly, *CLIC1* variants have also been associated with complement component C4 and C3 protein levels in the blood (Yang et al., 2012), which through the complement cascade (Sekar et al., 2016) are involved in immunological functions of pathogen clearance and in synaptic pruning and neuronal connectivity (Stephan, Barres, & Stevens, 2012). *CLIC1* encodes a chloride ion channel protein involved in many necessary cellular functions including the regulation of cell membrane potential, and the proliferation and differentiation of cells (Li et al., 2018), including a role in axonal outgrowth of neurons (Carlini et al., 2020).

We also identified multiple genes that have been previously associated with decreased sleep and insomnia phenotypes (including a core circadian clock gene *ARNTL*), and with a range of psychiatric disorders (Alloy, Ng, Titone, & Boland, 2017; Boivin, 2000; Bunney & Bunney, 2013; Bunney et al., 2015; Etain et al., 2014; Huckins et al., 2017; Karatsoreos, 2014; Kripke, Mullaney, Atkinson, & Wolf, 1978; Mansour et al., 2009; Melo, Abreu, Linhares Neto, de Bruin, & de Bruin, 2017; Meyrer, Demling, Kornhuber, & Nowak, 2009; Murray, Allen, Trinder, & Burgess, 2002), including eating disorders (Allison, Spaeth, & Hopkins, 2016), as well as with satiety and hunger (Arble, Bass, Laposky, Vitaterna, & Turek, 2009; Herpertz et al., 2000).

### In a patient population, expression of AN genes is associated with metabolic and autoimmune phenotypes

Unlike GWAS, which include carefully constructed case–control cohorts, pheWAS encompass all individuals within a healthcare system, including patients with subthreshold or partial presentations of a disorder, or individuals with commonly comorbid or co-diagnosed conditions. Importantly, because individuals are not ascertained for any specific disorder, they represent a more comprehensive clinical picture of the comorbidities and symptomatology associated with AN gene expression.

Examining the consequences of aberrant predicted gene expression among these patients (i.e. testing for GReX associations) may reveal clinical and biological consequences of these genes; for example, studying whether AN-associated genes have anthropometric and metabolic consequences among adults with no evidence for previous AN- or ED-diagnoses may disentangle whether certain endophenotypes present as a cause or consequence of AN. Food avoidance and restriction may arise due to gastrointestinal (GI) complaints and distress that provoke these behaviors and precede development of AN (Zucker & Bulik, 2020). Similarly, autoimmune disorders of the GI tract, such as celiac disease and Crohn's disease, show a bidirectional relationship with AN, with previous diagnosis of a GI-associated autoimmune disorders increasing the risk of AN and vice versa (Hedman et al., 2019). Our pheWAS results of AN-GReX associations with GI symptoms such as abdominal pain, ascites, and peptic ulcer, as well as GI-related autoimmune disorders such as celiac disease, suggest AN-GReX may contribute directly to these diseases and phenotypes, and that food aversive behaviors and gastric distress may be genetically regulated in these individuals, rather than occurring as a consequence of AN. We found a sex-specific association of *CLIC1*-GReX with celiac disease in females (online Supplementary Fig. S3B), indicating sex-specific regulation of AN-genes may be contributing to the disparity in autoimmune diagnoses and symptomatology between the sexes (Fuchs et al., 2014; Ludvigsson & Murray, 2019). Our main pheWAS diagnosis results of celiac disease and type 1 diabetes further show concordance with AN in the direction of effect of the tissue GReX, indicating a similarity in the genetic regulation of AN-genes between patients with these disorders and individuals with AN (online Supplementary Fig. 4A; Supplemental Results). Although these associations could be the consequence of undiagnosed AN-individuals in our biobank, they more likely reflect real biological associations of expression of those particular genes with the phenotype.

Our results further confirm the contribution of metabolic factors to AN etiology; we see very robust association of AN-GReX with type 1 diabetes, the hyperglycemic hormone glucagon and various forms of insulin. The association of AN with metabolic traits and abnormalities has been fairly well established, including with insulin resistance (*r*_g_ = −0.29), fasting insulin (*r*_g_ = −0.24), leptin (*r*_g_ = −0.26) and type 2 diabetes (*r*_g_ = −0.22), and a positive genetic correlation with HDL cholesterol (*r*_g_ = 0.21) (Adams, Reay, Geaghan, & Cairns, 2021; Ilyas et al., 2019; Watson et al., 2019). Notably, our results point to a similar role of aberrant glycemic regulation in the etiology of AN. Future analyses including EHR-derived lab results (LabWAS) studies may further elucidate AN-genes associated with abnormal metabolic regulation and clinical features.

### Context-specific associations reveal a role for BMI in the regulation of AN-gene expression

We stratified our pheWAS analyses by BMI in order to observe whether the effects of predicted AN-gene expression on clinical outcomes were moderated by BMI status. Understanding how BMI influences associations between AN-GReX and the clinical phenome may give us further insight into the biological mechanisms leading to those outcomes and the conferral of AN disease risk. The context-specific associations we see between BMI status and the association of AN-GReX with multiple phenotypes indicate the potential differences in AN-gene regulation in an environment of higher or lower BMI.

Among our BMI-stratified pheWAS analyses, we identified a number of associations between AN-GReX and foot pain, exclusive to the Low-BMI group. We hypothesize that these associations may stem from exercise-related injuries among this group. Multiple genes in our study, many of which were associated with foot pain in Low-BMI individuals, have been previously associated with regular gym attendance (online Supplementary Table S4B, *p*_adj_ = 3.4 × 10^−10^), or measures of physical activity. Excessive and compulsive exercise is a behavior often seen in individuals with AN (Bulik, 2004; Shroff et al., 2006), and evidence is suggestive that hyperactivity increases risk of chronic AN (Achamrah, Coëffier, & Déchelotte, 2016; Strober, Freeman, & Morrell, 1997). Similarly, studies have shown a genetic correlation between AN and physical activity (*r*_g_ = 0.17) (Hübel et al., 2019b; Watson et al., 2019). These genes may reflect a genetic liability to compulsive physical activity. Given the known association of AN disease with low bone mineral density and propensity for bone fracture (Solmi et al., 2016), our results may reflect the result of genes associated with compulsive exercise and bone mineral density contributing to increased osteoarticular pain.

We saw distinct modulation by BMI of *MGMT*-GReX-cholesterol associations in our cohort. Variants in the *MGMT* gene have been shown to be associated with AN (Watson et al., 2019), as well as anthropometric phenotypes such as waist-to-hip ratio and body height (Kichaev et al., 2019). *CTNNB1*, which was associated with both highest and lowest weight measures in our cohort, distinctly with measures of highest weight in High-BMI individuals, has been previously shown to be associated with lean body mass (Hübel et al., 2019a). We saw distinct associations of *PROS1*-GReX with lowest weight in Low-BMI individuals, which is a gene involved in many biological processes, including inflammation (Suleiman, Négrier, & Boukerche, 2013). Our results appear to be due to the effect of BMI on AN-GReX, rather than the direct effect of BMI/weight on the phenotypes (online Supplementary Fig. S4B). Future studies are needed to assess the specific role of BMI on AN-gene regulation.

Although the hypothesis-free, phenome-wide design of our study allows for powerful detection of clinical and biological associations of AN-risk genes, the same design also bears some notable limitations and caveats. One key caveat is the lack of diagnostic detail available for the patients in our study. EHR analyses leverage large, existing data sets to rapidly amass cohorts for analysis, to yield insights into whole phenome consequences of genotype and GReX associations; however, the scale and scope of these studies precludes deep phenotyping or performing clinical interviews. This lack of diagnostic precision may arise from a number of factors.

First, we make use of ICD codes within the medical record in order to infer diagnoses; since these are primarily used by clinicians for billing purposes, they likely provide an imperfect proxy for true disease state. In order to mitigate spurious results stemming from imperfectly assigned codes, we require ‘cases’ to have at least two instances of an ICD code; individuals with only one code were set to missing. Furthermore, for each code we restricted our meta-analysis to include only phenotypes with at least 10 cases and 10 controls, and required a total effective sample size >100 for inclusion in our final analysis.

Second, it is possible that our patients regularly receive treatment at multiple different hospital systems; as such, we may be capturing only partial data for each of our patients. In order to mitigate this, we restricted our analysis to patients with multiple data points within our EHR. Future analyses that seek to harmonize or meta-analyze patient data across EHR (e.g. NYC-CDRN, PsycheMERGE) are ongoing, and will be vital to disentangling this effect further.

Importantly, our patients have not all been explicitly assessed for EDs, and information regarding ED diagnoses and assessments earlier in life may be omitted from the records due to incomplete clinical history assessments. Therefore, it is possible that case-contamination within some of our sample is responsible for the associations observed within our data. In order to address this, we performed a simple experiment to simulate the effect sizes expected if undiagnosed AN contamination drives our result. We estimated the expected association statistics observed due to possible levels of contamination among pheWAS cases. Our model indicates that diagnostic contamination at 0.06, 1.2, or 3% is insufficient to account for the effect sizes observed within our pheWAS analysis. Moreover, since these genes are selected due to their large S-PrediXcan effect sizes, we expect that the contamination effects observed for these two genes will be greater than any others in our study; as such, we do not believe that our findings are attributable to hidden case contamination.

Our results illustrate the clinical outcomes associated with differences in AN-gene expression. Characterization of the phenotypic associations with AN-gene expression in a clinical setting can give us more insight into the biological mechanisms underlying AN and, consequently, how to diagnose and treat the disorder. By understanding the associations of AN-gene expression with symptomatology, prodromal or subthreshold disease states, we may gain insights into the biology of the disease, and perhaps identify therapeutic targets and opportunities for clinical interventions. For example, if GI complaints are truly the consequence of aberrant AN-gene expression, and contribute to disordered eating due to GI distress, treatment of those symptoms may help alleviate other AN symptoms or prevent development of later AN (Riedlinger et al., 2020; Wiklund et al., 2019). An understanding of the clinical associations of AN-gene expression can further augment the definition of AN, and could allow clinicians to more broadly identify individuals at greater risk of AN, or those who present with symptom constellations that do not yet meet the established diagnostic threshold.

## Supplementary material

For supplementary material accompanying this paper visit https://doi.org/10.1017/S0033291721004554. click here to view supplementary material
