# Supplementary material for: Mapping anorexia nervosa genes to clinical phenotypes
Source: Psychol Med. 2022 Apr 5;53(6):2619–33. doi: 10.1017/S0033291721004554 (PMC10123844; doi:10.1017/S0033291721004554)
Supplement: Supplementary file 1 [file S0033291721004554sup.zip › S0033291721004554sup003.docx]

**Eating Disorders Working Group of the Psychiatric Genomics Consortium**

Roger A. H. Adan^1-3^, Lars Alfredsson^4^, Tetsuya Ando^5^, Ole A. Andreassen^6^, Harald Aschauer^7^, Jessica H. Baker^8^, Vladimir Bencko^9^, Andrew W. Bergen^10,11^, Wade H. Berrettini^12^, Andreas Birgegård^13^, Joseph M. Boden^14^, Ilka Boehm^15^, Vesna Boraska Perica^16,17^, Harry Brandt^18^, Gerome Breen^19,20^, Julien Bryois^13^, Cynthia M. Bulik^8,13, 21^, Roland Burghardt^22^, Laura Carlberg^23^, Matteo Cassina^24^, Sven Cichon^25-27^, Maurizio Clementi^24^, Jonathan R. I. Coleman^19,20^, Roger D. Cone^28^, Philippe Courtet^29^, Steven Crawford^18^, Scott Crow^30^, James J. Crowley^31,32^, Unna N. Danner^2^, Oliver S. P. Davis^33-35^, Martina deZwaan^36^, George Dedoussis^37^, Daniela Degortes^38^, Janiece E. DeSocio^39^, Danielle M. Dick^40-42^, Dimitris Dikeos^43^, Christian Dina^44^, Monika Dmitrzak-Weglarz^45^, Elisa Docampo^46-48^, Laramie E. Duncan^49^, Philibert Duriez^50,51^, Karin Egberts^52^, Stefan Ehrlich^15^, Geòrgia Escaramís^46-48^, Tõnu Esko^53,54^, Thomas Espeseth^55,56^, Xavier Estivill^46-48,57^, Anne Farmer^19^, Angela Favaro^38^, Fernando Fernández-Aranda^58,59^, Manfred M. Fichter^60,61^, Krista Fischer^53^, James A. B. Floyd^62^, Manuel Föcker^63^, Lenka Foretova^64^, Andreas J. Forstner^27,65,66^, Monica Forzan^24^, Christopher S. Franklin^16^, Steven Gallinger^67^, Giovanni Gambaro^68^, Héléna A. Gaspar^19,20^, Ina Giegling^69^, Paola Giusti-Rodríquez^31^, Fragiskos Gonidakis^43^, Scott Gordon^70^, Philip Gorwood^50,51^, Monica Gratacos Mayora^46-48^, Jakob Grove^71-74^, Sébastien Guillaume^29^, Yiran Guo^75^, Hakon Hakonarson^75,76^, Katherine A. Halmi^77^, Ken B. Hanscombe^19^, Konstantinos Hatzikotoulas^16,78^, Joanna Hauser^79^, Johannes Hebebrand^80^, Sietske G. Helder^19,81^, Anjali Henders^82^, Stefan Herms^25,26^, Beate Herpertz-Dahlmann^83^, Wolfgang Herzog^84^, Anke Hinney^80^, L. John Horwood^14^, Christopher Hübel^19,85^, Laura M. Huckins^86-89^, James I. Hudson^90^, Hartmut Imgart^91^, Hidetoshi Inoko^92^, Vladimir Janout^93^, Susana Jiménez-Murcia^58,59^, Craig Johnson^94^, Jessica S. Johnson^86,87^, Jennifer Jordan^95,96^, Antonio Julià^97^, Gursharan Kalsi^19^, Deborah Kaminská^98^, Allan S. Kaplan^99-101^, Jaakko Kaprio^102^, Leila Karhunen^103^, Andreas Karwautz^104^, Martien J. H. Kas^1,105^, Walter H. Kaye^106^, James L. Kennedy^99-101^, Martin A. Kennedy^107^, Anna Keski-Rahkonen^108^, Kirsty Kiezebrink^109^, Youl-Ri Kim^110^, Katherine M. Kirk^111^, Lars Klareskog^112^, Kelly L. Klump^113^, Gun Peggy S. Knudsen^114^, Maria C. La Via^8^, Mikael Landén^13,115^, Janne T. Larsen^72,85,116^, Stephanie Le Hellard^117,118^, Virpi M. Leppä^13^, Dong Li^75^, Paul Lichtenstein^13^, Lisa Lilenfeld^119^, Bochao Danae Lin^1^, Jolanta Lissowska^120^, Astri J. Lundervold^121^, Jurjen Luykx^1^, Pierre J. Magistretti^122-123^, Sarah L. Maguire^194^, Mario Maj^124^, Katrin Mannik^53,125^, Sara Marsal^97^, Christian R. Marshall^126^, Nicholas G. Martin^70^, Manuel Mattheisen^32,71,127,128^, Morten Mattingsdal^6^, Sara McDevitt^129,130^, Peter McGuffin^19^, Sarah E. Medland^111^, Andres Metspalu^53,131^, Ingrid Meulenbelt^132^, Nadia Micali^133,134^, James Mitchell^135^, Karen Mitchell^136,137^, Alessio Maria Monteleone^124^, Palmiero Monteleone^138^, Grant W. Montgomery^82,111,139^, Preben Bo Mortensen^72,85,116^, Melissa A. Munn-Chernoff^8^, Benedetta Nacmias^140,141^, Marie Navratilova^64^, Ioanna Ntalla^37^, Catherine M. Olsen^142^, Roel A. Ophoff^143,144^, Julie O’Toole^145^, Leonid Padyukov^112^, Aarno Palotie^54,102,146^, Jacques Pantel^147^, Hana Papezova^98^, Richard Parker^111^, John F. Pearson^148^, Nancy L. Pedersen^13^, Triinu Peters^80^, Liselotte V. Petersen^72,85,116^, Dalila Pinto^87^, Kirstin L. Purves^19^, Anu Raevuori^108,149^, Nicolas Ramoz^51^, Ted Reichborn-Kjennerud^114,150^, Valdo Ricca^151^, Samuli Ripatti^152^, Stephan Ripke^153-155^, Marion Roberts^19^, Alessandro Rotondo^156^, Dan Rujescu^69^, Filip Rybakowski^157^, Paolo Santonastaso^158^, André Scherag^159^, Stephen W. Scherer^160,161^, Ulrike Schmidt^162^, Nicholas J. Schork^163^, Alexandra Schosser^164^, Jochen Seitz^83^, Lenka Slachtova^165^, P. Eline Slagboom^132^, Margarita C. T. Slof-Op’t Landt^166,167^, Agnieszka Slopien^168^, Nicole Soranzo^16,169-171^, Sandro Sorbi^140,141^, Lorraine Southam^16,78^, Vidar W. Steen^172,173^, Michael Strober^174,175^, Garret D. Stuber^8,176^, Patrick F. Sullivan^8,13,31^, Beata Świątkowska^177^, Jin P. Szatkiewicz^31^, Ioanna Tachmazidou^16^, Friederike I. Tam^15,178^, Elena Tenconi^38^, Laura M. Thornton^8^, Alfonso Tortorella^179^, Federica Tozzi^180^, Janet Treasure^19^, Artemis Tsitsika^181^, Marta Tyszkiewicz-Nwafor^168^, Konstantinos Tziouvas^182^, Annemarie van Elburg^2,183^, Eric F. van Furth^166,167^, Tracey D. Wade^184^, Gudrun Wagner^104^, Esther Walton^15^, Hunna J. Watson^8,185,186^, Thomas Werge^187^, David C. Whiteman^142^, H-Erich Wichmann^188^, Elisabeth Widen^102^, D. Blake Woodside^100,101,189,190^, Jiayi Xu^86^, Shuyang Yao^13^, Zeynep Yilmaz^8,13,31,85^, Eleftheria Zeggini^16,78,191^, Stephanie Zerwas^8^, Stephan Zipfel^192,193^.

^1^Brain Center Rudolf Magnus, Department of Translational Neuroscience, University Medical Center Utrecht, Utrecht, The Netherlands.^2^Center for Eating Disorders Rintveld, Altrecht Mental Health Institute, Zeist, The Netherlands.^3^Sahlgrenska Academy, University of Gothenburg, Gothenburg, Sweden.^4^Institute of Environmental Medicine, Karolinska Institutet, Stockholm, Sweden. ^5^Department of Behavioral Medicine, National Institute of Mental Health, National Center of Neurology and Psychiatry, Kodaira, Tokyo, Japan. ^6^NORMENT KG Jebsen Centre, Division of Mental Health and Addiction, University of Oslo, Oslo University Hospital, Oslo, Norway.^7^Biopsychosocial Corporation, Vienna, Austria. ^8^Department of Psychiatry, University of North Carolina at Chapel Hill, Chapel Hill, North Carolina, USA. ^9^First Faculty of Medicine, Institute of Hygiene and Epidemiology, Charles University, Prague, Czech Republic.^10^BioRealm, LLC, Walnut, California, USA. ^11^Oregon Research Institute, Eugene, Oregon, USA.^12^Department of Psychiatry, Center for Neurobiology and Behavior, University of Pennsylvania Perelman School of Medicine, Philadelphia, Pennsylvania, USA.^13^Department of Medical Epidemiology and Biostatistics, Karolinska Institutet, Stockholm, Sweden.^14^Christchurch Health and Development Study, University of Otago, Christchurch, New Zealand.^15^Division of Psychological and Social Medicine and Developmental Neurosciences, Faculty of Medicine, Technische Universität Dresden, Dresden, Germany.^16^Wellcome Sanger Institute, Wellcome Genome Campus, Hinxton, Cambridge, UK.^17^Department of Medical Biology, School of Medicine, University of Split, Split, Croatia.^18^The Center for Eating Disorders at Sheppard Pratt, Baltimore, Maryland, USA.^19^Institute of Psychiatry, Psychology and Neuroscience, Social, Genetic and Developmental Psychiatry (SGDP) Centre, King’s College London, London, UK.^20^National Institute for Health Research Biomedical Research Centre, King’s College London and South London and Maudsley National Health Service Trust, London, UK.^21^Department of Nutrition, University of North Carolina at Chapel Hill, Chapel Hill, North Carolina, USA.^22^Klinikum Frankfurt/Oder, Frankfurt, Germany.^23^Medical University of Vienna, Vienna, Austria.^24^Clinical Genetics Unit, Department of Woman and Child Health, University of Padova, Padova, Italy.^25^Institute of Medical Genetics and Pathology, University Hospital Basel, Basel, Switzerland.^26^Department of Biomedicine, University of Basel, Basel, Switzerland.^27^Institute of Neuroscience and Medicine (INM-1), Research Center Juelich, Juelich, Germany.^28^Life Sciences Institute and Department of Molecular and Integrative Physiology, University of Michigan, Ann Arbor, Michigan, USA.^29^Department of Emergency Psychiatry and Post-Acute Care, CHRU Montpellier, University of Montpellier, Montpellier, France.^30^Department of Psychiatry, University of Minnesota, Minneapolis, Minnesota, USA.^31^Department of Genetics, University of North Carolina at Chapel Hill, Chapel Hill, North Carolina, USA.^32^Department of Clinical Neuroscience, Karolinska Institutet, Stockholm, Sweden.^33^MRC Integrative Epidemiology Unit, University of Bristol, Bristol, UK.^34^Bristol Medical School, University of Bristol, Bristol, UK.^35^The Alan Turing Institute, London, UK.^36^Department of Psychosomatic Medicine and Psychotherapy, Hannover Medical School, Hannover, Germany.^37^Department of Nutrition and Dietetics, Harokopio University, Athens, Greece.^38^Department of Neurosciences, University of Padova, Padova, Italy.^39^College of Nursing, Seattle University, Seattle, Washington, USA.^40^Department of Psychology, Virginia Commonwealth University, Richmond, Virginia, USA.^41^College Behavioral and Emotional Health Institute, Virginia Commonwealth University, Richmond, Virginia, USA.^42^Department of Human and Molecular Genetics, Virginia Commonwealth University, Richmond, Virginia, USA.^43^First Department of Psychiatry, National and Kapodistrian University of Athens, Medical School, Eginition Hospital, Athens, Greece.^44^L’institut du thorax, INSERM, CNRS, UNIV Nantes, Nantes, France.^45^Department of Psychiatric Genetics, Poznan University of Medical Sciences, Poznan, Poland.^46^Barcelona Institute of Science and Technology, Barcelona, Spain.^47^Universitat Pompeu Fabra, Barcelona, Spain.^48^Centro de Investigación Biomédica en Red en Epidemiología y Salud Pública (CIBERESP), Barcelona, Spain.^49^Department of Psychiatry and Behavioral Sciences, Stanford University, Stanford, California, USA.^50^GHU Paris Psychiatrie et Neurosciences, CMME, Paris Descartes University, Paris, France.^51^INSERM U1266, Institute of Psychiatry and Neurosciences, Paris, France.^52^Department of Child and Adolescent Psychiatry, Psychosomatics and Psychotherapy, University Hospital of Würzburg, Centre for Mental Health, Würzburg, Germany.^53^Estonian Genome Center, University of Tartu, Tartu, Estonia.^54^Program in Medical and Population Genetics, Broad Institute of Massachusetts Institute of Technology and Harvard University, Cambridge, Massachusetts, USA.^55^Department of Psychology, University of Oslo, Oslo, Norway.^56^Bjørknes College, Oslo, Norway.^57^Genomics and Disease, Bioinformatics and Genomics Programme, Centre for Genomic Regulation, Barcelona, Spain.^58^Department of Psychiatry, University Hospital of Bellvitge – IDIBELL and CIBERobn, Barcelona, Spain.^59^Department of Clinical Sciences, School of Medicine, University of Barcelona, Barcelona, Spain.^60^Department of Psychiatry and Psychotherapy, Ludwig-Maximilians-University (LMU), Munich, Germany.^61^Schön Klinik Roseneck affiliated with the Medical Faculty of the University of Munich, Munich, Germany.^62^Genomics plc, Genomics PLC, Oxford, UK.^63^Department of Child and Adolescent Psychiatry, University of Münster, Münster, Germany.^64^Department of Cancer, Epidemiology and Genetics, Masaryk Memorial Cancer Institute, Brno, Czech Republic.^65^Institute of Human Genetics, University of Bonn, School of Medicine and University Hospital Bonn, Bonn, Germany.^66^Centre for Human Genetics, University of Marburg, Marburg, Germany.^67^Department of Surgery, Faculty of Medicine, University of Toronto, Toronto, Ontario, Canada.^68^Division of Nephrology and Dialysis, Department of Medicine, AOVR, Ospedale Maggiore, Verona, Italy.^69^Department of Psychiatry, Psychotherapy and Psychosomatics, Martin Luther University of Halle-Wittenberg, Halle (Saale), Germany.^70^Genetics and Computational Biology Department, QIMR Berghofer Medical Research Institute, Brisbane, Queensland, Australia.^71^Department of Biomedicine, Aarhus University, Aarhus, Denmark.^72^The Lundbeck Foundation Initiative for Integrative Psychiatric Research (iPSYCH), Aarhus, Denmark.^73^Centre for Integrative Sequencing, iSEQ, Aarhus University, Aarhus, Denmark.^74^Bioinformatics Research Centre, Aarhus University, Aarhus, Denmark.^75^Center for Applied Genomics, Children’s Hospital of Philadelphia, Philadelphia, Pennsylvania, USA.^76^Department of Pediatrics, University of Pennsylvania Perelman School of Medicine, Philadelphia, Pennsylvania, USA.^77^Department of Psychiatry, Weill Cornell Medical College, New York, New York, USA.^78^Institute of Translational Genomics, Helmholtz Zentrum München – German Research Centre for Environmental Health, Neuherberg, Germany.^79^Department of Adult Psychiatry, Poznan University of Medical Sciences, Poznan, Poland.^80^Department of Child and Adolescent Psychiatry, University Hospital Essen, University of Duisburg-Essen, Essen, Germany.^81^Zorg op Orde, Delft, The Netherlands.^82^Institute for Molecular Bioscience, University of Queensland, Brisbane, Queensland, Australia.^83^Department of Child and Adolescent Psychiatry, Psychosomatics and Psychotherapy, RWTH Aachen University, Aachen, Germany.^84^Department of General Internal Medicine and Psychosomatics, Heidelberg University Hospital, Heidelberg University, Heidelberg, Germany.^85^National Centre for Register-Based Research, Aarhus BSS, Aarhus University, Aarhus, Denmark.^86^Pamela Sklar Division of Psychiatric Genomics, Icahn School of Medicine at Mount Sinai, New York, New York, USA.^87^Department of Psychiatry, and Genetics and Genomic Sciences, Icahn School of Medicine at Mount Sinai, New York, New York, USA.^88^Seaver Autism Center for Research and Treatment, Icahn School of Medicine at Mount Sinai, New York, New York, USA.^89^Mental Illness Research, Education and Clinical Centers, James J. Peters Department of Veterans Affairs Medical Center, Bronx, New York, USA.^90^Biological Psychiatry Laboratory, McLean Hospital/Harvard Medical School, Boston, Massachusetts, USA.^91^Eating Disorders Unit, Parklandklinik, Bad Wildungen, Germany.^92^Department of Molecular Life Science, Division of Basic Medical Science and Molecular Medicine, School of Medicine, Tokai University, Isehara, Japan.^93^Faculty of Health Sciences, Palacky University, Olomouc, Czech Republic.^94^Eating Recovery Center, Denver, Colorado, USA.^95^Department of Psychological Medicine, University of Otago, Christchurch, New Zealand.^96^Canterbury District Health Board, Christchurch, New Zealand.^97^Rheumatology Research Group, Vall d’Hebron Research Institute, Barcelona, Spain.^98^First Faculty of Medicine, Department of Psychiatry, Charles University, Prague, Czech Republic.^99^Centre for Addiction and Mental Health, Toronto, Ontario, Canada.^100^Institute of Medical Science, University of Toronto, Toronto, Ontario, Canada.^101^Department of Psychiatry, University of Toronto, Toronto, Ontario, Canada.^102^Institute for Molecular Medicine Finland FIMM, HiLIFE, Helsinki Institute of Life Science, University of Helsinki, Helsinki, Finland.^103^Institute of Public Health and Clinical Nutrition, Department of Clinical Nutrition, University of Eastern Finland, Kuopio, Finland.^104^Eating Disorders Unit, Department of Child and Adolescent Psychiatry, Medical University of Vienna, Vienna, Austria.^105^Groningen Institute for Evolutionary Life Sciences, University of Groningen, Groningen, The Netherlands.^106^Department of Psychiatry, University of California San Diego, San Diego, California, USA.^107^Department of Pathology and Biomedical Science, University of Otago, Christchurch, New Zealand.^108^Department of Public Health, University of Helsinki, Helsinki, Finland.^109^Institute of Applied Health Sciences, School of Medicine, Medical Sciences and Nutrition, University of Aberdeen, Aberdeen, UK.^110^Department of Psychiatry, Seoul Paik Hospital, Inje University, Seoul, Korea.^111^QIMR Berghofer Medical Research Institute, Brisbane, Queensland, Australia.^112^Division of Rheumatology, Department of Medicine, Center for Molecular Medicine, Karolinska Institutet and Karolinska University Hospital, Stockholm, Sweden.^113^Department of Psychology, Michigan State University, East Lansing, Michigan, USA.^114^Department of Mental Disorders, Norwegian Institute of Public Health, Oslo, Norway.^115^Institute of Neuroscience and Physiology, The Sahlgrenska Academy at the University of Gothenburg, Gothenburg, Sweden.^116^Centre for Integrated Register-based Research (CIRRAU), Aarhus University, Aarhus, Denmark.^117^Dr. Einar Martens Research Group for Biological Psychiatry, Center for Medical Genetics and Molecular Medicine, Haukeland University Hospital, Bergen, Norway.^118^Department of Clinical Medicine, Laboratory Building, Haukeland University Hospital, Bergen, Norway.^119^The Chicago School of Professional Psychology, Washington D.C., USA.^120^Department of Cancer Epidemiology and Prevention, M. Sklodowska-Curie National Research Institute of Oncology, Warsaw, Poland.^121^Department of Biological and Medical Psychology, University of Bergen, Bergen, Norway.^122^BESE Division, KAUST, KSA, King Abdullah University of Science and Technology, Thuwal, Saudi Arabia.^123^Department of Psychiatry, University of Lausanne-University Hospital of Lausanne (UNIL-CHUV), Lausanne, Switzerland.^124^Department of Psychiatry, University of Campania “Luigi Vanvitelli”, Naples, Italy.^125^Center for Integrative Genomics, University of Lausanne, Lausanne, Switzerland.^126^Department of Paediatric Laboratory Medicine, Division of Genome Diagnostics, The Hospital for Sick Children, Toronto, Ontario, Canada.^127^Center for Psychiatry Research, Stockholm Health Care Services, Stockholm City Council, Stockholm, Sweden.^128^Department of Psychiatry, Psychosomatics and Psychotherapy, University of Würzburg, Würzburg, Germany.^129^Department of Psychiatry, University College Cork, Cork, Ireland.^130^Child and Adolescent Regional Eating Disorder Service (CAREDS), Health Service Executive South, Cork, Ireland.^131^Institute of Molecular and Cell Biology, University of Tartu, Tartu, Estonia.^132^Molecular Epidemiology Section, Department of Biomedical Datasciences, Leiden University Medical Centre, Leiden, The Netherlands.^133^Department of Psychiatry, Faculty of Medicine, University of Geneva, Geneva, Switzerland.^134^Department of Pediatrics, Gynaecology and Obstetrics, University of Geneva, Geneva, Switzerland.^135^Department of Psychiatry and Behavioral Science, University of North Dakota School of Medicine and Health Sciences, Fargo, North Dakota, USA.^136^National Center for PTSD, VA Boston Healthcare System, Boston, Massachusetts, USA.^137^Department of Psychiatry, Boston University School of Medicine, Boston, Massachusetts, USA.^138^Department of Medicine, Surgery and Dentistry “Scuola Medica Salernitana”, University of Salerno, Salerno, Italy.^139^Queensland Brain Institute, University of Queensland, Brisbane, Queensland, Australia.^140^Department of Neuroscience, Psychology, Drug Research and Child Health (NEUROFARBA), University of Florence, Florence, Italy.^141^IRCCS Fondazione Don Carlo Gnocchi, Florence, Italy.^142^Population Health Department, QIMR Berghofer Medical Research Institute, Brisbane, Queensland, Australia.^143^Center for Neurobehavioral Genetics, Semel Institute for Neuroscience and Human Behavior, University of California Los Angeles, Los Angeles, California, USA.^144^Department of Psychiatry, Erasmus MC, University Medical Center Rotterdam, Rotterdam, The Netherlands.^145^Kartini Clinic, Portland, Oregon, USA.^146^Center for Human Genome Research, Massachusetts General Hospital, Boston, Massachusetts, USA.^147^INSERM U1124, Université de Paris, Paris, France.^148^Biostatistics and Computational Biology Unit, University of Otago, Christchurch, New Zealand.^149^Department of Adolescent Psychiatry, Helsinki University Hospital, Helsinki, Finland.^150^Institute of Clinical Medicine, University of Oslo, Oslo, Norway.^151^Department of Health Science, University of Florence, Florence, Italy.^152^Department of Biometry, University of Helsinki, Helsinki, Finland.^153^Analytic and Translational Genetics Unit, Department of Medicine, Massachusetts General Hospital and Harvard Medical School, Boston, Massachusetts, USA.^154^Stanley Center for Psychiatric Research, Broad Institute of the Massachusetts Institute of Technology and Harvard University, Cambridge, Massachusetts, USA.^155^Department of Psychiatry and Psychotherapy, Charité – Universitätsmedizin, Berlin, Germany.^156^Department of Psychiatry, Neurobiology, Pharmacology, and Biotechnologies, University of Pisa, Pisa, Italy.^157^Department of Psychiatry, Poznan University of Medical Sciences, Poznan, Poland.^158^Department of Neurosciences, Padua Neuroscience Center, University of Padova, Padova, Italy.^159^Institute of Medical Statistics, Computer and Data Sciences, Jena University Hospital, Jena, Germany.^160^Department of Genetics and Genomic Biology, The Hospital for Sick Children, Toronto, Ontario, Canada.^161^McLaughlin Centre, University of Toronto, Toronto, Ontario, Canada.^162^Institute of Psychiatry, Psychology and Neuroscience, Psychological Medicine, King’s College London, London, UK.^163^J. Craig Venter Institute (JCVI), La Jolla, California, USA.^164^Department of Psychiatry and Psychotherapy, Medical University of Vienna, Vienna, Austria.^165^First Faculty of Medicine, Department of Biology and Medical Genetics, Charles University, Prague, Czech Republic.^166^Center for Eating Disorders Ursula, Rivierduinen, Leiden, The Netherlands.^167^Department of Psychiatry, Leiden University Medical Centre, Leiden, The Netherlands.^168^Department of Child and Adolescent Psychiatry, Poznan University of Medical Sciences, Poznan, Poland.^169^Donor Health and Genomics, National Institute for Health Research Blood and Transplant Unit, Cambridge, UK.^170^Division of Cardiovascular Medicine, British Heart Foundation Centre of Excellence, Cambridge, UK.^171^Department of Haematology, University of Cambridge, Cambridge, UK.^172^Center for Medical Genetics and Molecular Medicine, Haukeland University Hospital, Bergen, Norway.^173^Department of Clinical Science, University of Bergen, Bergen, Norway.^174^Department of Psychiatry and Biobehavioral Science, Semel Institute for Neuroscience and Human Behavior, University of California Los Angeles, Los Angeles, California, USA.^175^David Geffen School of Medicine, University of California Los Angeles, Los Angeles, California, USA.^176^Department of Cell Biology and Physiology, University of North Carolina at Chapel Hill, Chapel Hill, North Carolina, USA.^177^Department of Environmental Epidemiology, Nofer Institute of Occupational Medicine, Lodz, Poland.^178^Eating Disorders Research and Treatment Center, Department of Child and Adolescent Psychiatry, Faculty of Medicine, Technische Universität Dresden, Dresden, Germany.^179^Department of Psychiatry, University of Perugia, Perugia, Italy.^180^Brain Sciences Department, Stremble Ventures, Limassol, Cyprus.^181^Adolescent Health Unit, Second Department of Pediatrics, “P. & A. Kyriakou” Children’s Hospital, University of Athens, Athens, Greece.^182^Pediatric Intensive Care Unit, “P. & A. Kyriakou” Children’s Hospital, University of Athens, Athens, Greece.^183^Faculty of Social and Behavioral Sciences, Utrecht University, Utrecht, The Netherlands.^184^School of Psychology, Flinders University, Adelaide, South Australia, Australia.^185^School of Psychology, Curtin University, Perth, Western Australia, Australia.^186^School of Paediatrics and Child Health, University of Western Australia, Perth, Western Australia, Australia.^187^Department of Clinical Medicine, University of Copenhagen, Copenhagen, Denmark.^188^Helmholtz Centre Munich – German Research Center for Environmental Health, Munich, Germany.^189^Centre for Mental Health, University Health Network, Toronto, Ontario, Canada.^190^Program for Eating Disorders, University Health Network, Toronto, Ontario, Canada.^191^Technical University of Munich (TUM) and Klinikum Rechts der Isar, TUM School of Medicine, Munich, Germany.^192^Department of Internal Medicine VI, Psychosomatic Medicine and Psychotherapy, University Medical Hospital Tuebingen, Tuebingen, Germany.^193^Centre of Excellence for Eating Disorders (KOMET), University Tuebingen, Tuebingen, Germany. ^194^School of Medicine, InsideOut Institute, Sydney, New South Wales, Australia.

**SUPPLEMENTAL METHODS**

***Bio*Me™ *Ancestry QC***

Ancestry was initially assigned to each Bio*Me*™ individual according to six self-reported group designations: African (AA), European (EA), Hispanic (HA), East Asian (ESA), Native American (NA), and O (Other), and confirmed using principal components analysis (PCA). We assigned un-classified and self-declared “Other” individuals, we merged our data with 1000Genomes phase 3 genotypes, and assigned individuals to super-populations using a PCA in PLINK (Chang et al., 2015; Purcell et al., 2007). From this, we assigned individuals to one of six groups: African (AA), European (EA), East Asian (ESA), Hispanic (HA), Native American (NA), and South Asian (SAS). A total of 119 unassigned samples were removed, leaving 31585 individuals for analysis

***BMI Stratification***

We stratified our cohort into three categories of BMI: High, Mid, and Low. In order to determine which individuals fell into each group, we looked at the distribution of BMI for each ancestry group and sex (**Figure S1**). Within each distribution, we decided to use the quartiles of the normal distribution as our cut-offs for assigning BMI group (**Figure S1A**). Individuals whose BMI fell above the 3^rd^ quartile of the BMI distribution were assigned to the High BMI category, those who fell below the 1^st^ quartile were assigned to Low BMI, and those whose BMI fell between the 1^st^ and 3^rd^ quartile of the distribution were assigned to the Mid BMI group. **Table S2** describes the BMI values for each ancestry and sex, as well as the range of BMI values included in each group for each ancestry and sex.

***Bio*Me *BRSPD File QC***

All of the Bio*Me*-R Structured Phenotype Database (BRSPD) files underwent a fairly extensive QC process to correct misspelled variable fields, remove extraneous symbols, standardize units of measure, and replace spaces with underscores (“_”). All of the files were originally delimited by the “|” symbol, and thus were converted to a tab-delimited format for pheWAS use. Many files had variable fields with variations on the same phenotype or measure (for example 1mg/mL could be written as “1_mg/mL”, “1mg/mL”, “1mg1mL”, “1mg/1mL”, etc.). These measures and phenotypes needed to be collapsed and standardized before input into pheWAS. Likewise, there were a few phenotypes, specifically the vital sign measurements, where each individual had many measurements (mean number of measurements = 24), with each measurement representing an encounter with the healthcare system. In order to look at the overall association with these phenotypes, continuous vital sign measures with multiple entries were collapsed into four measures: highest measure recorded, lowest measure recorded, mean measure, and variance of measure. Phenotypes with improbable measures (for example adults with a lowest weight of 10kg or height=100 inches) were removed before collapsing for each individual. For example, if an individual had five measures of height recorded as 65, 65.5, 65, 65, and 100 inches, we would remove the 100-inch measure and then calculate the mean with the remaining values.

*Allergies*

Known allergies and notes on allergic reaction were entered in the original Allergy file, which included individual ID, allergen, date of entry, and notes on the allergic reaction (if available). Most QC of the allergy file entailed collapsing variables of the same or similar phenotype. For example, allergy to tree nuts could be noted as “TREE NUT”, “TREE NUTS”, “TREE NUT UNSPECIFIED”, and “NUT, TREE”. All of these phenotypes were collapsed into the category of “TREE NUT”. After QC of the Allergy file and application of selection criteria for sample size and phenotype counts, we had a total of 113 Allergy phenotypes available for pheWAS. The within-gene tissue-specific Bonferroni p-value threshold was set at 0.05/113 phenotypes (p=4.4 x 10^-4^), and the experiment-wide threshold set at 0.05/(113 phenotypes * 45 tissues) (p=9.8 x 10^-6^).

*Diagnosis codes*

Diagnosis code phenotypes encompassed both the encounter diagnosis and phecode files. The encounter diagnosis file comprises the primary diagnoses, denoted by the International Classification of Disease (ICD) 9 and 10 codes, assigned to each patient by the clinician in their visits and interactions with the health system. Most diagnosis codes in the encounter diagnosis file are ICD-10 codes, however, there were a few diagnoses that were still using ICD-9 codes that had not been converted. We converted the original encounter diagnosis file, which consisted of information on patient ID, diagnosis code, date of entry, notes, and code description, into counts of each diagnosis code per individual. The PheWAS R package *createPhewasTable* function uses this count information to select cases of a particular diagnosis to have a count of 2 or more diagnosis codes, select controls with counts of 0 and exclude individuals with only 1 count of a particular code. Phecodes were assigned from the encounter diagnosis file by grouping ICD-9 and ICD-10 diagnostic codes based on previous phecode mappings (Wu et al., 2019). We defined cases as individuals with at least two counts of a code (“TRUE”). Those with zero counts were considered “controls” (“FALSE”), and those with only one count were set to missing (“NA”). After QC, our dataset included 2178 unique Encounter Diagnosis codes and 1093 unique phecodes. Due to the high correlation between Encounter Diagnosis and phecode files, we combined all results from both PheWAS and performed an FDR correction in *R* to determine significance (p_FDR_<0.05).

*Encounter Orders*

The encounter orders file describes the various lab tests and other procedures ordered by the clinician upon the individual’s encounter with the healthcare system. Similar to the encounter diagnosis file, the encounter orders file includes individual ID, the encounter order procedure code, date of encounter, any notes from the clinician, and notes on whether the order was complete or currently being carried out. For this phenotype, each individual was noted as “TRUE” or “FALSE” as to whether they were assigned a particular order. After QC, there were a total of 1609 phenotypes available for pheWAS. The within-gene tissue-specific Bonferroni p-value threshold was set at 0.05/1609 phenotypes (p=3.1 x 10^-5^), and the experiment-wide threshold set at 0.05/(1609 phenotypes * 45 tissues) (p=6.9 x 10^-7^).

*Family History*

Family history of disease phenotypes were assigned with the family relation who had disorder such as mother, father, sister, brother, paternal grandmother, maternal grandmother, etc. We collapsed each phenotype based on the relationship degree in order to reduce the number of tests. Relationships of mother, father, sister, brother, son, daughter, and child were categorized as “first degree”. Grandchild, half-brother, half-sister, maternal aunt, maternal grandfather, maternal grandmother, maternal uncle, paternal aunt, paternal grandfather, paternal grandmother, and paternal uncle were categorized as “second degree”. Cousin, maternal great aunt, maternal great grandfather, maternal great grandmother, maternal great uncle, paternal great aunt, paternal great grandfather, paternal great grandmother, and paternal great uncle were categorized as “third degree”. Foster, spouse, stepbrother, stepfather and stepsister were categorized as “no relation”. After QC, there were a total of 144 phenotypes for pheWAS.. The within-gene tissue-specific Bonferroni p-value threshold was set at 0.05/144 phenotypes (p=3.5 x 10^-4^), and the experiment-wide threshold set at 0.05/(144 phenotypes * 45 tissues) (p=7.7 x 10^-6^).

*Lipid measures (Total cholesterol, HDL cholesterol, and LDL cholesterol*

Measures of cholesterol, including HDL, LDL and total cholesterol measurements (mg/dL), were derived from lab results from the Bio*Me*™ EHR. Outliers were removed as described in the initial BRSPD QC paragraph above, and three phenotypes derived from each lab measure were used for pheWAS analyses: Highest recorded, lowest recorded, and mean total cholesterol, HDL, and LDL cholesterol levels. The within-gene tissue-specific Bonferroni p-value threshold was set at 0.05/9 phenotypes (p=0.0056), and the experiment-wide threshold set at 0.05/(9 phenotypes * 45 tissues) (p=1.2 x 10^-4^).

*Medications*

We used CLAMP, a clinical natural language processing algorithm, to identify the core ingredients of unique Bio*Me*™ prescriptions from the EHR-derived medications file. Using the unstructured Bio*Me*™ prescriptions as input, CLAMP returns an RxNorm concept unique identifier (RXCUI) for any prescription that contains a suspected drug name. After manual review of 100 random RXCUIs output by CLAMP, each RXCUI from CLAMP was then reintroduced to the RxNorm API to standardize the drugs by their ingredients. From this we were able to collapse and standardize medication names for PheWAS based on their common drug name. For example, sertraline, the generic name for the brand-name SSRI Zoloft, would encompass prescriptions for both the generic name “sertraline” as well as “Zoloft”. After QC a total of 951 medication phenotypes were available for pheWAS. The within-gene tissue-specific Bonferroni p-value threshold was set at 0.05/951 phenotypes (p=5.3 x 10^-5^), and the experiment-wide threshold set at 0.05/(951 phenotypes * 45 tissues) (p=1.2 x 10^-6^).

*OBGYN History*

Obstetric history was derived from a file that included individual, type of pregnancy and/or parity (term, preterm, ectopic, abortion, therapeutic abortion, spontaneous abortion, para, and gravida), and date of OBGYN encounter. The within-gene tissue-specific Bonferroni p-value threshold was set at 0.05/8 phenotypes (p=0.0063), and the experiment-wide threshold set at 0.05/(8 phenotypes * 45 tissues) (p=1.3 x 10^-4^).

*Pain Score and Location*

For pain score and location, we created measurements that we hoped captured both the severity of pain recorded (pain score is a 0 to 10 scale, with 0 as no pain and 10 being the most severe pain ever experienced), as well as the number of times an individual reported a particular pain score. We created pain score measurements that included highest recorded (with a maximum value of 10), mean pain score, and pain score sum, which was the sum of all recorded pain score measurements. In addition to pain score, we also had pain location recorded with pain score. We further created pain score by location, where each location was assessed for highest, mean, and sum pain scores. We looked at pain location overall, regardless of score, as an additional phenotype. The within-gene tissue-specific Bonferroni p-value threshold was set at 0.05/99 phenotypes (p=5.1 x 10^-4^), and the experiment-wide threshold set at 0.05/(99 phenotypes * 45 tissues) (p=1.1 x 10^-5^).

*Personal History*

Personal history phenotypes were extracted from a large 156-question questionnaire that had information on demographics, social history, education, behavior, personal medical history, and more. After QC, a total of 35 phenotypes were available for pheWAS. The within-gene tissue-specific Bonferroni p-value threshold was set at 0.05/35 phenotypes (p=0.0014), and the experiment-wide threshold set at 0.05/(35 phenotypes * 45 tissues) (p=3.2 x 10^-5^).

*Social History (Alcohol, Illicit Drug and Tobacco use; Sexual History)*

Social history variables containing information on education, tobacco and alcohol use, illicit drug use, and sexual history were available within the BRSPD files. We split this file into multiple phenotype files encompassing categorical and continuous measures of tobacco, alcohol and illicit drug use, as well as sexual history. Continuous measures of alcohol use were measured as ounces of alcohol consumed per week, illicit drug use was measured as frequency or number of times used, and tobacco use was separated into two continuous measures of number of cigarette packs smoked per day and number of years of tobacco use. Categorical measures of alcohol, illicit drug and tobacco use included Y/N questions on use and exposure (yes/no) and type of user (current, past, never). Sexual history consisted of categorical measures of sexual activity and contraceptive use. The within-gene tissue-specific Bonferroni p-value threshold was set at 0.05/31 phenotypes (p=0.0016), and the experiment-wide threshold set at 0.05/(31 phenotypes * 45 tissues) (p=3.6 x 10^-5^).

*Vital Signs (Height, Weight, Blood Pressure, Temperature, Respirations, Pulse, Pulse Oximetry))*

The original vital signs file included information on Individual ID, type of vital sign, vital sign measure, unit of measure, and date of measurement for nine signs: blood pressure, height, pulse, pulse oximetry, respirations, temperature, and weight. Multiple measures were made for each individual’s encounter with the Mount Sinai health system. To account for these multiple measures we first removed any measurements that were clear, biologically impossible outliers (e.g. an individual who had a height measurement of 1,200 inches), then standardized all measurements to the same unit (e.g. cm for height, kg for weight and degrees Celsius for temperature), and finally summarized each individual’s measurements into four measures per phenotype: highest recorded measurement, lowest recorded measurement, mean measurement and variance. Blood pressure measurements were given as separate systolic and diastolic pressure measurements, and we also included a measure of pulse pressure, which was calculated as the difference between systolic and diastolic measurements.

For the weight phenotype, we chose to use three measurements: highest recorded weight, lowest recorded weight, and weight change over time, which was calculated by using the earliest and most recent recorded weight measures divided by the number of years between those records as the average number of kg change per year. This obviously will not reflect weight cycling and other trajectories over time, but gives us a rough mean estimate of weight fluctuation over time. Future analyses will include more precise weight trajectories for our pheWAS. The within-gene tissue-specific Bonferroni p-value threshold for vital signs was set at 0.05/32 phenotypes (p=0.0016), and the experiment-wide threshold set at 0.05/(32 phenotypes * 45 tissues) (p=3.5 x 10^-5^).

**Testing for hidden case contamination**

Consider a binary trait, D_x_, which occurs in *N_+_* cases within Bio*Me*™. If there is no association between GReX and D_x_, we expect average GReX to be equal among cases and controls; essentially, we observe the same difference in gene expression as if we had randomly sampled any two groups of individuals.

If there is an association between G and D_x_ that is driven by unidentified case contamination (that is, by individuals within our case group that are incorrectly missing information about AN diagnoses) then we can estimate the expect effect size due to this contamination.

First, we assume that all other individuals, with or without the trait D_x,_ have GReX with mean G and standard deviation $g_{var}$.

Next, we assume all GReX differences are driven by contamination, which occurs at prevalence *p* and with effect size $\beta$ on gene expression. Assuming a well-designed and adequately powered GWAS and S-PrediXcan study, as here, we may estimate $\beta$ directly from S-PrediXcan summary statistics.

**Among controls:**

We estimate average GReX as the sum of $\left( G \pm g_{var} \right)$ for all $N_{-}$ individuals not expressing trait D_x_, as follows:

$$\bar{{GReX}_{-}}=G= \frac{\sum_{i=1}^{N_{-}} \left( G \pm g_{var} \right)}{N_{-}}$$

Similarly, it is trivial to derive the expected variance of this distribution:

$$\sigma_{-}^{2}= {g_{var}}^{2}=\frac{\sum_{i=1}^{N_{-}} {\left( (G \pm g_{var} \right)-\bar{x})}^{2}}{N_{-}}$$

**Among cases**:

For the subset of individuals ($N_{AN})$ with undiagnosed or undeclared AN, GReX will be up- or downregulated by S-PrediXcan effect size $\beta$;

For all other individuals ($N_{AN}+1: N_{+}$), GReX follows the previously defined normal distribution:

$$\bar{{GReX}_{+}}= \frac{N_{AN}(\beta)G +G(N_{+}-N_{AN})}{N_{+}}$$

To simplify: $p$ is the proportion of diagnosis-contaminated cases, such that $pN_{+}=N_{AN}$

$$=\frac{\left( G \right)\left( pN_{+}\beta\right)+G\left( N_{+}-pN_{+} \right)}{N_{+}}$$

$$=\frac{pN_{+}\left( \beta\right)G+G(N_{+})(1-p)}{N_{+}}$$

$$= p\left( \beta\right)G+G(1-p)$$

Similarly, we may derive the expected variance among cases as follows:

$$\sigma_{+}^{2}=\frac{N_{AN}\sigma_{AN}^{2}+\left( N_{+}-N_{AN} \right){g_{var}}^{2}+N_{AN}\left( \beta G-\bar{x} \right)^{2}+\left( N_{+}-N_{AN} \right)\left( G-\bar{x} \right)^{2}}{N_{+}}$$

To calculate the expected variance among individuals with undeclared AN:

$$\sigma_{AN}^{2}=\frac{1}{N}\sum_{i=1}^{N} ({\beta\chi_{i}-\beta G)}^{2}$$

$$=\frac{1}{N}\sum_{i=1}^{N} {\beta(\chi_{i}-G)}^{2}$$

$$=(\beta^{2})\frac{1}{N}\sum_{i=1}^{N} {(\chi_{i}-G)}^{2}$$

$$={{(\beta^{2})g}_{var}}^{2}$$

To simplify the expected case variance: we consider that $pN_{+}=N_{AN}$, $\bar{x}=\bar{{GReX}_{+}}$, and $\sigma_{AN}^{2}={{(\beta^{2})g}_{var}}^{2}$

$$\sigma_{+}^{2}=\frac{{pN}_{+}{{(\beta^{2})g}_{var}}^{2}+\left( N_{+}-{pN}_{+} \right){g_{var}}^{2}+{pN}_{+}{(\beta G-\bar{{GReX}_{+}})}^{2}+(N_{+}-pN_{+}){(G-\bar{{GReX}_{+}})}^{2}}{N_{+}}$$

$$=p(\beta^{2})\left( {g_{var}}^{2} \right)+\left( 1-p \right){g_{var}}^{2}+p{(BG-\bar{{GReX}_{+}})}^{2}+(1-p){(G-\bar{{GReX}_{+}})}^{2}$$

**Comparing cases and controls**

The **expected difference** **δ** between D_x_ cases and controls (δ) may be calculated as follows:

$$=\bar{{GReX}_{+}}-\bar{{GReX}_{-}}= p\left( \beta\right)G+G\left( 1-p \right)-G$$

$$=G(p\beta+1-p-1)$$

$$=pG(\beta-1)$$

Finally, the statistical significance of the difference between the two distributions (x,y) may be estimated from first principles using a T-Score, as follows:

$$T=\frac{\bar{{GReX}_{+}}-\bar{{GReX}_{-}}}{\sqrt{\frac{\sigma_{+}^{2}}{N_{+}}+\frac{\sigma_{-}^{2}}{N_{-}}}}$$

Where individual terms are as calculated above.

In order to test this hypothesis, we simulated gene expression following a normal distribution with mean G=2 and standard deviation $g_{var}$=0.1 for (i) 1,000 cases and 1,000 controls; (ii) 1,000 cases and 10,000 controls; (iii) 1,000 cases and 30,000 controls.

We introduced diagnostic contamination into our case group (x) at seven different rates (p=0.1, 0.5, 0.05, 0.02, 0.01, 0.005, 0.001), at nine different effect levels ($\beta$ =1,2,3,4,5,10,0.1, 0.25, 0.5). Following the formulae above, samples within our case group (x) had expression levels $\beta\left( G \pm g_{var} \right)$.

For each p x$\beta$combination we performed 10,000 permutations, and calculated mean expression levels within and across distributions; expected variance within and across distributions; and T-scores and p-values of expected significance of the difference between distributions.

Across all p x$\beta$combinations and case/control proportions, the formulae derived above accurately estimate the observed values (**Figure S3; Supplemental Material**).

**SUPPLEMENTAL RESULTS**

***S-PrediXcan of AN***

We used a binomial test to look for tissue enrichment within our experiment-wide significant gene-tissue associations and found no significant enrichment of any tissue. We then looked at tissue enrichment across nominally significant gene-tissue associations (p<0.05) and found significant enrichment of whole blood tissue (p=5.77 x 10^-7^, proportion of tests=0.0232, proportion of hits=0.0290), as well as significant under-enrichment in CMC DLPFC (p<2.20 x 10^-16^, proportion of tests=0.0398, proportion of hits=0.0171) and GTEx Testis tissues (p=0.02994, proportion of tests=0.0335, proportion of hits=0.0306).

***AN-GReX PheWAS***

***Allergies***

After QC, a total of 113 allergy phenotypes were tested for association with AN-GReX. In the overall Bio*Me*™ cohort, downregulation of *PFKFB4* was associated with allergy to both the antibiotic tetracycline (Skin Not Sun Exposed-*PFKFB4*, p=4.12 x 10^-6^) and gut motility drug metoclopramide (Overall-Brain cortex-*PFKFB4*, p=9.49 x 10^-6^). Similarly, in individuals with Mid and Low BMI, downregulation of *PFKFB4* was associated with allergy to tetracycline (Mid-Skin Not Sun Exposed-*PFKFB4*, p=1.13 x 10^-5^; Mid-Skin Sun Exposed-*PFKFB4*, p=2.90 x 10^-4^; Low-Skin Not Sun Exposed-*PFKFB4*, p=4.51 x 10^-4^). Among females, downregulation of *PFKFB4* was similarly associated with metoclopramide (Female-Brain, Cortex-*PFKFB4*, p=3.0 x 10^-6^). Downregulation of *PFKFB4* in individuals with Mid BMI was also associated with allergy to the diabetic drug metformin (Mid-Lung-*PFKFB4*, p=1.44 x 10^-4^) and upregulation to the analgesic acetaminophen (Mid-Esophagus Mucose-*PFKFB4*, p=9.28 x 10^-6^). Additionally, downregulation of *PFKFB4* was associated with allergy to the antibiotic vancomycin in High BMI individuals (High-Brain Cortex-*PFKFB4*, p=9.89 x 10^-5^; High-Cells Transformed lymphocytes-*PFKFB4*, p=3.37 x 10^-4^). Upregulation of *SLC2A10* was associated with allergy to morphine in the overall cohort (Colon transverse-*SLC2A10*, p=1.85 x 10^-5^; Esophagus Mucosa-*SLC2A10*, p=8.10 x 10^-5^). Among females, upregulation of the celiac-associated *CLIC1* gene was associated with a gluten allergy (Female-Multiple tissues-*CLIC1*, p<5.5 x 10*-4*). Additional allergy associations are provided in **Table S12**.

***Encounter Orders***

Encounter orders are coded orders from the EHR for various laboratory tests and procedures, dietary restrictions and other medication orders. After QC, there were a total of 1609 unique encounter order codes tested for association with AN-GReX. Full summary statistics for encounter orders are provided in **Table S13**.

In the overall cohort, downregulation of *ARIH2*, *NCKIPSD* and *DALRD3*, and upregulation of *CCDC71, SPINK8* and *WDR6* were associated with encounter order “DIET24”, an order for a regular diet (Heart Left Ventricle-ARIH2, p=8.95 x 10^-6^; Multiple tissues-*NCKPISD*, p<2.21 x 10^-5^; Cells Transformed Lymphocytes-*DALRD3*, p=8.33 x 10^-6^; Brain Frontal Cortex-*CCDC71*, p=1.75 x 10^-5^; Brain Hippocampus-*SPINK8*, p=1.21 x 10^-5^; Brain Frontal Cortex-*WDR6*, p=1.02 x 10^-5^; Brain Putamen basal ganglia-*WDR6*, p=7.59 x 10^-6^; Brain Substantia Nigra-WDR6, p=3.79 x 10^-6^). *ARIH2*, *CCDC71* and *WDR6* were additionally associated with encounter order “OPH1122”, coded for “Ophthalmoscopy subsequent performed”, along with upregulation of *CCDC36* (Upregulation-Heart Left Ventricle-ARIH2, p=2.17 x 10^-5^; Upregulation-Multiple tissues-*CCDC36*, p<1.95 x 10^-5^; Downregulation-Multiple tissues-CCDC71, p<2.09 x 10^-5^; Downregulation-Brain Putamen basal ganglia-WDR6, p=1.84 x 10^-5^). Among females, downregulation of *CCDC71*, *GPX1*, *SLC26A6*, and *WDR6*, and upregulation of *ARIH2*, *CCDC36*, *DALRD3*, *NCKIPSD*, *NDUFAF3*, *P4HTM*, *RP11-3B7.1*, were also associated with the “OPH1122” order code (Female-Multiple tissues-Multiple genes, p<3.8 x 10^-5^). Additional encounter orders associated with AN-GReX in the overall cohort include order “51701” for insertion of a bladder catheter, “97112” for Neuromuscular re-education, “96372” for therapeutic, prophylactic and diagnostic injections and infusions, “100696” for a pregnancy-induced hypertension panel, “102915” for IFE interpretation, “600536” for pelvic ultrasound and “NUR577” for calorie count (**Table S13**).

Downregulation of *ARIH2*, *CCDC36*, and *DALRD3* and upregulation of *CCDC71* and *WDR6* (all of which were all associated with “DIET24” in the overall cohort), were associated with encounter order “PRE5” for fall precautions in individuals with Low BMI (p<3.85 x 10^-5^; **Table S13**). Additional associations within individuals of Low BMI were downregulation of *SLC26A10* with order “100478” for surveillance of multi-drug-resistant gram-negative bacterial respiratory infection (Low-Liver-*SLC26A10*, p=1.70 x 10^-5^), downregulation of *FBLIM1* with code “600536” for non-obstetric pelvic ultrasound (Low-DLPFC-*FBLIM1*, p=3.49 x 10^-5^), and downregulation of *DALRD3* with “DIET13” for a clear liquid diet (Low-Liver-*DALRD3*, p=2.98 x 10^-5^). In individuals with High BMI, upregulation of *ARIH2* was associated with code “82565” for a creatinine serum lab order (High-Brain Amygdala-*ARIH2*, p=2.87 x 10^-5^) and downregulation of *WDR6* and *TMEM89* was associated with code “51701” for bladder catheter insertion (High-Artery Aorta-*TMEM89*, p=1.77 x 10^-5^; High-Thyroid-*WDR6*, p=2.35 x 10^-5^). Additional encounter order codes associated with AN-GReX in individuals with High BMI were “DIET50” for a renal diet, “CON65” for a consult to social worker, “PR157” for post-diagnostic catheter nursing intervention, “PRL166” for physician notification, “101186” for an abdominal pain panel, “NUR17” for pain assessment, “82040” for albumin blood measurement, and “NUR463” for obtaining height and weight measures (**Table S13**).

***Family History***

After QC a total of 144 phenotypes were available from family history data derived from the EHR. Family history phenotypes were initially collapsed based on degree of relatedness into “first degree”, “second degree” and “third degree” categories (see **Supplementary Methods**). Upregulation of *MGMT* was associated with a first-degree family history of HIV infection in both the overall cohort (p<3.33 x 10^-4^) and in individuals with low BMI (p<7.93 x 10^-4^) across multiple tissues. This association passed the phenotype-wide threshold of p=7.72 x 10^-6^ in individuals with low BMI (Low-Brain Hippocampus-*MGMT*, p=6.93 x 10^-7^; Low-Subcutaneous Adipose-*MGMT*, p=7.53 x 10^-6^). Additional tissue-specific significant results are shown in **Table S14**.

***Lipids (Total cholesterol, HDL, and LDL measures)***

We looked at the association of AN-GReX with three different measures of cholesterol: total cholesterol, HDL cholesterol, and LDL cholesterol, all measured as mg/dL. For each cholesterol phenotype, we looked at the highest and lowest recorded measures, as well as the mean measure (**Supplementary Methods**). All tissue-significant results are shown in **Table S6A**.

In order to determine which tissues may be driving the *MGMT*-GReX association in High BMI individuals with highest and mean cholesterol and LDL measures, we performed a stepwise conditional analysis within the High BMI group for *MGMT*-GReX with each phenotype. For each iteration, we conditioned on the top *MGMT*-GReX tissue association until there were no nominally significant *MGMT*-GReX associations (p<0.05). Results are shown in **Table S6B**. *MGMT*-Stomach, Liver, and Cells GReX was associated with highest cholesterol measure in High BMI individuals, whereas *MGMT*-Stomach and Esophagus, Mucosa GReX was associated with highest LDL cholesterol. *MGMT*-Brain GReX was associated with mean measures of total cholesterol and LDL cholesterol: *MGMT*-DLPFC GReX with mean cholesterol, and MGMT-Hippocampus with mean LDL measures (**Table S6B**).

***Medications***

After QC, a total of 951 unique medications/drug components were tested for association with AN-GReX. All associations with medications are shown in **Table S9**. In the overall cohort, upregulation of MHC-gene *CLIC1* was associated with multiple insulin- and diabetes-related medications including glucagon (Spleen-*CLIC1*, p=4.20 x 10^-9^), insulin aspart, insulin detemir, insulin degludec, insulin glulisine, insulin glargine, insulin lispro, insulin isophane, regular insulin and liraglutide (Subcutaneous Adipose-*CLIC1*, p<3.27 x 10^-5^). Among females, *CLIC1* was similarly associated with both glucagon (Female-Spleen-*CLIC1*, p=1.4 x 10^-8^) and insulin phenotypes (Female-Multiple tissues-*CLIC1*, p=9.6 x 10^-7^). Within the BMI-stratified groups, we find that *CLIC1* upregulation is similarly associated with glucagon in individuals in the Mid BMI group (Mid-Spleen-*CLIC1*, p=2.09 x 10^-6^), and the association of *CLIC1* GReX with glucagon remains fairly similar across BMI groups (**Figure S5**). Upregulation of *KREMEN1* and *RBM6*, and downregulation of *PFKFB4* were also associated with insulin- and diabetes-related phenotypes (Insulin aspart-Liver-*KREMEN1*, p=2.81 x 10^-5^; insulin glargine-Liver-*KREMEN1*, p=4.16 x 10^-5^; insulin isophane-Liver-*KREMEN1*, p=4.31 x 10^-5^; linagliptin-Skin Not Sun Exposed-*PFKFB4*, p=3.91 x 10^-5^; insulin isophane-Brain Substantia Nigra-*RBM6*, p=3.96 x 10^-5^).

In the overall cohort, downregulation of *RNF123* was associated with the benzodiazepine sedative temazepam (Artery Aorta-*RNF123*, p=2.76 x 10^-6^), stimulant laxative sennosides (Esophagus Mucosa-*RNF123*, p=1.33 x 10^-5^; Skeletal Muscle-*RNF123*, p=6.88 x 10^-6^), and diuretic hydrochlorothiazide (Esophagus Muscularis-*RNF123*, p=3.73 x 10^-5^). Upregulation of *KREMEN1* was also associated with the hydrochlorothiazide (Adipose Visceral Omentum-*KREMEN1*, p=4.55 x 10^-5^). Downregulation of *PFKFB4* was associated with type 2 diabetes medication empagliflozin (Skin Not Sun Exposed-*PFKFB4*, p=1.58 x 10^-5^) and anti-diarrheal loperamide (Skin Not Sun Exposed-*PFKFB4*, p=2.19 x 10^-5^). Additional overall AN-GReX associations with medications include antibiotic bacitracin (DLPFC-*C3orf62*, p=4.28 x 10^-5^), SSRI antidepressant sertraline (Brain Cerebellum-*MGMT*, p=2.53 x 10^-5^), analgesic salicylic acid (Brain Caudate basal ganglia-*NICN1*, p=1.90 x 10^-5^), HIV antiviral lopinavir (Brain Hippocampus-*SLC26A6*, p=1.72 x 10^-6^) and immunosuppressant chloroquine (Cells Transformed lymphocytes-*SLC2A10*, p=3.71 x 10^-5^).

Chemotherapy drug Paclitaxel is associated with upregulation of *SUOX* (Mid-DGN Whole Blood-*SUOX*, p=1.15 x 10^-6^) and upregulation of *PFKFB4* with anti-muscarinic drug mirabegron in Mid BMI individuals (Mid-Esophagus Mucosa-*PFKFB4*, p=1.77 x 10^-6^). In individuals of High BMI, downregulation of MST1 was associated with anti-diarrheal hormone octreotide (High-Adrenal gland-MST1, p=4.71 x 10^-6^) and downregulation of *PFKFB4* was associated with antidiabetic repaglinide and anti-tremor and gut antispasmodic Hyoscyamine (Repaglinide-High-Skin Sun Exposed-*PFKFB4*, p=7.92 x 10^-6^; Hyoscyamine-High-Skin Not Sun Exposed-*PFKFB4*, p=1.39 x 10^-5^). Among Low BMI individuals, upregulation of *GPX1* was associated with a prescription for lanolin oil (Low-Adipose Visceral Omentum-*GPX1*, p=8.25 x 10^-6^). Additionally, downregulation of *USP19* was associated with osteoporosis drug Teriparatide in individuals with Low BMI (Low-Pituitary-*USP19*, p=7.84 x 10^-6^).

Within the sex-stratified results, downregulation of *RNF123* and *MST1R* was associated with a prescription of the diuretic drug Hydrochlorothiazide in Females (p<5.9 x 10^-5^). Downregulation of *RNF123* and *MST1R*, and upregulation of *RBM6* were additionally associated with the anti-hypertensive drug Olmesartan among Females (p<4.8 x 10-5). Among males, *RP11-477N3.1* and *RNF123* were associated with multiple psychiatric drugs, including the antipsychotic Clozapine, benzodiazepines Lorazepam and Temazepam, and anxiolytic Buspirone (p<5.6 x 10^-5^). Additional associations of AN-GReX with medications are found in **Table S9**.

***OB/GYN History***

OB/GYN phenotypes were extracted from the EHR for female individuals and contained the following categories along with date of outcome: abortion, current pregnancy, ectopic pregnancy, para (parity), gravida, preterm birth, spontaneous abortion, to-term birth and therapeutic abortion. In the overall cohort, downregulation of *MST1* was associated with overall gravida (number of pregnancies) in multiple tissues (Overall-*MST1*, p<0.0034). Among Mid BMI individuals, *CLIC1*, *EBF3*, *MGMT* and *SUOX* were associated with preterm birth (p<0.0051). Upregulation of *RBM6* was associated with preterm birth among Low BMI individuals for multiple tissues (p<0.0061), while downregulation of *MST1R*, *RNF123*, and *SLC2A10* were associated with a term birth in Low BMI individuals (p<0.0059). Upregulation of *KREMEN1* in individuals with High BMI was associated with overall parity, or the number of births per gestation (Subcutaneous adipose-*KREMEN1*, p=1.16 x 10^-4^). Downregulation of *SLC26A10* was also associated with parity, but in individuals of low BMI (Subcutaneous adipose-*SLC26A10*, p=2.03 x 10^-5^). All associations are shown in **Table S16**.

***Pain Score and Location***

QC of pain score and pain location phenotypes are available in the **Supplementary Methods**. Final phenotypes available for pain score include highest ever pain score, sum of pain scores and mean pain score. Pain location resulted in a total of 24 location phenotypes: abdomen, ankle, back, breast, chest, costal left, costal right, elbows, feet, generalized, groin, hands, head, knees, left leg, lower extremities, neck, pelvis, right leg, sacrum, shoulder, throat, upper extremities and wrist. Pain score by location phenotypes included measures of highest ever, sum and mean pain scores for each location type. A total of 99 phenotypes (categorical and continuous) were available for pheWAS analyses.

Downregulation of *MGMT* was associated with mean pain score among High BMI individuals (High BMI-Minor salivary gland-*MGMT*, p=7.8 x 10^-5^), while upregulation of *SLC2A10* was associated with mean pain score among Low BMI individuals (Low BMI-Adrenal gland-*SLC2A10*, p=6.5 x 10^-5^). Downregulation of *MGMT* was associated with pain score sum in High BMI individuals (High BMI-Spleen-*MGMT*, p=1.85 x 10^-5^). Among males, downregulation of *CCDC36* in DGN whole blood was associated with mean pain score (p=3.1 x 10^-5^).

For measures of pain location only, many genes were associated with pain location in the overall cohort, as well as among the stratified BMI groups. In the overall cohort, upregulation of *SEMA3F, and* downregulation of *WDR6* were associated with throat pain (p<4.2 x 10^-4^). Upregulation of TUSC2 in Pancreas was associated with knee pain in both the overall cohort (p=7.6 x 10^-6^) and in females (p=3.9 x 10^-5^). Among Low BMI individuals, multiple genes were associated with foot pain (60 associations, p<5.0 x 10^-4^), generalized pain (13 associations, p<4.3 x 10^-4^), and elbow pain (p<4.6 x 10^-4^). All summary statistics for AN-GReX associations with pain location are located in **Table S8**.

When merging pain score with pain location, multiple AN genes were associated with measure of pain in various bodily locations (**Table S8**). In the overall cohort, multiple genes were associated with pain score sum in knees (14 associations, p<4.3 x 10^-4^). Upregulation of *CTNNB1*, *TMEM89* and *TNFSF12* was associated with throat pain score sum (Nerve Tibial-*CTNNB1*, p=4.92 x 10^-10^; Brain Cerebellar Hemisphere-*TMEM89*, p=2.39 x 10^-6^; Nerve Tibial-*TNFSF12*, p=8.20 x 10^-7^). Upregulation of *KREMEN1* was associated with highest ever elbow pain as well as mean elbow pain measures Upregulation of *EBF3* was associated with hand pain score sum, while downregulation of *PFKFB4* and *SPINK8* were associated with back pain score sum and costal left pain score sum respectively (**Table S8**).

Within the stratified groups, many genes were associated with various pain scores and locations. *MST1* downregulation was associated with highest neck pain score in Mid BMI individuals (Multiple tissues, p<4.00 x 10^-5^ ) (**Table S8**). Upregulation of *ARIH2*, *MST1* and *NICN1* in Low BMI individuals was associated with right leg pain score sum. In High BMI individuals, *CTNNB1*, *RP11-477N3.1* and *SPINK8* were associated with measures of knee pain, and *PFKFB4*, *SUOX* and *NICN1* were associated with back pain, head pain, and ankle pain respectively. Among females, many genes were associated with pain score measures in elbows and the sacrum (p<9.8 x 10^-6^), while in males there were multiple genes associated with pain score measures in the pelvis (p<1.2 x 10^-5^). All summary statistics for pain score by location can be found in **Table S8**.

***Personal History***

We tested a total of 35 phenotypes listed as “Personal History of Disease” in Bio*Me*™ (**Table S16**). Upregulation of *ARIH2*, *C3orf62*, *CCDC36*, *CELSR3*, *DALRD3*, *NCKIPSD*, *NDUFAF3*, *NICN1* and *P4HTM*, and downregulation of *CCDC71*, *GPX1* and *WDR6*, were associated with personal history of Lupus in the overall cohort, as well as in Females, and in individuals with Mid BMI, in multiple tissues (Overall, p<1.42 x 10^-3^; Mid, p<1.41 x 10^-3^) (**Figure S15**). All of these genes fall within the chromosome 3 locus from our S-PrediXcan results (**Figure 2, Table S3A**). Additionally, MHC-gene *CLIC1* was also associated with a personal history of Lupus in the overall cohort (Skeletal Muscle-*CLIC1*, p=7.57 x 10^-4^; Spleen-*CLIC1*, p=6.72 x 10^-4^; Subcutaneous Adipose-*CLIC1*, p=2.01 x 10^-4^). In individuals with Mid BMI, upregulation of *ARIH2*, *C3orf62*, *CCDC36*, *CELSR3*, *DALRD3*, *NCKIPSD*, *NICN1*, *P4HTM* and *USP19*, and downregulation of *CCDC71*, *GPX1*, *LAMB2*, and *WDR6*, was associated with a personal history of high blood pressure (p<1.42 x 10^-3^). Similarly, upregulation of *ARIH2*, *CCDC36*, *CELSR3*, *DALRD3*, *NCKIPSD*, *NDUFAF3*, *P4HTM* and downregulation of *WDR6* was associated with a personal history of kidney disease in individuals with Mid BMI (p<1.39 x 10^-3^). Among High BMI individuals, downregulation of *KREMEN1* was associated with a personal history of autism (High BMI-Multiple tissues-*KREMEN1*, p<0.001). Among females, downregulation of *KREMEN1* was also associated with Autism (Females-Multiple tissues-*KREMEN1*, p<9.1 x 10^-4^). Among females, *ARIH2*, *C3orf62*, *CCDC71*, *DALRD3*, *NCKIPSD*, *NDUFAF3*, *NICN1*, and *P4HTM* were associated with a personal history of ovarian cancer (Females-Multiple tissues-Multiple genes, p<0.0011). Within males, we saw multiple genes associated with a personal history of Alzheimer’s disease (30 associations, p<0.001), and inflammatory bowel disease (14 associations, p<0.0014). Additional associations are shown in **Table S15**.

***Social History* (additional results)**

Sample numbers for social history phenotypes can be found in **Tables S17B-E**. Phenotypes of sexual history, such as sexual activity, contraceptive use and type, as well as information about sexual partner, were derived from the social history questionnaire. A total of eight phenotypes were tested for association with AN-GReX*: (1) Sexually active (Y/N) (2) Self-reported female or male partner (3) Contraceptive method: abstinence, condom, injection, IUD, and pill (*phenotypes are categorized as Y/N or T/F). In the overall cohort, upregulation of *RBM6* and *SEMA3F* and downregulation of *MST1R* and *RNF123* were associated with being sexually active across multiple tissues (*RBM6*, p<5.46 x 10^-3^; *SEMA3F*, p<4.51 x 10^-3^; *MST1R*, p<4.60 x 10^-3^; *RNF123*, p<4.50 x 10^-3^). Similarly, among females, upregulation of *RBM6* and *SEMA3F*, and downregulation of *RNF123*, were significantly associated with being sexually active (p<1.1 x 10-4). Within our stratified BMI analyses, downregulation of *APEH* was associated with being sexually active in individuals of High BMI (High-Esophagus Muscularis-*APEH*, p=4.63 x 10^-5^). Additionally in individuals of High BMI, upregulation of *GPX1* was associated with IUD use (High-Esophagus Mucosa-*GPX1*, p=8.11 x 10^-5^).

We found multiple tissue-specific associations of AN-GReX with measures of substance use across all of the stratification groups. Downregulation of *MGMT* was associated with decreased alcohol use (ounces per week) in individuals in the Mid-BMI group (N=4963) (Mid BMI- Multiple tissues-*MGMT*, p<9.7 x 10^-4^; **Figure S8**), and with lower amounts of alcohol consumption in males (N_Males_=4227; p<1.4 x 10^-3^) (**Figure SX**). Additionally, upregulation of *DHFR2* and *KREMEN1* in females was associated with being an alcohol user (N_Female Cases_=7336) (p<4.6 x 10^-4^) (**Figure S3**). In individuals of Low BMI, predicted expression of *GPR75* and *GPX1* was associated with illicit drug use frequency (N_Low BMI_=219) (Liver-*GPR75*, p=3.34 x 10^-4^; Nucleus accumbens-*GPX1*, p=2.04 x 10^-4^; **Figure S3**). In females, downregulation of *GPX1* was associated with a lower likelihood of being an illicit drug user (N_Female Cases_=560) (Females-Brain, Cortex-*GPX1*, p=9.9 x 10^-4^; Females-Esophagus, Mucosa-*GPX1*, p=8.0 x 10^-4^), while upregulation of *C3orf62* was associated with greater illicit drug use frequency (N_Females_=17191) (Females-Visceral omentum adipose-*C3orf62*, p=1.5 x 10^-4^). All associations are described in **Table S7**.

***Vital signs***

Vital signs were measured for individuals at most if not all encounters with the healthcare system. Measurements of blood pressure, height, pulse, pulse oximetry, respirations, temperature and weight were recorded for each individual for each encounter. Vital sign measurements were standardized and QC of these measurements is described in the **Supplementary Methods**. Full results for vital signs are shown in **Table S11**.

*Blood Pressure*

Blood pressure measures were split into three factors: diastolic pressure, systolic pressure, and pulse pressure (see **Supplementary Methods**), with highest ever measure, lowest ever measure, mean measure and variance calculated for each phenotype. In the overall cohort, upregulation of *RBM6* and downregulation of *MST1R* were associated with systolic highest measurements (Systolic highest-Overall-Minor salivary gland-*RBM6*, p<3.4 x 10^-5^; Systolic highest-Overall-Skeletal muscle-*MST1R*, p<3.0 x 10^-5^). Downregulation of *MST1R* was associated with multiple measures of systolic, diastolic and pulse-pressure measures in the Overall cohort (p<8.4 x 10^-4^), and among females (p<9.8 x 10^-4^). Upregulation of NCKIPSD, *NDUFAF3*, and *P4HTM*, and downregulation of *WDR6* were associated with lowest systolic pressure in the overall cohort (multiple tissues, p<9.68 x 10^-4^). All associations are described in the full summary statistics in **Table S11**.

*Height*

Height for individuals in Bio*Me*™ was standardized to centimeters units of measure (see **Supplementary Methods**). In the overall cohort, there were four genes associated with measures of mean height. Upregulation of *CLIC1*, *GPR75* and *LINC00324* and downregulation of *CTNNB1* were associated with mean height (Adipose Subcutaneous-*CLIC1*, p=1.39 x 10^-5^; Adipose Subcutaneous-*GPR75*, p=3.51 x 10^-4^; Artery Tibial-*GPR75*, p=5.07 x 10^-4^; Skin Sun Exposed-*LINC00324*, p=6.51 x 10^-4^; Brain Amygdala-*CTNNB1*, p=2.28 x 10^-4^; Brain Caudate basal ganglia-*CTNNB1*, p=1.67 x 10^-4^; Brain Putamen basal ganglia-*CTNNB1*, p=4.65 x 10^-4^). *GPR75* and *CLIC1* upregulation were also associated with mean height among the Mid BMI individuals (Mid-Adipose Subcutaneous-*CLIC1*, p=4.31 x 10^-4^; Mid-Artery Tibial-*GPR75*, p=1.44 x 10^-4^). Among females, multiple genes were associated with mean height across multiple tissues (30 associations, p<9.8 x 10^-4^). Full summary statistics are available in **Table S11**.

*Pulse*

Pulse measurements of beats per minute (bpm) were measured as the other vital sign continuous measurements as highest ever, lowest ever, mean and variance (**Supplemental Methods**). Downregulation of *NCKIPSD* was associated with mean pulse in the overall cohort (Colon Transverse-*NCKIPSD*, p=3.0 x 10^-4^). Within Mid BMI individuals, *CCDC36*, *DALRD3*, *GPX1*, and *NICN1* were associated with lowest recorded pulse (8 associations, p<8.7 x 10^-4^). Among individuals of Low BMI, downregulation of *ARIH2* was associated with highest pulse, while downregulation of *MST1R* and *RNF123* was associated with mean pulse (Low-Liver-*ARIH2*, p=4.5 x 10^-4^; Low-Colon Sigmoid-*MST1R*, p=6.4 x 10^-4^; Low-Pituitary-*RNF123*, p=7.0 x 10^-4^). No significant results were found for pulse measures in our sex-stratified analyses. Full summary statistics are located in **Table S11**.

*Pulse Oximetry*

AN-GReX was not significantly associated with measures of pulse oximetry in the overall Bio*Me*™ cohort, however, when we stratified by BMI, we did see significant associations of AN-GReX with pulse oximetry among the BMI groups. Downregulation of *RBM6*, and upregulation of *SLC26A10* were associated with lowest pulse oximetry measure in Mid BMI individuals (Mid BMI-Brain, Caudate basal ganglia-*RBM6*, p=8.6 x 10^-4^; Mid BMI-Heart, Left ventricle-*SLC26A10*, p=5.4 x 10^-4^). Upregulation of *DHFR2* was associated with lowest pulse oximetry measurements in High BMI individuals (High-Cells, Transformed fibroblasts*-DHFR2*, p=4.1 x 10^-4^). In females, downregulation of *MST1R* was associated with measures of highest pulse oximetry (Females-Subcutaneous adipose-*MST1R*, p=4.9 x 10^-4^; Females-Esophagus, Mucosa-*MST1R*, p=5.5 x 10^-4^), and upregulation of *TNFSF12* in tibial nerve was associated with mean pulse oximetry (p=8.3 x 10^-4^). Full summary statistics are located in **Table S11**.

*Respirations*

Associations of AN-GReX with respirations, or breaths per minute, were measured using highest ever, lowest ever, mean and variance for the respiration phenotypes (**Supplemental Methods**). Downregulation of *CCDC36 and MST1R* and upregulation of *CCDC71 and WDR6*  were associated with measures of highest ever respiration rate in the overall cohort (Multiple tissues, p<9.84 x 10^-4^, **Table S11**). Additionally, downregulation of *CCDC36* and *P4HTM*, and upregulation of *GPX1*, were associated with mean respiration rate among the overall cohort (Multiple tissues, p<8.53 x 10^-4^). Among the BMI stratified groups, downregulation of *GPX1* and upregulation of *NICN1* is associated with lowest ever respiration rates in High BMI individuals (Multiple tissues, p<8.99 x 10^-4^). Among Low BMI individuals, we see similar patterns of association of AN-GReX with respirations as we saw in the overall cohort. Downregulation of *ARIH2*, *NCKIPSD*, *NDUFAF3*, *RP11-3B7.1*, and *CCDC36*, and upregulation of *CCDC71* are associated with highest respiration rate in the Low BMI individuals (Multiple tissues, p<9.93 x 10^-4^). Upregulation of *MST1* in DGN whole blood was associated with respiration variance among males (p=5.7 x 10^-4^). Downregulation of *MGMT* in females was associated with highest respirations measured (Female-Brain, Cerebellar hemisphere-*MGMT*, p=5.3 x 10^-4^). Full summary statistics are located in **Table S11**.

*Temperature*

Temperature measurements were standardized to degrees Celsius (**Supplemental Methods**), and highest ever, lowest ever, mean and variance calculated. Among individuals in the Mid BMI group, downregulation of *EBF3* was associated with highest ever temperature (DLFPC-*EBF3*, p=7.24 x 10^-4^; Ovary-*EBF3*, p=7.15 x 10^-4^). *PROS1* upregulation was associated with temperature variance among males (Males-Aorta artery-*PROS1*, p=4.3 x 10^-4^). Full summary statistics are located in **Table S11**.

*Weight*

Within the overall cohort, downregulation of *RP11-804H8.6* was associated with highest measured weight (Overall-Lung-*RP11-804H8.6*, p=0.0015). Across the stratified groups, different genes were associated with highest measured weight: among High BMI individuals, downregulation of *CTNNB1* and *SLC26A10* (High BMI-Colon, Transverse-*CTNNB1*, p=6.7 x 10^-4^; High BMI-Tibial artery-*SLC26A10*, p=0.0014), among males, downregulation of *P4HTM*, and upregulation of *PFKFB4* were associated with highest measured weight (Males-Cells, Transformed fibroblasts-*P4HTM*, p=9.4 x 10^-4^; Males-Lung-*PFKFB4*, p=7.7 x 10^-4^). Among Low BMI individuals, *PROS1* downregulation was associated with lowest recorded weight (Low BMI-CommonMind DLPFC-*PROS1*, p=0.0012). For measures of weight change over time, upregulation of *SLC2A10* in the overall cohort, and upregulation of *MST1R* in the Mid BMI individuals were associated with weight change over time (Overall-Tibial nerve-*SLC2A10*, p=0.0013; Mid BMI-Colon, Sigmoid-*MST1R*, p=5.1 x 10*-4*) (**Table S11; Figure S6**).

***Context-specific BMI results***

In order to test whether our context-specific results were being driven by the effect of BMI on AN-GRex, rather than the direct effect of BMI on the context-specific phenotype, we ran our context-specific Social history phenotypes (Alcohol consumption, Illicit drug use, and Tobacco use) using BMI, highest recorded weight (kg) and lowest recorded weight (kg) as predictors. Our results are shown in **Figure S4B**. For measures of alcohol consumption, BMI and highest recorded weight were associated with ounces of alcohol consumed per week (p<7.4 x 10^-4^) at the tissue-specific threshold of significance (p<0.003). None of the phenotypes were associated with frequency of illicit drug use (p>0.10). Only highest weight was associated with number of years of tobacco use (p=1.0 x 10^-4^). BMI, highest weight, and lowest weight were significantly associated with the number of cigarette packs smoked per day (p<1.2 x 10^-5^).

***AN Concordance***

Predicted gene expression of AN genes in our biobank cohort allows us to probe how aberrant AN gene expression affects clinical outcomes. We can ask, for those individuals whose AN-GReX is most similar to the GReX of someone with anorexia nervosa, which clinical outcomes do they present with? What about for individuals whose AN-GReX is dissimilar from someone with AN? What do their clinical outcomes look like? In order to assess how similar or dissimilar our AN-GReX biobank associations are as compared to individuals with AN, we compared the direction of effect (DoE) for each gene-tissue-pheWAS association with the DoE of that gene-tissue pair for AN from our S-PrediXcan results (**Figure 4; Supplemental Tables**). Gene-tissue-phenotype associations with the same DoE as the gene-tissue-AN association were marked as “concordant with AN”, while those with opposite DoEs were marked as “not concordant”. To visualize the overall concordance/discordance of our pheWAS results with AN, for each experiment-wide significant phenotype, we took the proportion of association results with the same DoE as AN divided by the total number of associations for that phenotype.

$$\boldsymbol{Concordance proportion=}\frac{Number of phenotype associations with the same gene\_tissue DoE as AN}{Total number of phenotype associations}$$

For example, in the Overall cohort, we have seven associations of AN-GReX with Celiac disease. All of those associations have a positive DoE for the gene *CLIC1* with Celiac disease across multiple tissues. The association of *CLIC1* GReX with AN for all of those tissues is also a positive DoE. Thus, for the overall cohort, the number of phenotype associations with the same *CLIC1*-tissue DoE as AN is 7/7 or 1. For phenotypes where none of the associations are in the same direction as AN, the concordance proportion is equal to zero. **Figure 4** shows concordance results for all experiment-wide significant phenotypes across all of the phenotypes tested. For those phenotypes concordant with AN, this may indicate that genetic regulation of those AN genes is more similar to individuals with AN in individuals with those clinical outcomes.

**SUPPLEMENTARY FIGURES AND TABLES**

Figure S1: BMI stratification of the Bio*Me*™ cohort. We stratified the Bio*Me*™ individuals into three BMI groups: High, Mid and Low, based on the distribution of BMI within each ancestry by sex. (A) Individuals whose BMI fell above the 3^rd^ quartile of BMI distribution were assigned to the High BMI group, those whose BMI fell below the 1^st^ quartile were assigned to Low, and those between the 1^st^ and 3^rd^ quartiles of the distribution were assigned to the Mid BMI group. (B) BMI distributions are plotted by sex (female, male) for each ancestry group. Ancestry is coded as: AA (African), EA (European), ESA (East Asian), HA (Hispanic), NA (Native American), SAS (South Asian).

Figure S2: Distribution of simulated gene expression to test for case contamination. Simulated gene expression distributions for 10,000 permutations at varying levels of contamination rate (*p*) and effect on GReX ($\boldsymbol{\beta}$) for 1000 cases (X) and 1000 controls (Y). (A) p=0.01, $\boldsymbol{\beta}$=0.1 (B) p=0.1, $\boldsymbol{\beta}$=0.5 (C) p=0.001, $\boldsymbol{\beta}$=1 (D) p=0.4, $\boldsymbol{\beta}$=0.25.

Figure S3: Diagnosis code associations across stratification groups. Effect estimates and 95% CI for FDR-significant associations of AN-GReX with (A) Encounter Diagnosis ICD-10 codes across the BMI stratified groups, Overall, High-BMI, Mid-BMI, and Low-BMI, and (B) across sex-stratified groups. (C) Phecode associations across the BMI stratification groups: Overall, High-BMI, Mid-BMI, and Low-BMI and (D) sex-stratified groups. Diagnosis code and description and gene-tissue given on the Y-axis. Experiment-wide significance threshold set at p<3.4 x 10^-7^. *High-BMI and Males are missing celiac phecode associations, and High- and Low-BMI groups are missing immunodeficiency diagnoses due to low numbers of cases (N_eff_<100) within those groups.

**Figure S4: Forest plot of substance use AN-GReX associations across stratification groups**. Effect estimates and 95% CI for the associations of (A) AN-GReX with substance use phenotypes across the stratification groups: Overall, High-BMI, Mid-BMI, Low-BMI, Females, and Males. Bonferroni experiment-wide significance set at p<3.6 x 10^-5^ [31 phenotypes * 45 tissues]. Tissue-specific significance set at p<0.0016 [31 phenotypes]. (B) Context-specific substance use results including BMI, highest recorded weight, and lowest recorded weight predictors.

Figure S5: Experiment-wide significant AN-GReX associations with medications across stratification groups. Effect estimates and 95% CI for the associations of (A) *CLIC1*-Spleen GReX with Glucagon, and *RP11-849F2.8*-Esophagus, Mucosa GReX with Clopidogrel medications across the four BMI groups: Overall, High-BMI, Mid-BMI and Low-BMI. (B) CLIC1-GReX associations with Glucagon and Insulin medications across sex-stratified groups. Bonferroni experiment significance set at p<1.2 x 10^-6^ [951 phenotypes * 45 tissues]. Tissue-specific significance threshold set at p<5.26 x 10^-5^ [951 phenotypes].

Figure S6: AN-GReX associations with weight phenotypes across stratification groups. Effect estimate and 95% CI measures for AN-GReX associated with the measures of highest recorded weight (kg), lowest recorded weight (kg), and weight change over time (kg/year) across BMI- and sex-stratified groups (Overall, High-BMI, Mid-BMI, Low-BMI, Females, and Males). Bonferroni experiment significance set at p<3.5 x 10^-5^ for Vital sign phenotypes [32 phenotypes * 45 tissues]. Tissue-specific significance threshold set at p<0.0016 [32 phenotypes].

Figure S7: Descriptive statistics for vital sign and lipid phenotypes. Descriptive statistics for lipid phenotypes of (A) LDL cholesterol, (B) HDL cholesterol, and (C) total cholesterol. Vital sign measurements for (D) pulse (beats per minute), (E) pulse oximetry (%), (F) respirations (breaths per minute), (G) temperature (degrees Celsius), (H) weight (kg) measures, and (I) height (cm).

**Table S1: Bonferroni-adjusted significance thresholds for each tissue model tested for S-PrediXcan**. Significance thresholds for CMC (1), DGN (1), and GTEx (48) tissues. The number of genes tested per tissue, with p value for Bonferroni tissue-specific significance set at p=0.05/N_Genes_, is indicated for each tissue. Experiment-wide significance threshold set at p<3.75x10^-8^ (0.05/(26364 genes * 50 tissues)).

**Table S2: Bio*Me*™ Demographics**. Sample numbers, mean BMI, and mean age for each ancestry group and sex in the Bio*Me*™ cohort (N=30385). BMI values used for determining stratification assignment to High-BMI, Mid-BMI, and Low-BMI are also shown. High-BMI range indicates the values above the 3^rd^ quartile of the BMI distribution within each ancestry and sex group; Low-BMI indicates values below the 1^st^ quartile, and Mid-BMI indicates the BMI range between the 1^st^ and 3^rd^ quartiles.

**Table S3: PGC AN-GWAS S-PrediXcan results**. (A) Gene-level summary statistics for transcriptomic imputation of AN-GWAS (N_Cases_=16992, N_Congrols_=55525) using S-PrediXcan models for CMC DLPFC, DGN whole blood, and 48 GTEx v7 tissues. Bonferroni-adjusted tissue-specific threshold set at p<2.45 x 10^-5^ (See **Table S1**). Bonferroni experiment-wide significance set at p<3.75 x 10^-8^. Experiment-significant genes are indicated in bold. (B) Direction of effect and summary statistics of S-PrediXcan AN-genes across all tissues tested, regardless of significance. (C) Calculated variance in predicted GReX for all AN-genes in CMC DLPFC, DGN whole blood, and GTEx tissues in the Bio*Me*™ cohort. Gene-tissue pairs in bold indicate GReX with very low variance (gVAR<0.002), which were subsequently removed from pheWAS analyses.

**Table S4: AN-Gene descriptions and gene set enrichment**. (A) Summary description of the 53 significant S-PrediXcan AN-genes. (B) FUMA GENE2FUNC gene set enrichment results for 52 AN-genes. Background genes selected were all genes (N=28454) tested in the initial S-PrediXcan analysis. P value of association of a gene set with AN-genes is FDR-BH adjusted (adjP) at a significance threshold of adjP<0.05.

**Table S5: Diagnosis code pheWAS results**. GReX-Tissue-Phenotype associations for encounter diagnosis (N=2178) and phecode (N=1093) phenotypes across all stratification groups: Overall, High-BMI, Mid-BMI, Low-BMI, Females and Males. Encounter diagnosis and Phecode results, due to their non-independence, were combined and FDR-adjusted for an Experiment-wide threshold (FDR-adjusted p<0.05, p<3.4 x 10^-7^. All associations had a meta-analysis heterogeneity score (pHet) > 0.001, and an effective sample size N_Eff_>100.

**Table S6: Lipid pheWAS results and conditional analysis**. (A) GReX-Tissue-Phenotype associations for highest measured, lowest measured and mean measures of Cholesterol, HDL and LDL across all stratification groups: Overall, High-BMI, Mid-BMI, Low-BMI, Females, and Males. Bonferroni tissue-specific threshold set at p<0.0056 [0.05/9 phenotypes]; Experiment-specific threshold set at p<1.2 x 10^-4^ [0.05/(9 phenotypes * 45 tissues)]. (B) Step-wise conditional analysis results for highest cholesterol and LDL, and mean cholesterol and LDL measures with MGMT-GReX in High-BMI individuals. All associations had a meta-analysis heterogeneity score (pHet) > 0.001.

**Table S7: Social History pheWAS results**. GReX-Tissue-Phenotype associations for Social History phenotypes (Alcohol, Illicit Drug and Tobacco use; Sexual History) across all stratification groups: Overall, High-BMI, Mid-BMI, Low-BMI, Females, and Males. Bonferroni tissue-specific threshold set at p<0.0016 [0.05/31 phenotypes]; Experiment-wide threshold set at p< 3.6 x 10^-5^ [0.05/(31 phenotypes * 45 tissues)].. All associations had a meta-analysis heterogeneity score (pHet) > 0.001. All categorical phenotypes had an effective sample size N_Eff_>100.

**Table S8: Pain Score and Location pheWAS results**. GReX-Tissue-Phenotype associations for Pain score and location phenotypes (N=99) across all stratification groups: Overall, High-BMI, Mid-BMI, Low-BMI, Females, and Males. Bonferroni tissue-specific threshold set at p<5.05 x 10^-4^ [0.05/99 phenotypes]; Experiment-specific threshold p< 1.12 x 10^-5^ [0.05/(99 phenotypes * 45 tissues)]. All associations had a meta-analysis heterogeneity score (pHet) > 0.001. All categorical phenotypes had an effective sample size N_Eff_>100.

**Table S9: Medication pheWAS results**. GReX-Tissue-Phenotype associations for medication phenotypes (N=951) across all stratification groups: Overall, High-BMI, Mid-BMI, Low-BMI, Females, and Males. Tissue-specific threshold set at p<5.26 x 10^-5^ [0.05/951 phenotypes]. Experiment-wide significance threshold p<1.17 x 10^-6^ [0.05/(951 phenotypes * 45 tissues)]. All associations had a meta-analysis heterogeneity score (pHet) > 0.001, and an effective sample size N_Eff_>100.

**Table S10: Expected association levels due to diagnostic contamination (ie, PheWAS trait cases with missing, undeclared or unknown AN diagnoses).**

**Table S11: Vital sign pheWAS results**. GReX-Tissue-Phenotype associations for Vital Sign phenotypes (Blood pressure, Height, Pulse, Pulse Oximetry, Respirations, Temperature and Weight) across all stratification groups: Overall, High-BMI, Mid-BMI, Low-BMI, Females, and Males. Bonferroni-adjusted tissue-specific threshold set at p<0.0016 [0.05/32 phenotypes]; Experiment-wide threshold set at p<3.47x10^-5^ [0.05/(32 phenotypes*45 tissues)]. All associations had a meta-analysis heterogeneity score (pHet) > 0.001.

**Table S12: Allergy pheWAS results**. GReX-Tissue-Phenotype associations for allergy phenotypes (N=113) across all stratification groups: Overall, High-BMI, Mid-BMI, Low-BMI, Females, and Males. Tissue-specific threshold set at p<4.42 x 10^-4^ [0.05/113 phenotypes]. Experiment wide-threshold of significance p<9.8 x 10^-6^ [0.05/(113 phenotypes * 45 tissues)]. All associations had a meta-analysis heterogeneity score (pHet) > 0.001, and an effective sample size N_Eff_>100.

**Table S13: Encounter orders pheWAS results**. GReX-Tissue-Phenotype associations for Encounter order phenotypes (N=1609) across all stratification groups: Overall, High-BMI, Mid-BMI, Low-BMI, Females, and Males. Tissue-specific threshold set at p<3.11 x 10^-5^ [0.05/1609 phenotypes]. Experiment-wide significance threshold p<6.9 x 10^-7^ [0.05/(1609 phenotypes * 45 tissues)]. All associations had a meta-analysis heterogeneity score (pHet) > 0.001, and an effective sample size N_Eff_>100.

**Table S14: Family History pheWAS results**. GReX-Tissue-Phenotype associations for Family history phenotypes (N=144) across all stratification groups: Overall, High-BMI, Mid-BMI, Low-BMI, Females, and Males. Tissue-specific threshold set at p<3.47 x 10^-4^ [0.05/144 phenotypes]. Experiment-wide significance threshold set at p<7.7 x 10^-6^ [0.05/(144 phenotypes * 45 tissues)]. All associations had a meta-analysis heterogeneity score (pHet) > 0.001, and an effective sample size N_Eff_>100.

**Table S15: Personal History pheWAS results**. GReX-Tissue-Phenotype associations for Personal history phenotypes (N=35) across all stratification groups: Overall, High-BMI, Mid-BMI, Low-BMI, Females, and Males. Tissue-specific threshold set at p<0.0014 [0.05/35 phenotypes]. Experiment-wide significance threshold set at p<3.17 x 10^-5^ [0.05/(35 phenotypes * 45 tissues)]. All associations had a meta-analysis heterogeneity score (pHet) > 0.001, and an effective sample size N_Eff_>100.

**Table S16: OB/GYN pheWAS results**. GReX-Tissue-Phenotype associations for OB/GYN phenotypes (N=9) across all BMI groups within Females: Overall, High-BMI, Mid-BMI, and Low-BMI. Tissue-specific threshold set at p<0.006 [0.05/9 phenotypes]. Experiment-wide significance threshold set at p<1.2 x 10^-4^ [0.05/(9 phenotypes * 45 tissues)]. All associations had a meta-analysis heterogeneity score (pHet) > 0.001, and an effective sample size N_Eff_>100.

**Table S17: Sample Numbers for OB/GYN and Social History phenotypes**. Sample numbers by phenotype for each ancestral group are shown for (A) Categorical OBGYN phenotypes, (B) Alcohol use, (C) Illicit drug use, (D) Tobacco use, and (E) Sexual history.
