# Supplementary figures and images for: Mapping anorexia nervosa genes to clinical phenotypes
Source: Psychol Med. 2022 Apr 5;53(6):2619–33. doi: 10.1017/S0033291721004554 (PMC10123844; doi:10.1017/S0033291721004554)

Figure S1

A

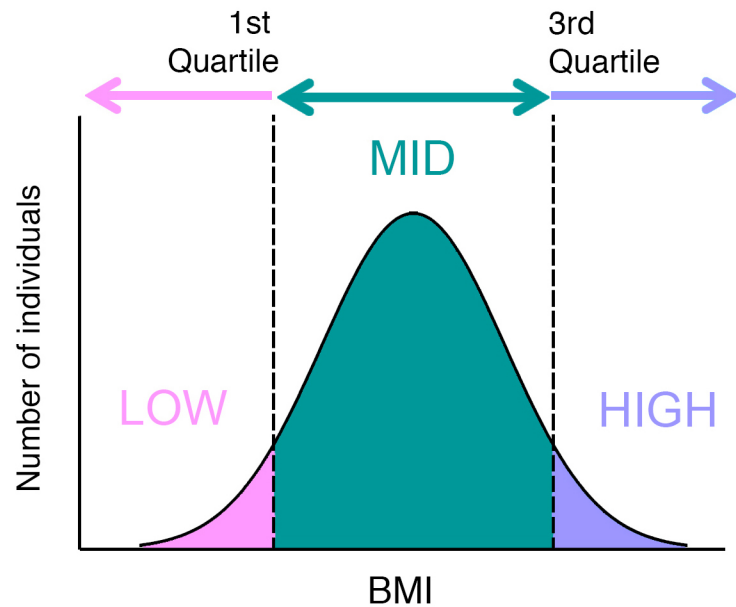

B

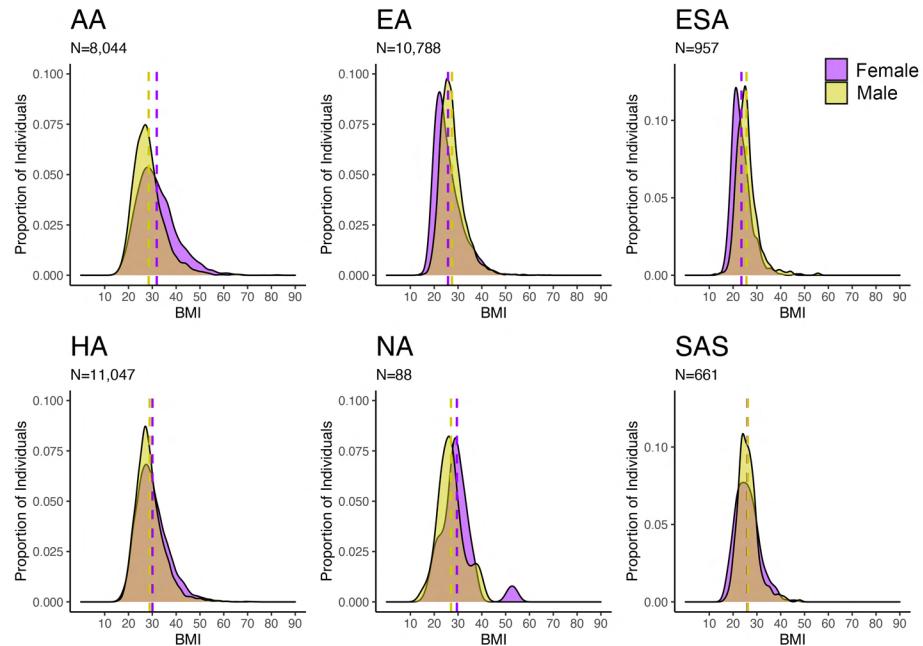

Figure S2A

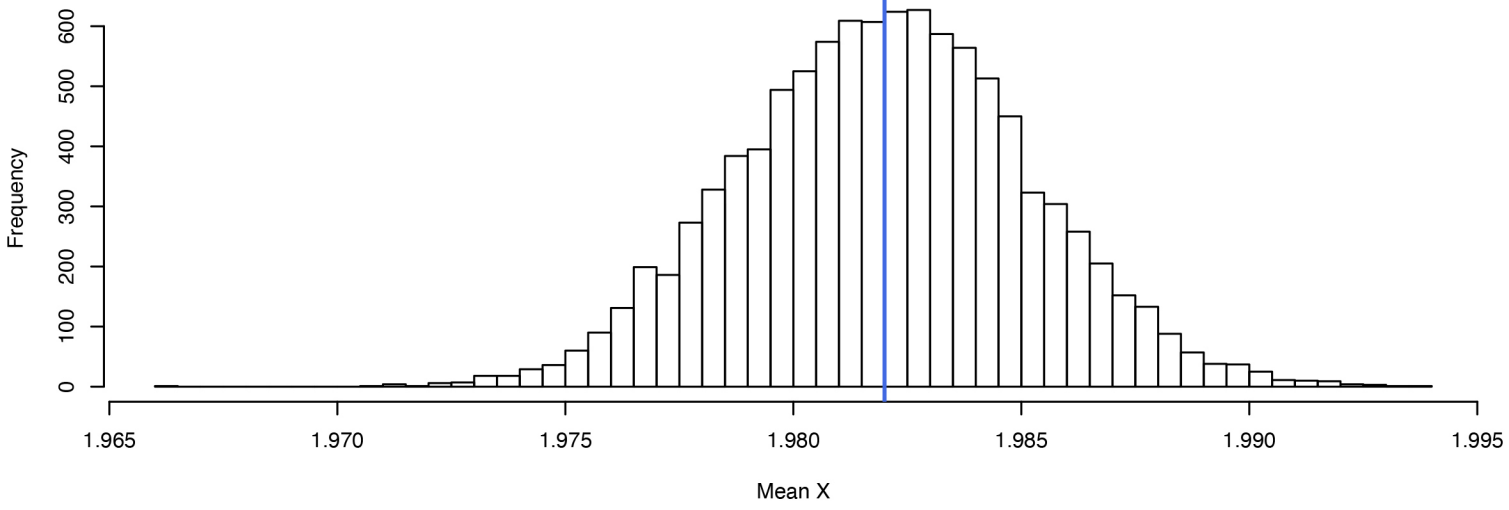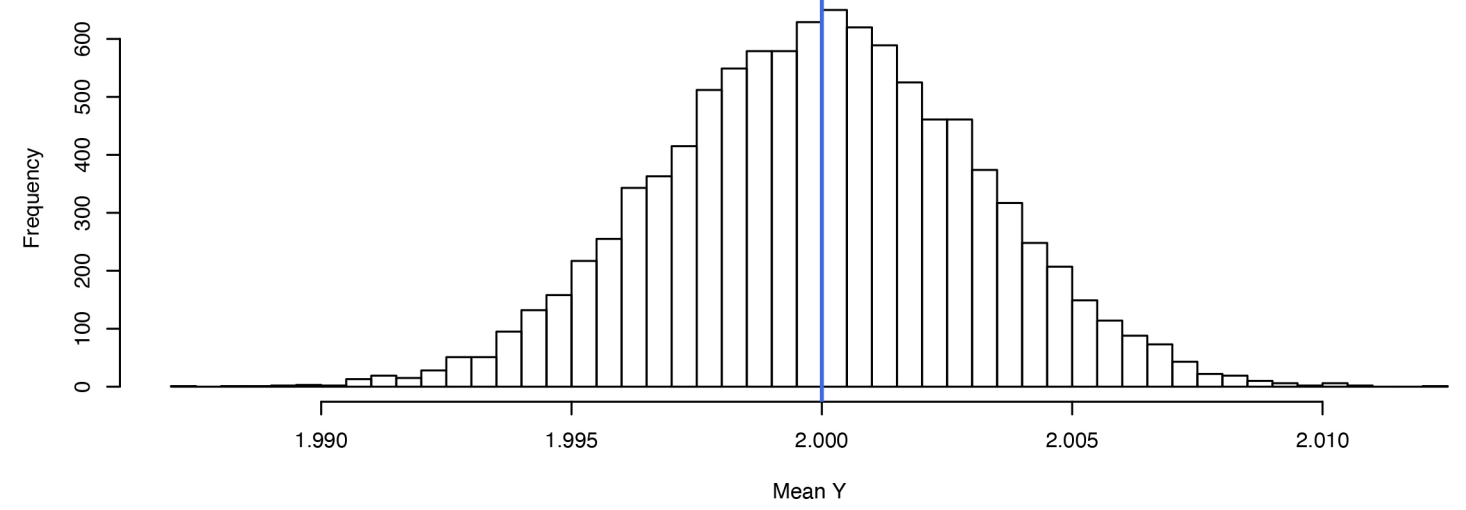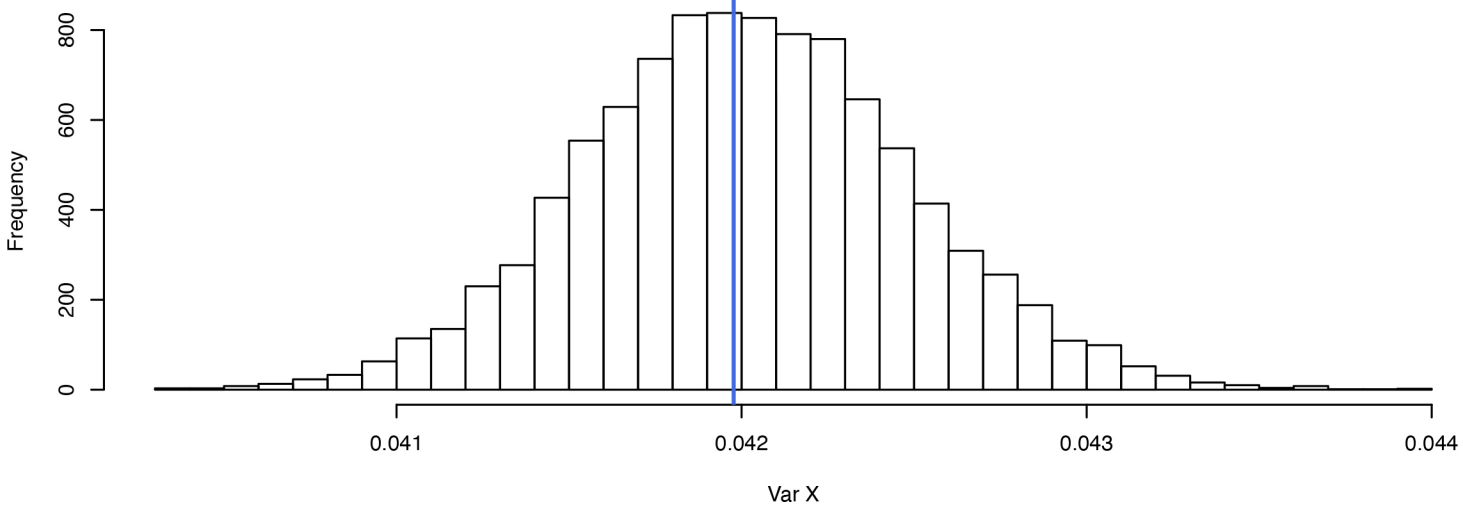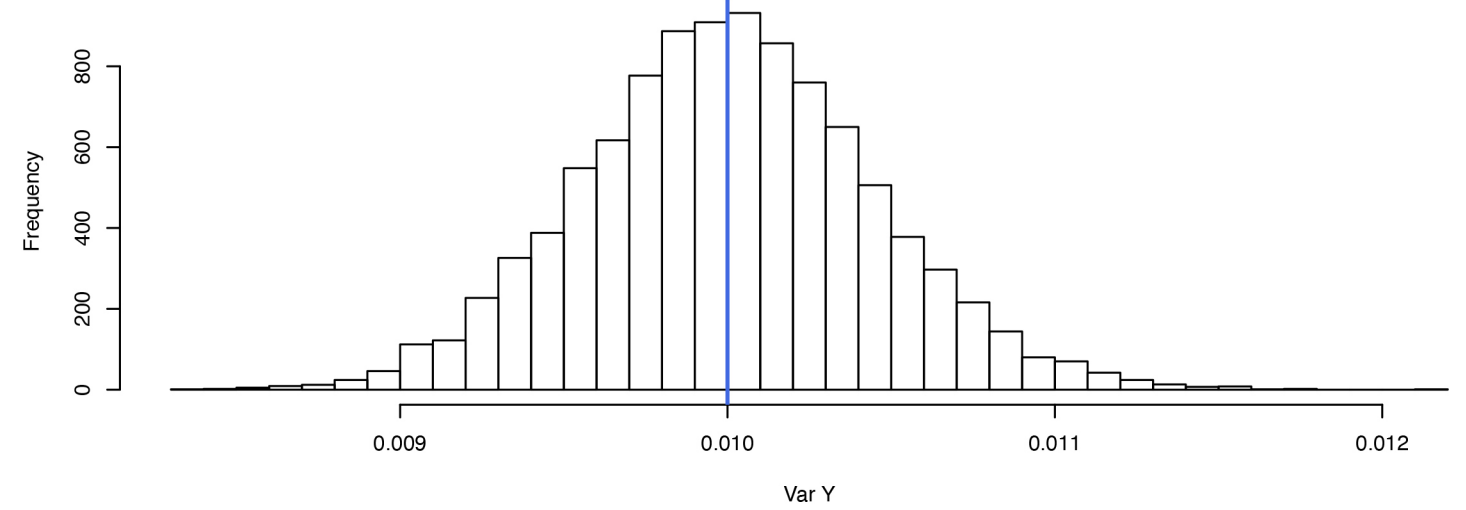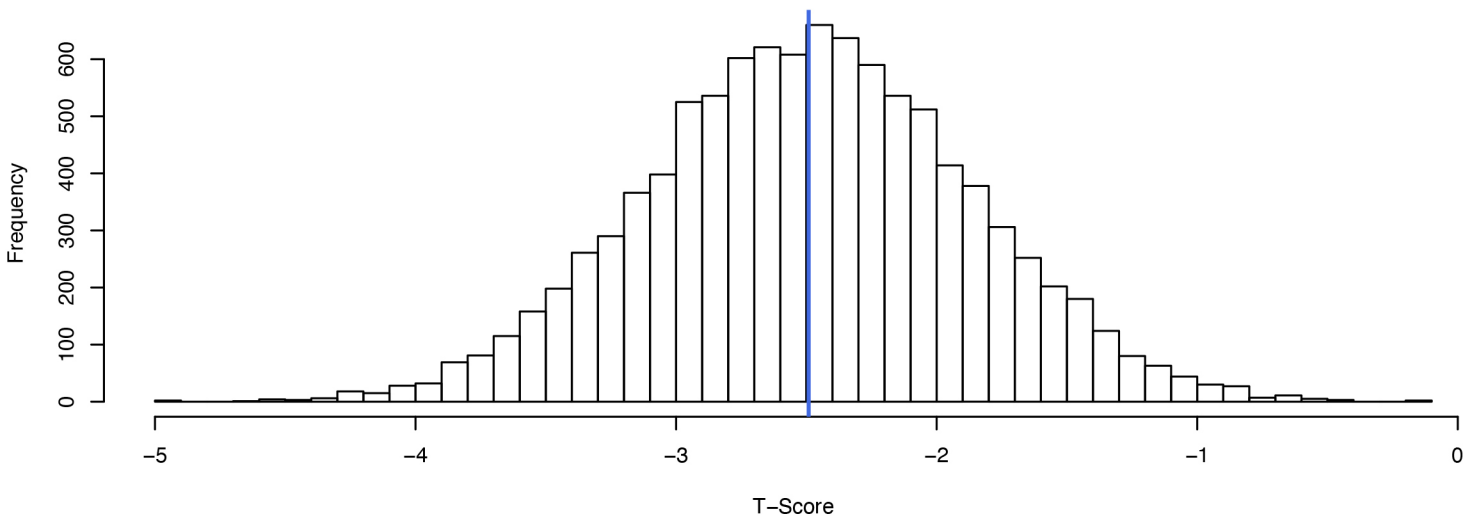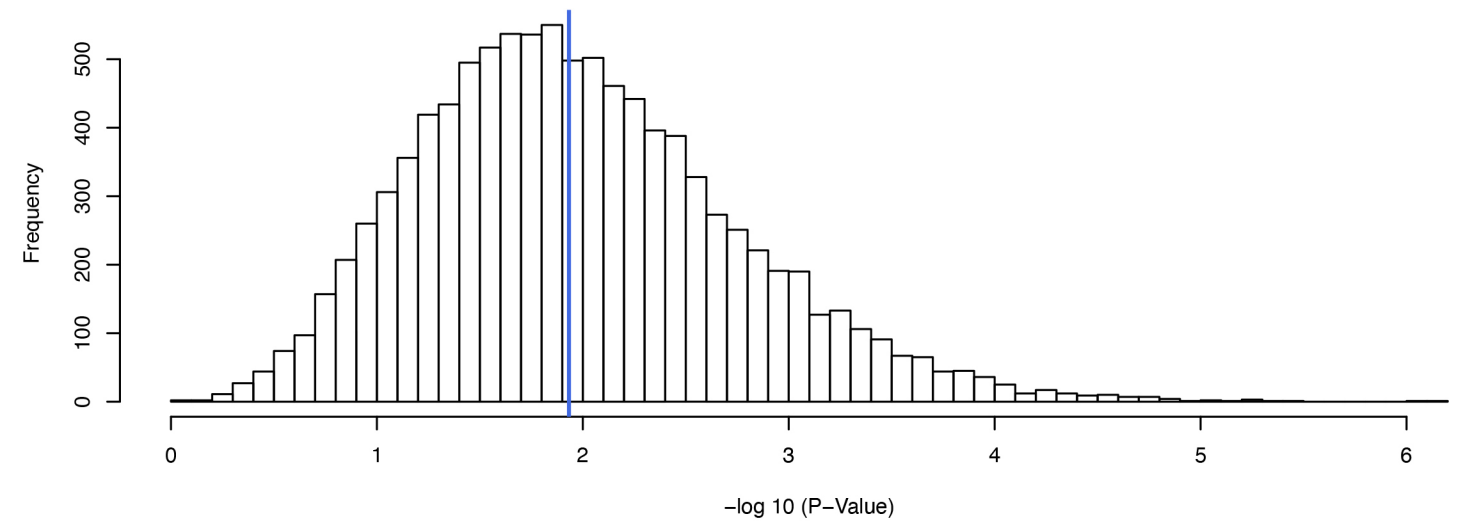

Figure S2B

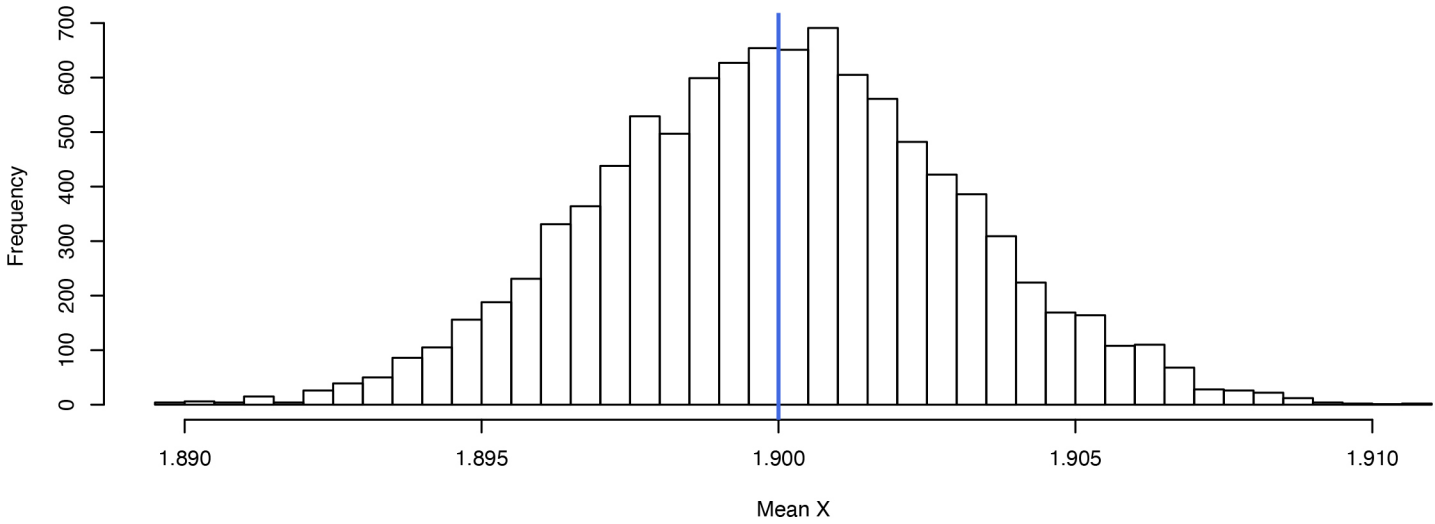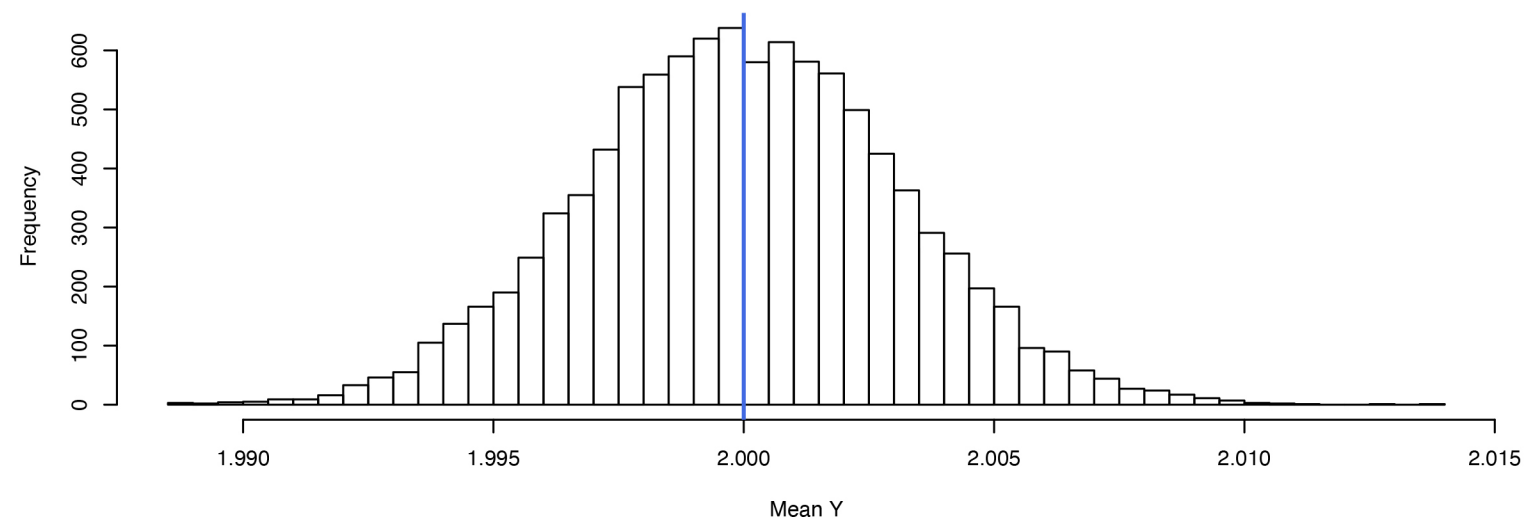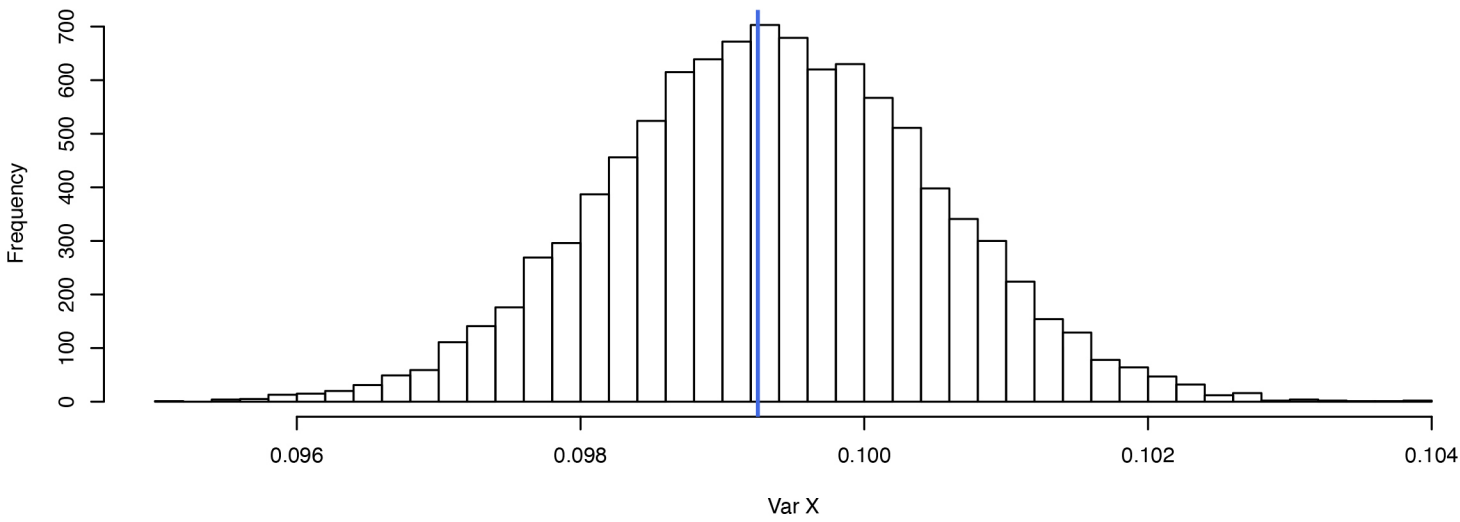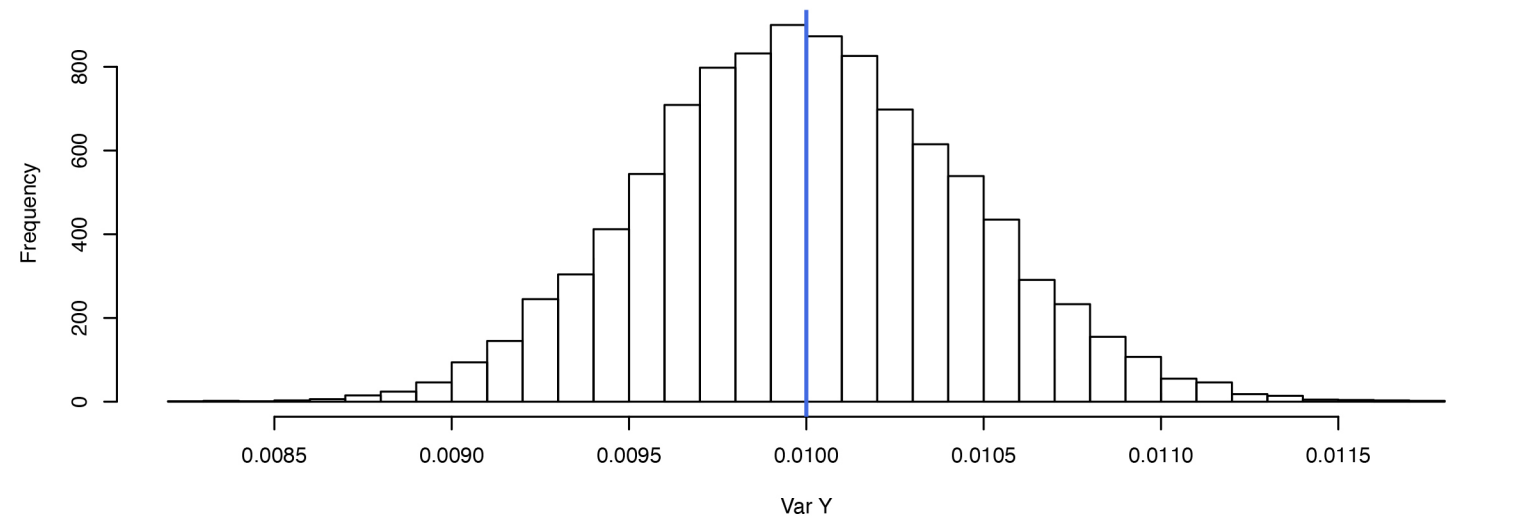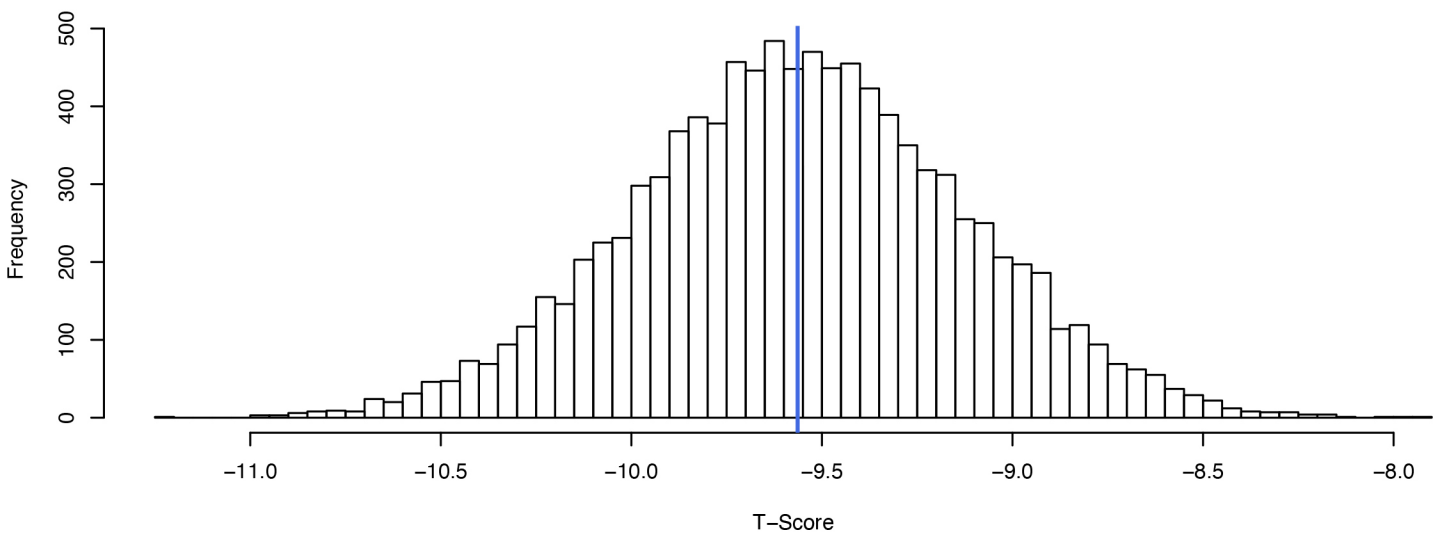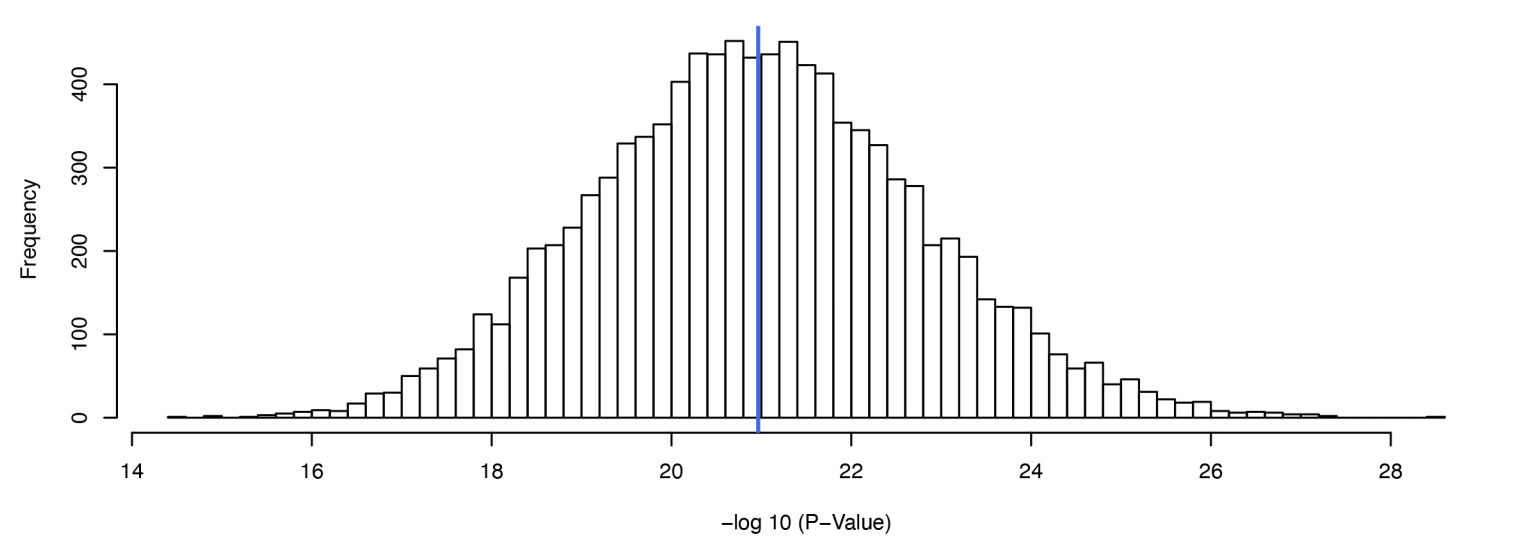

Figure S2C

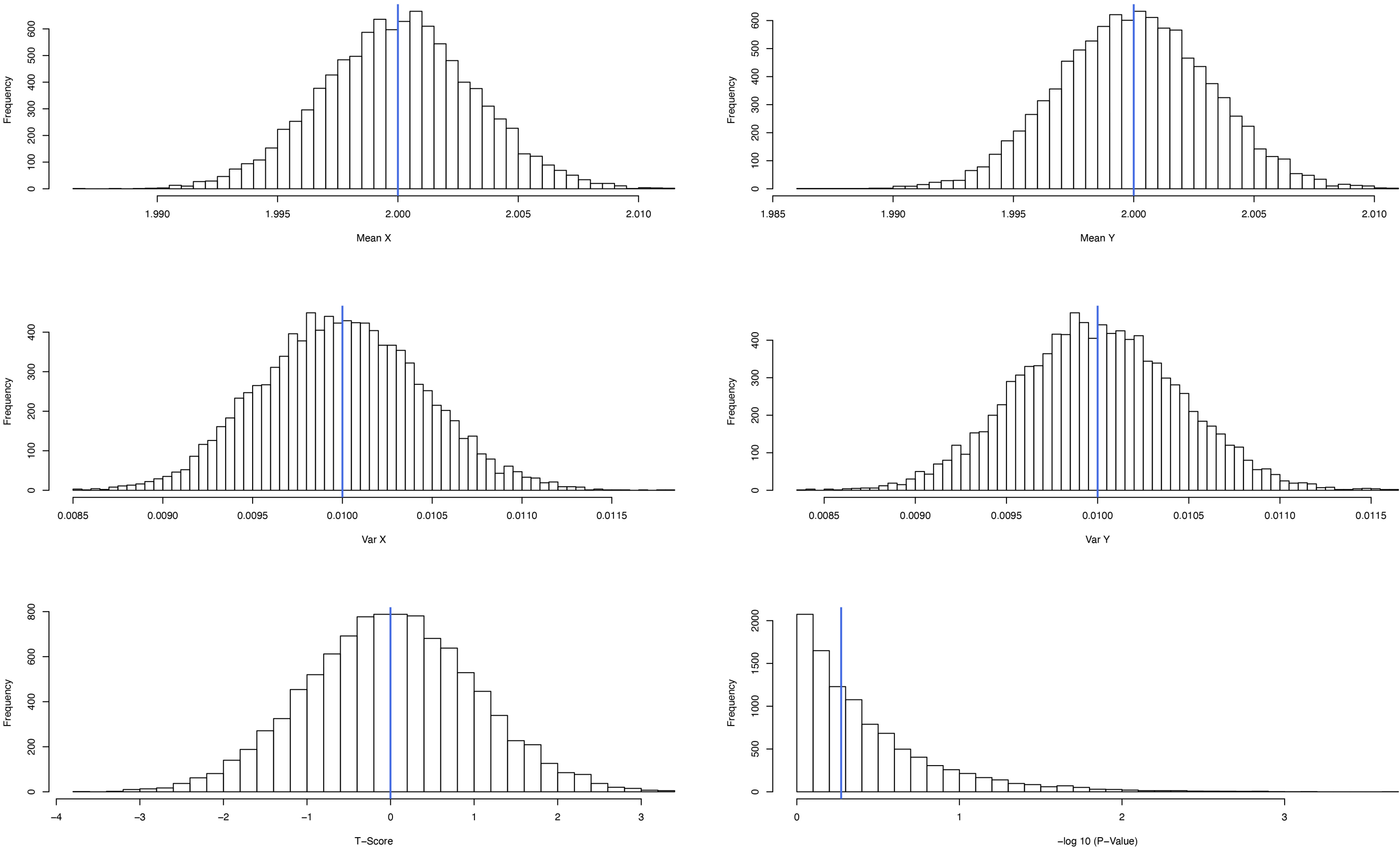

Figure S2D

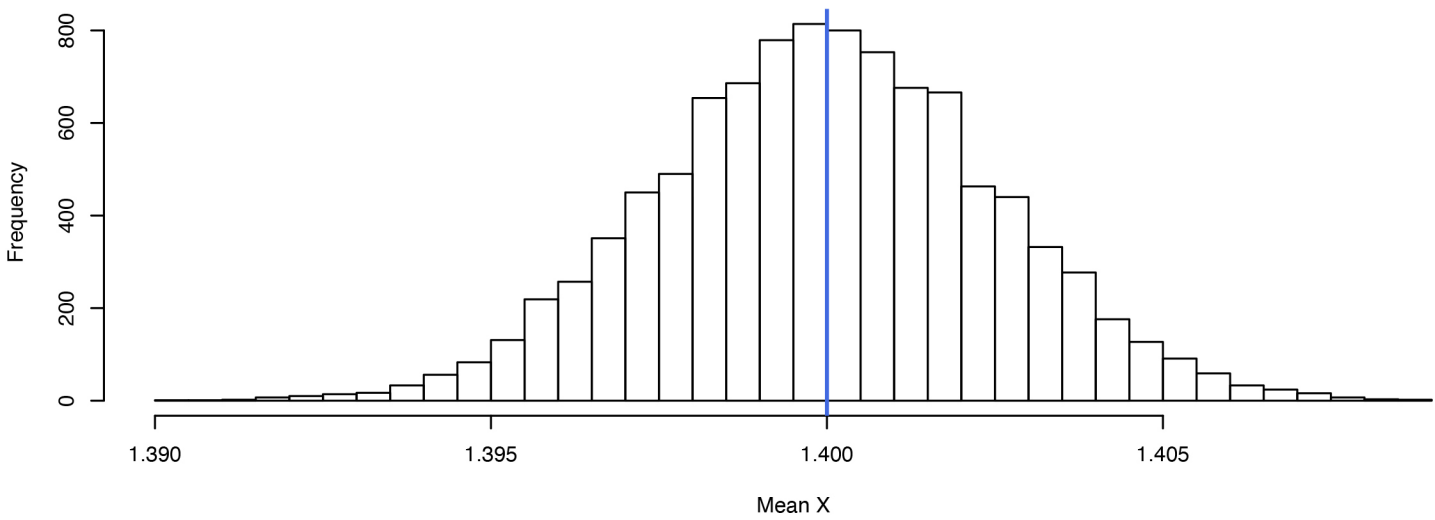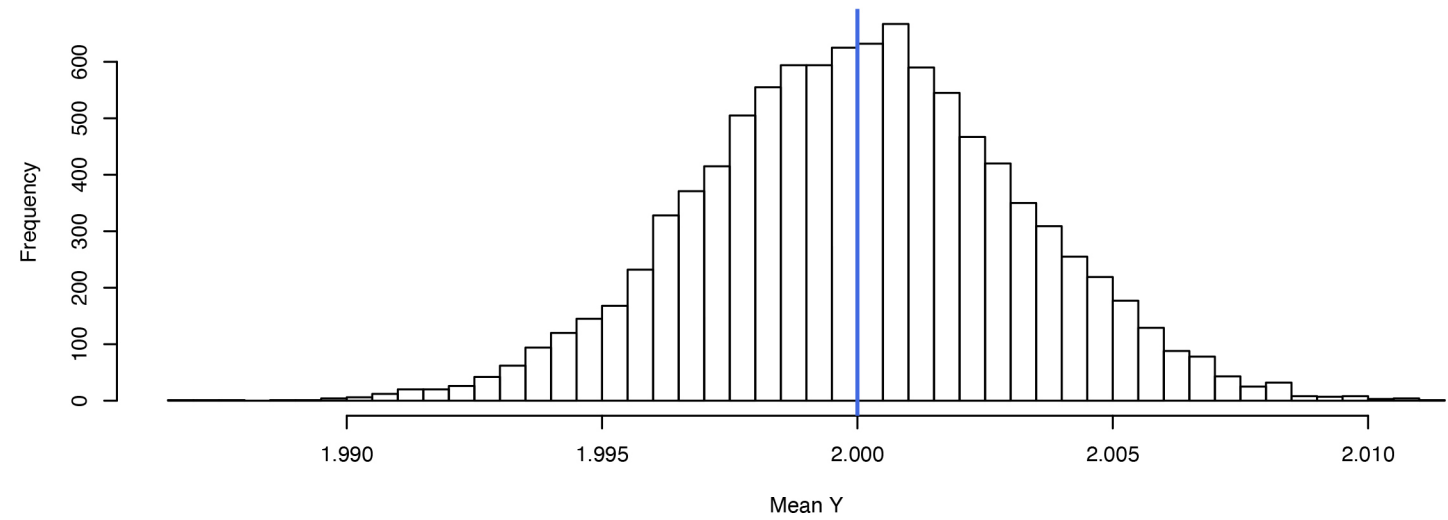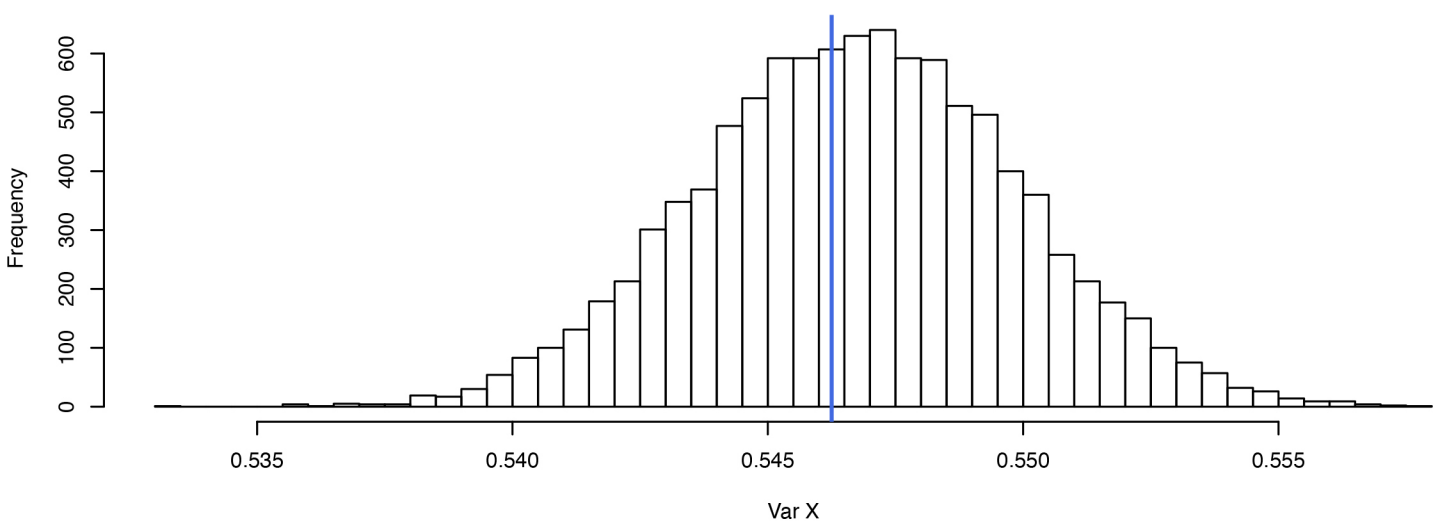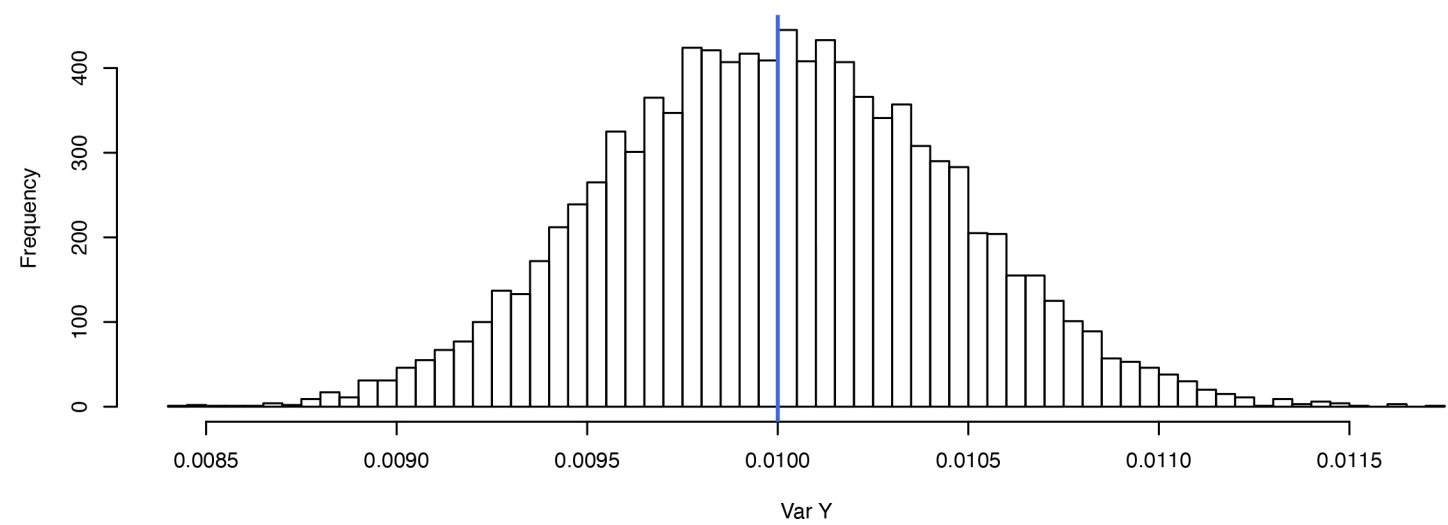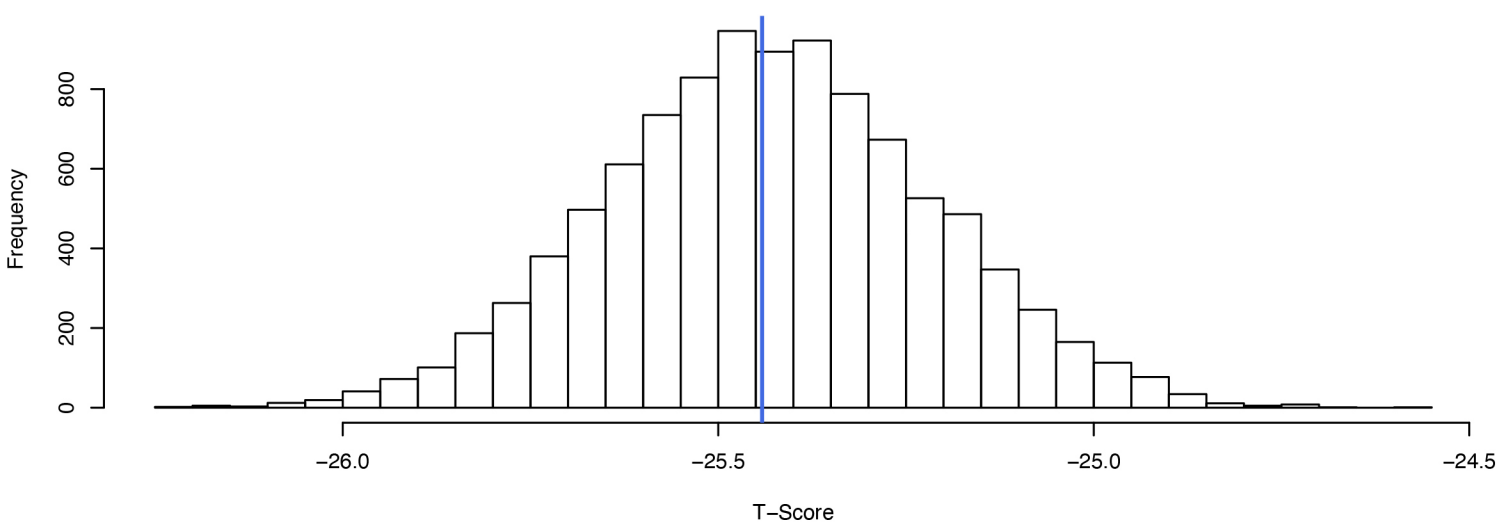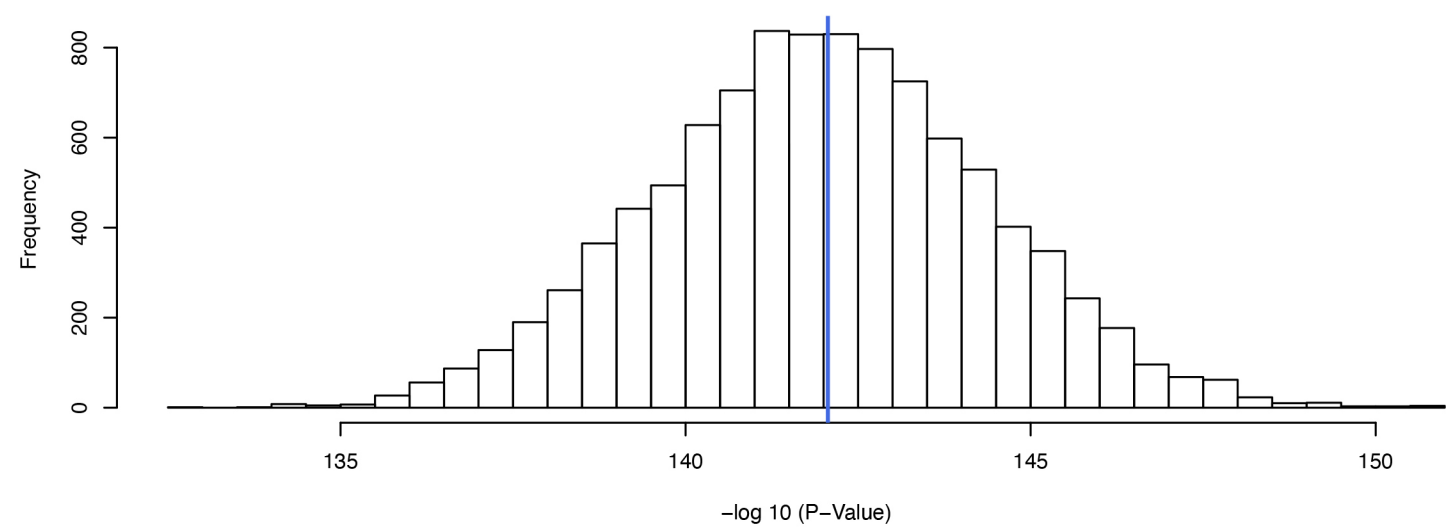

Figure S3

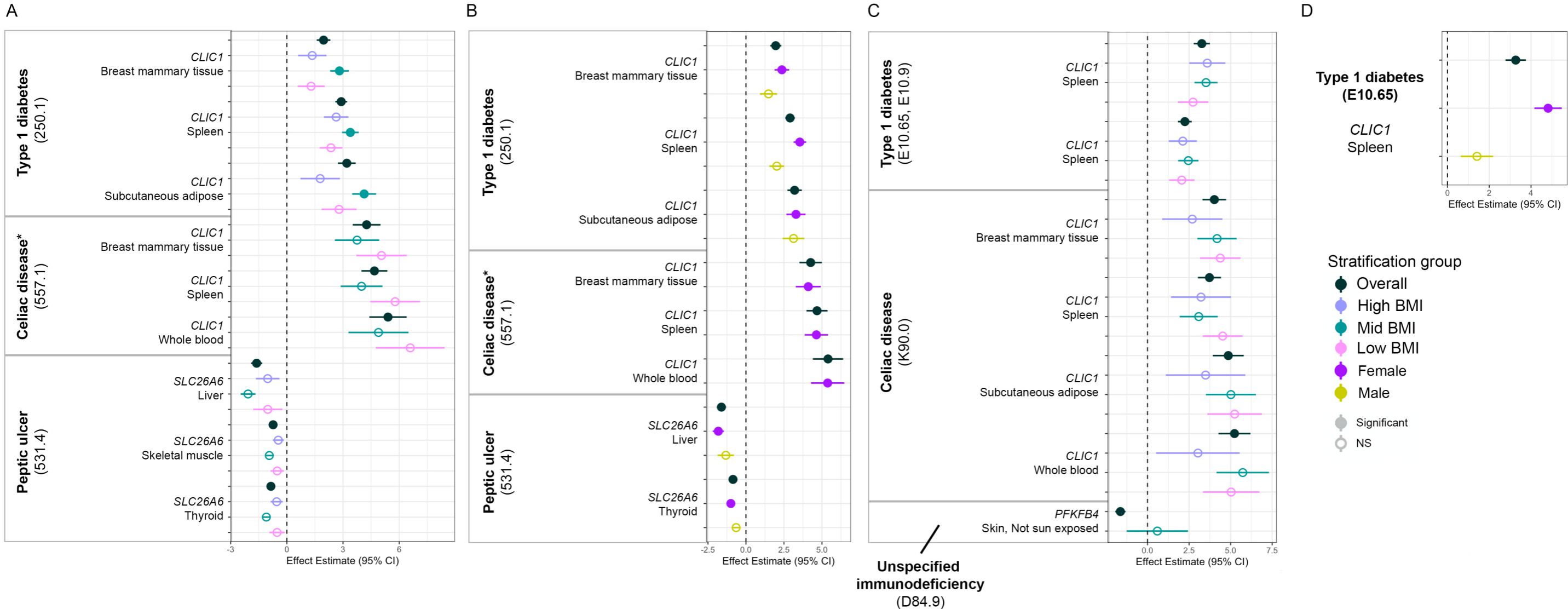

# A

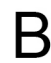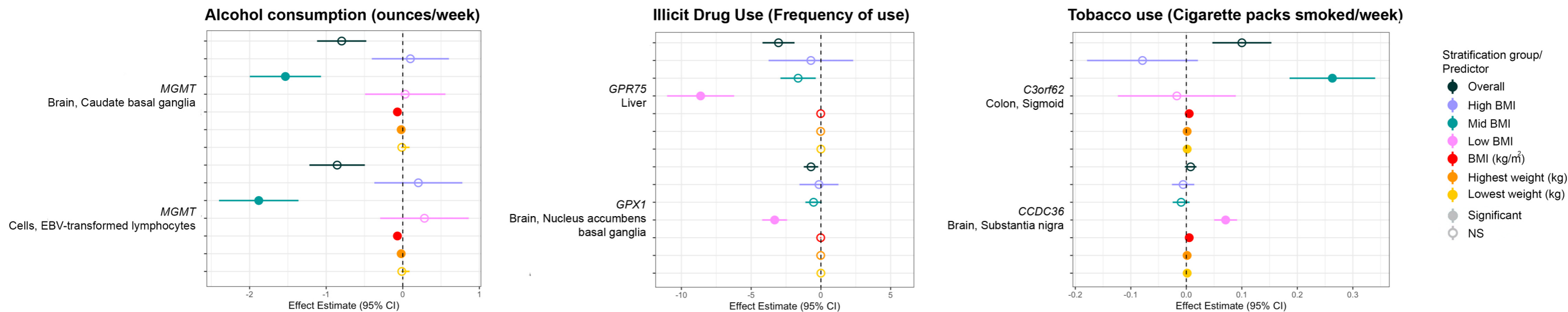

Figure S5

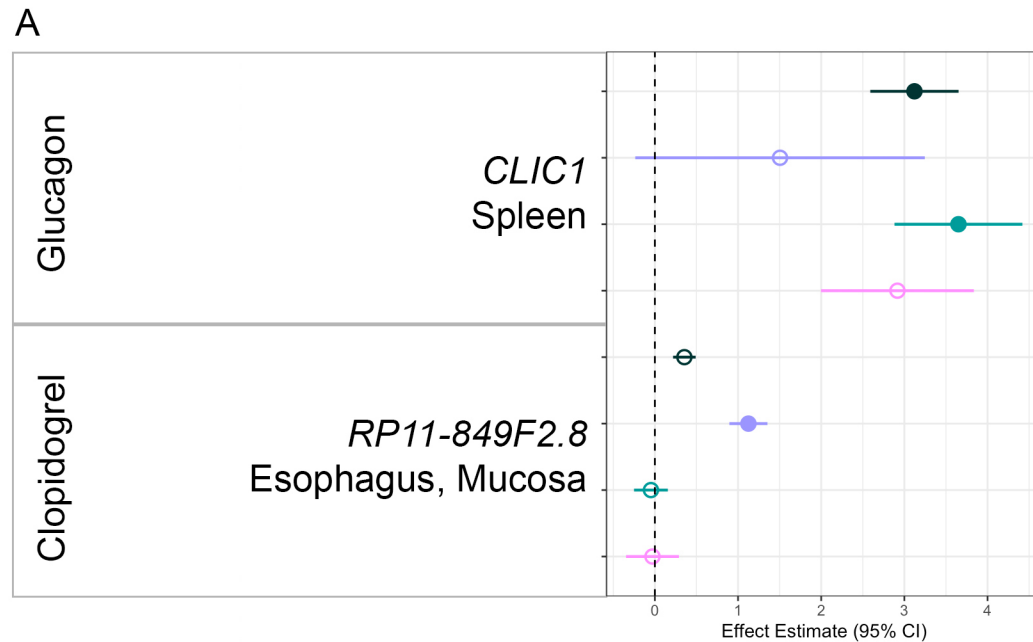

**B**

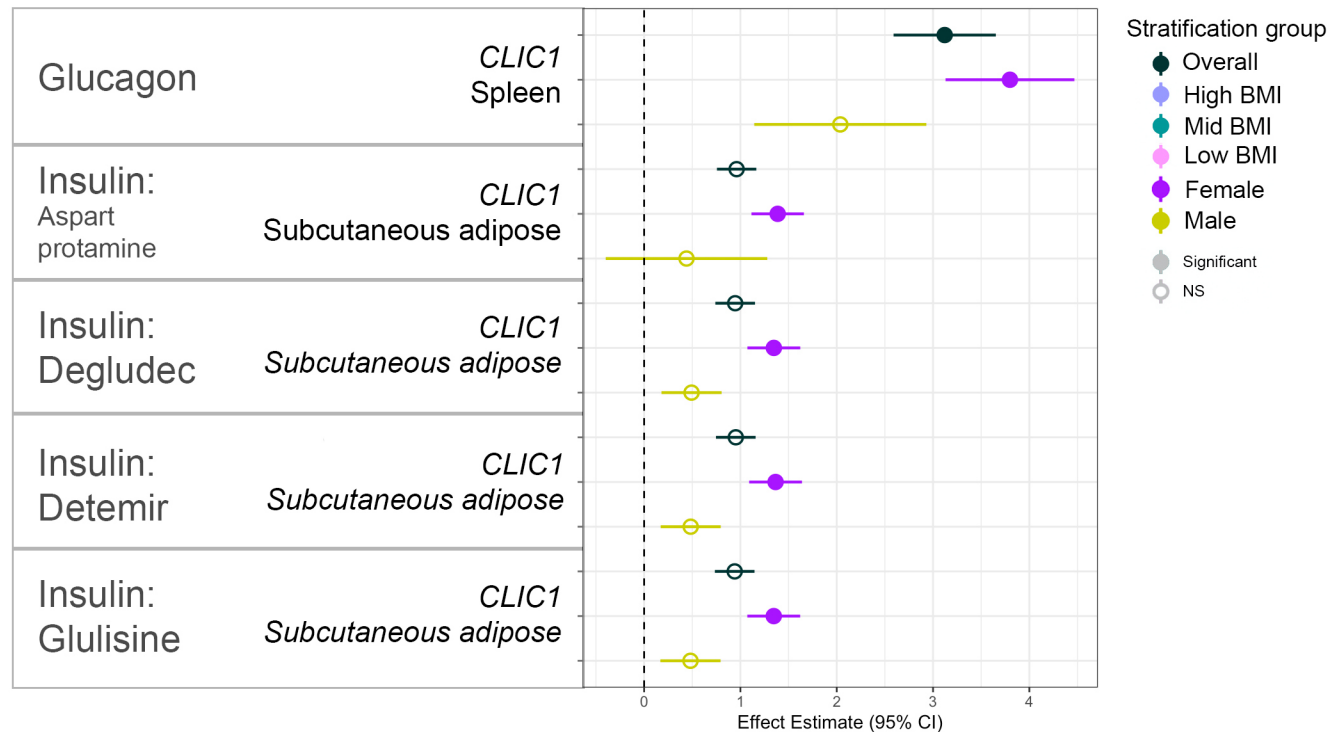

Figure S6

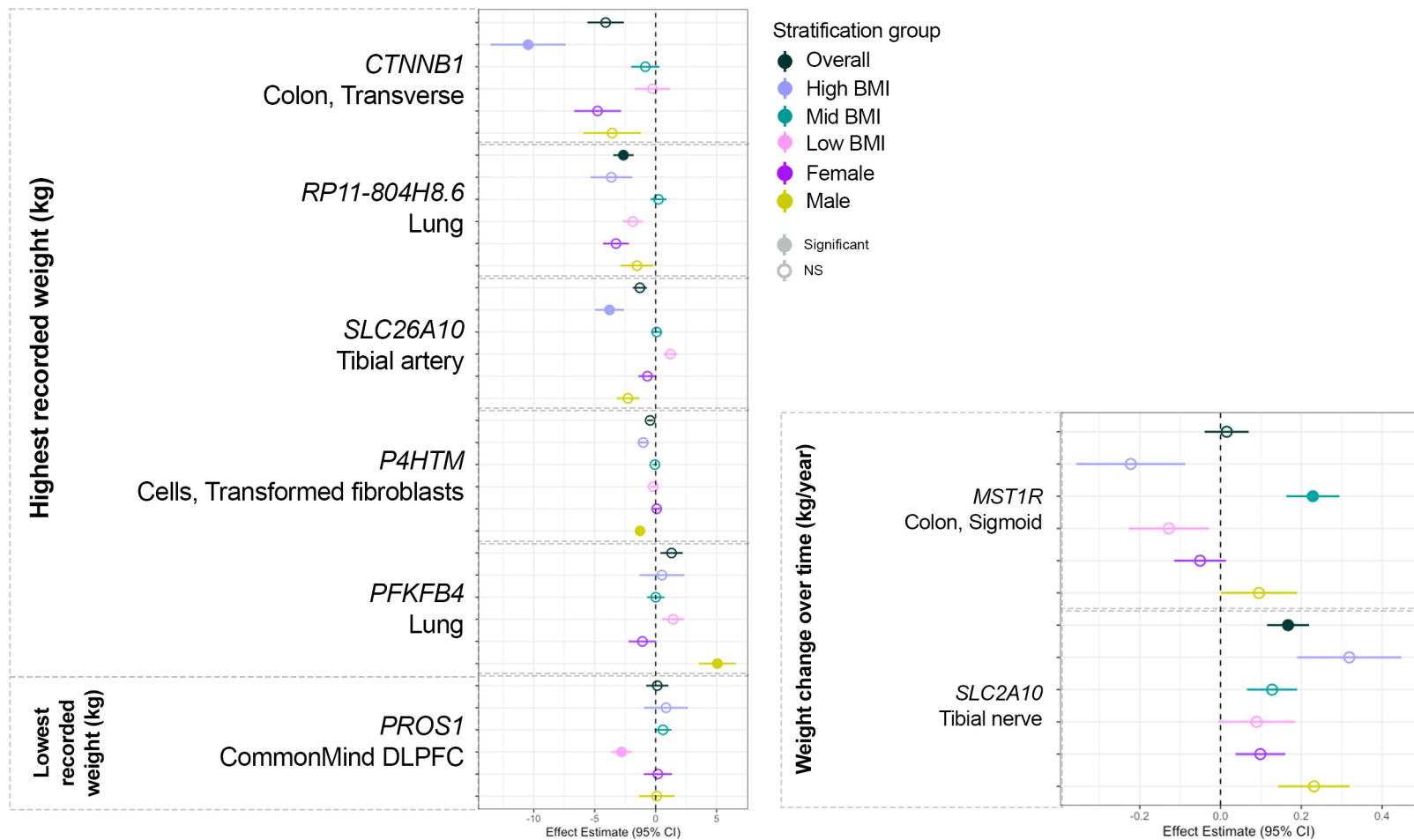

A

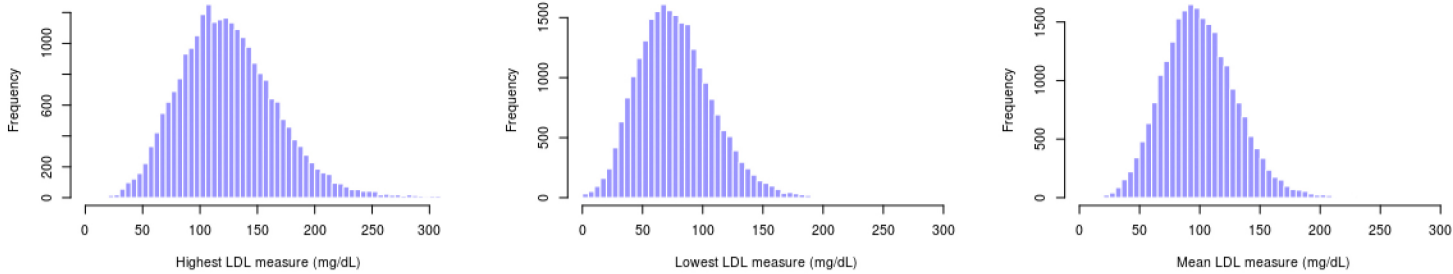

B

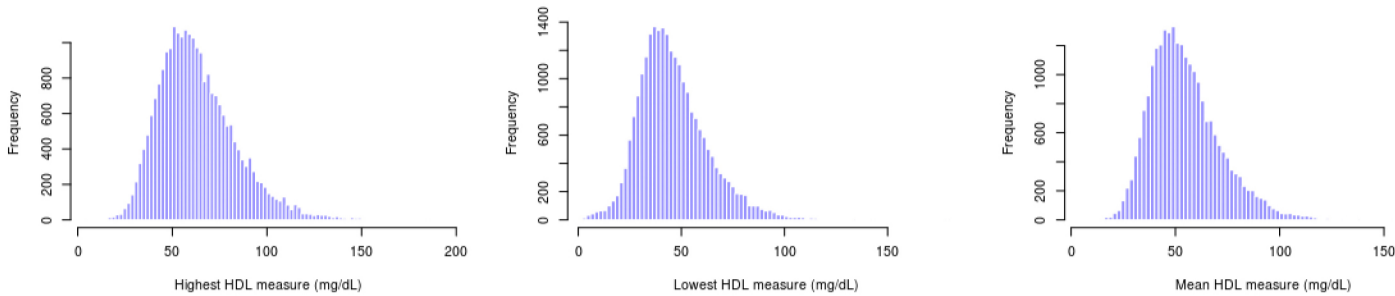

C

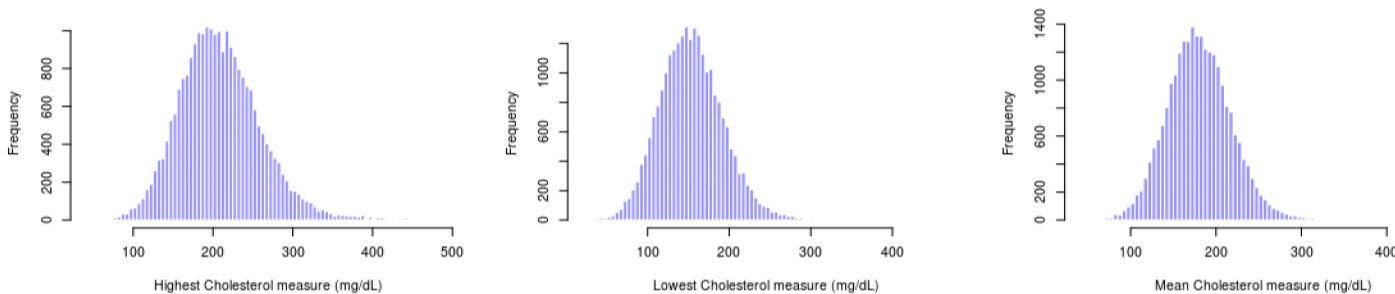

D

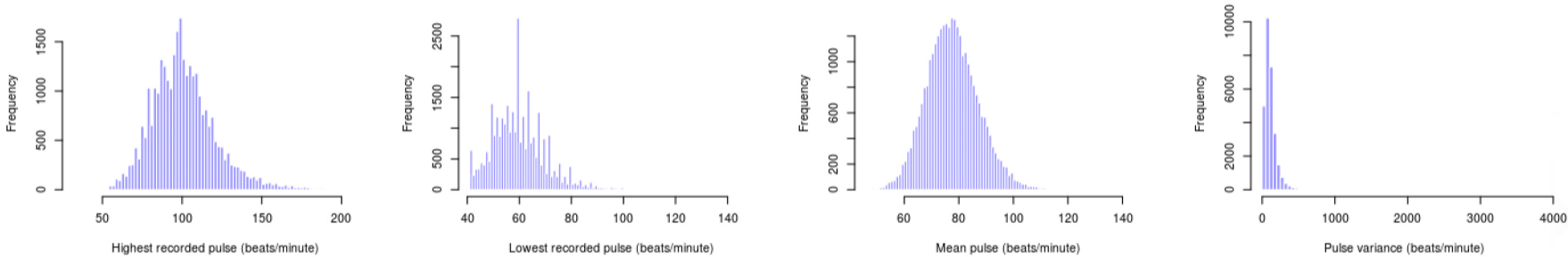

E

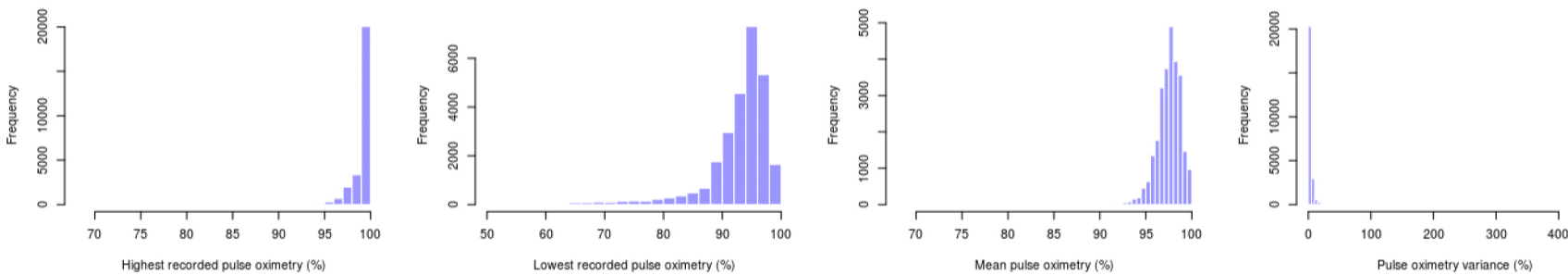

F

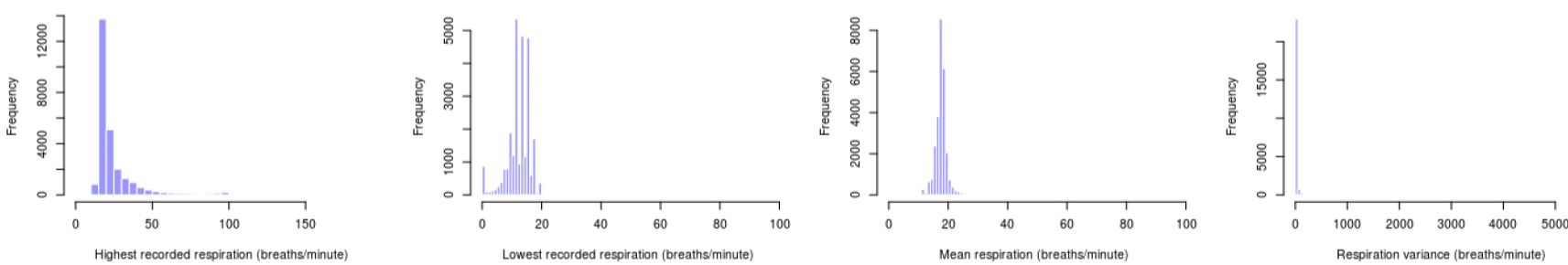

G

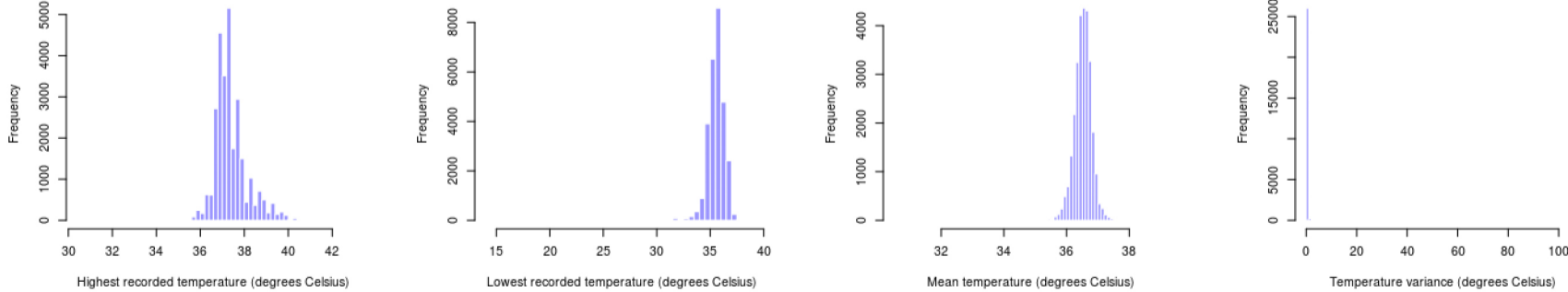

H

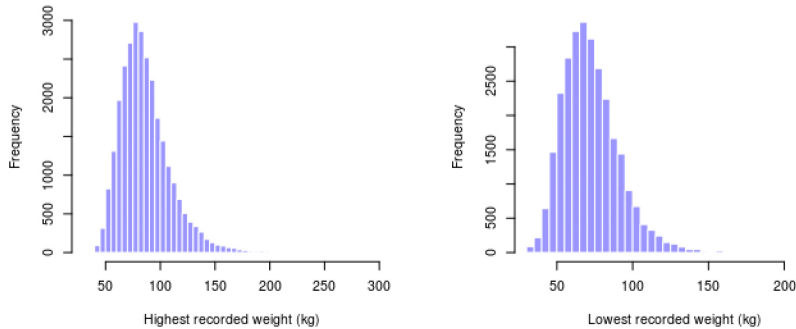

I

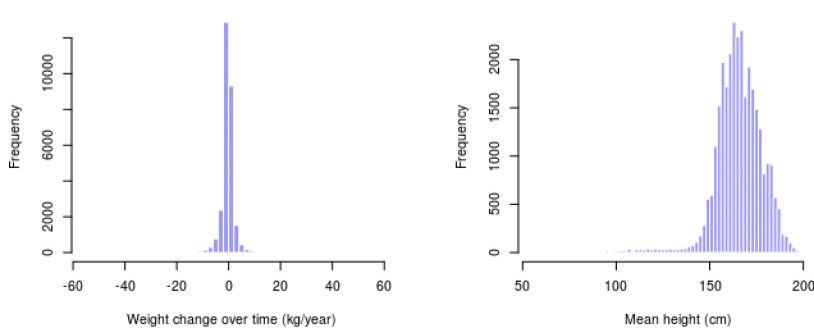

Supplement: Supplementary file 1 [file S0033291721004554sup.zip › S0033291721004554sup002.pdf]
